# Discovery of N-phenyl-(2,4-dihydroxypyrimidine-5-sulfonamido) phenylurea-based thymidylate synthase (TS) inhibitor as a novel multi-effects antitumor drugs with minimal toxicity

**DOI:** 10.1038/s41419-019-1773-0

**Published:** 2019-07-11

**Authors:** Xin-yang Li, Ting-jian Zhang, Mohamed Olounfeh Kamara, Guo-qing Lu, Hai-li Xu, De-pu Wang, Fan-hao Meng

**Affiliations:** School of Pharmacy, China Medical University, 77 Puhe Road, 110122 Shenyang, China

**Keywords:** Drug development, Drug development

## Abstract

Thymidylate synthase (TS) is a hot target for tumor chemotherapy, and its inhibitors are an essential direction for anti-tumor drug research. To our knowledge, currently, there are no reported thymidylate synthase inhibitors that could inhibit cancer cell migration. Therefore, for optimal therapeutic purposes, combines our previous reports and findings, we hope to obtain a multi-effects inhibitor. This study according to the principle of flattening we designed and synthesized 18 of *N*-phenyl-(2,4-dihydroxypyrimidine-5-sulfonamido)phenyl urea derivatives as multi-effects inhibitors. The biological evaluation results showed that target compounds could significantly inhibit the hTS enzyme, BRaf kinase and EGFR kinase activity in vitro, and most of the compounds had excellent anti-cell viability for six cancer cell lines. Notably, the candidate compound **L14e** (IC_50_ = 0.67 μM) had the superior anti-cell viability and safety to A549 and H460 cells compared with pemetrexed. Further studies had shown that **L14e** could cause G1/S phase arrest then induce intrinsic apoptosis. Transwell, western blot, and tube formation results proved that **L14e** could inhibit the activation of the EGFR signaling pathway, then ultimately achieve the purpose of inhibiting cancer cell migration and angiogenesis in cancer tissues. Furthermore, in vivo pharmacology evaluations of **L14e** showed significant antitumor activity in A549 cells xenografts with minimal toxicity. All of these results demonstrated that the **L14e** has the potential for drug discovery as a multi-effects inhibitor and provides a new reference for clinical treatment of non-small cell lung cancer.

## Introduction

Numerous chemotherapeutic drugs harm healthy tissues while treating cancer, and a single targeted drug does not achieve satisfactory therapeutic effects. Therefore, we are committed to the development of a multi-effect anti-tumor drug.

Thymidylate synthase (TS) involves in the process of DNA replication and repair^1–4^, is a critical well-recognized target for anticancer agents. The regulatory role of TS may be implicated in the synthesis of key proteins that regulate the apoptotic process^5^. In our previous studies, we reported a series of TS inhibitors based on *N*-phenyl-(2,4-dihydroxypyrimidine-5-sulfonamido)benzoyl hydrazide skeleton with IC_50_ values around 10–20 μM. And compound **10l**, the most potent one in the series, had excellent anti-proliferation ability (IC_50_ = 1.26 μM, against A549 cells), which were superior to pemetrexed in the treatment of non-small cell lung cancer (NSCLC) cell^6^.

**Fig. 1:**
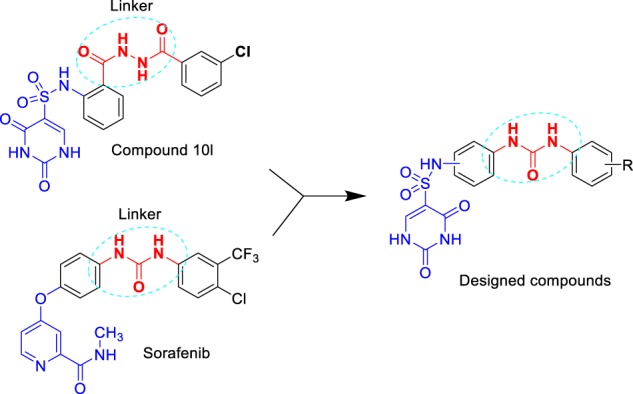
Chemical structures of selected inhibitors and design of target compounds

Many studies have shown that NSCLC is characterized by high malignancy compared with other cancers, which is closely associated with high expression of EGFR signaling pathway in NSCLC^7,8^. Evidently, dual targeting to TS and EGFR signaling pathway is an extremely attractive treatment strategy for NSCLC^9^. This preliminary conception drives us to screen the potential effects of our above-mentioned compounds on the EGFR pathway. Surprisingly, in continued studies, we found that compound **10l** could inhibit A549 cells migration at high doses, implying its potential role in the EGFR signaling pathway. The results inspired us to design TS multi-effects inhibitors with **10l** as a lead compound. Sorafenib (Fig. 1) is an oral multi-kinase inhibitor that could act on the EGFR pathway^10^. Interestingly, compound **10l** presented some structural similarity with sorafenib, which is two aryl groups linking by a 3–4 atoms linker (diacylhydrazine for the former and urea for the latter). Therefore, sorafenib could be another excellent reference compound for designing TS multi-effects inhibitors.

According to the co-crystal structure of sorafenib/BRaf, diaryl urea played a vital role in binding with wild type BRaf^11^, the lipophilic trifluoromethyl phenyl ring inserts into a hydrophobic pocket, the central phenyl ring interacts with aliphatic side chains of Lys482, Leu513, and Thr528 and the urea group forms two hydrogen bonds with the protein. In this work, we retained a basic skeleton of **10l**, and fused the diaryl urea structure of sorafenib into the molecule by replacing the diacylhydrazine fragment with a urea (Fig. 1). Then designed a series of *N*-phenyl-(2,4-dihydroxypyrimidine-5-sulfonamido) phenylurea derivatives as TS multi-effects inhibitors (Fig. 1).

In summary, we firstly synthesized a total of 18 target compounds. In vitro the inhibitory potency of the target compounds against human thymidylate synthase (hTS), BRaf kinase and EGFR kinase was evaluated, and their inhibition of the cell viability of the six cancer cells was further examined in vitro. In the subsequent studies, we investigated the proliferation, migration and apoptosis of A549 cells and H460 cells using MTT assay, transwell migration assay, and flow cytometry, respectively. Besides, the expression levels of apoptotic proteins and EGFR-related proteins were measured. In vivo pharmacological evaluation of **L14e** was performed in A549 tumor xenografts and toxicity studies.

## Results

### Chemistry

**Scheme 1:**
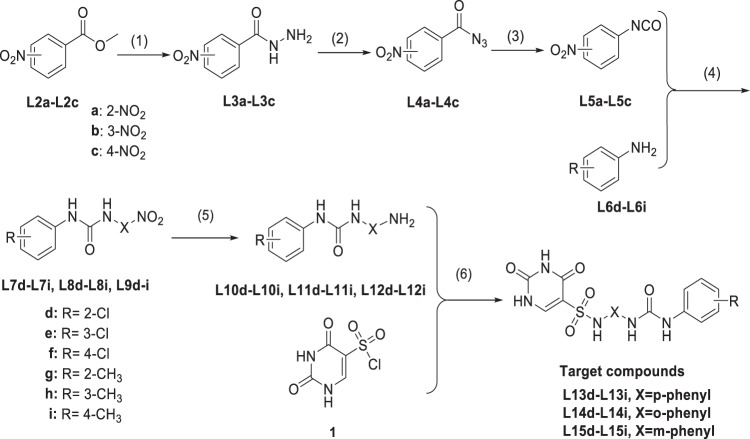
Reagents and condition: (1) N_2_H_4_·H_2_O, CH_3_OH, 80 °C; (2) NaNO_2_, HCl, CH_2_Cl_2_, 0 °C; (3) CH_2_Cl_2_, 80 °C; (4) CH_2_Cl_2_, Corresponding substituted aniline, 80 °C; (5) Zn, NH_4_Cl, 25 °C; (6) DMF, Pyridine, 25 °C

The chemical synthesis of N-phenyl-(2,4-dihydroxypyrimidine-5-sulfonamido)phenyl urea derivatives (**L13d-L13i**, **L14d-L14i**, and **L15d-L15i**) was carried out by the synthetic method illustrated in Scheme 1. Preparation of 2,4-dihydroxypyrimidine-5-sulfonylchloride(**1**) was done according to the reported method in our past work^6^.

Compounds **L3a-L3c** underwent diazonium reaction in the presence of NaNO_2_ and dilute hydrochloric acid to obtain compound **L4a-L4c** at 0 °C. Then, the compound **L4a-L4c** was subjected to Curtius rearrangement reaction at 80 °C to obtain compounds **L5a-L5c** which was also then reacted with the corresponding substituted aniline(**L6d-L6i**) to obtain a corresponding substituted nitrophenylurea compound (**L7d-L7i**, **L8d-L8i**, and **L9d-L9i**). Also, the nitro group was reduced in the presence of Zn and NH_4_Cl at 25 °C. Finally, the corresponding substituted aminophenyl urea compounds (**L10d-L10i**, **L11d-L11i**, and **L12d-L12i**) were reacted with uracil under pyridine as an acid binding agent at 25 °C to obtain the target compounds (**L13d-L13i**, **L14d-L14i**, and **L15d-L15i**). Here, the post-treatment of the target compound involved the Hinsberg reaction and was purified according to the method reported by Gris^12^. The overall synthetic route is environmentally friendly, has no violent reaction, and the yield is as high as 70% or more and provides guarantee for mass production in the industry. Furthermore, when purifying the target compound, we found that the solubility of the compound at a pH of 8.0 to 9.0 is from 0.1 to 0.5 g/mL.

### In vitro enzyme activity assay and molecular docking

In vitro human TS (hTS) enzyme activity, BRaf kinase and EGFR kinase assays studies on 18 target compounds synthesized, the inhibition of enzymes by target compounds were summarized in Table 1. From the results, the inhibition efficiency of compound **L14e** was significantly improved after structural modification. Moreover, the overall inhibition of TS by the compounds were superior to the inhibitory effect on the BRaf kinase and were superior to the inhibition of EGFR kinase. Among them, compound **L14e** had the most effective inhibitory effect on these enzymes. Since the active sites of tyrosine kinases were universal (both ATP binding sites), we had molecular docking of hit compound **L14e** with TS and BRaf kinase to study their mode of action.

**Table 1:** IC_50_ values of compounds, PTX and Sorafenib against human TS (hTS), BRaf and EGFR kinase

Compounds	IC_50_^a^ (µM)			Compounds	IC_50_ (µM)		
	hTS	BRaf	EGFR		hTS	BRaf	EGFR
**L13d**	2.36	2.39	4.22	**L14h**	3.52	3.61	4.28
**L13e**	4.55	3.64	4.41	**L14i**	6.98	5.45	>10
**L13f**	6.30	3.59	4.65	**L15d**	3.81	4.09	7.65
**L13g**	9.25	>10	>10	**L15e**	2.17	2.96	4.37
**L13h**	4.57	3.53	3.99	**L15f**	1.55	2.68	5.54
**L13i**	7.39	4.91	5.48	**L15g**	>10	>10	>10
**L14d**	1.91	1.62	2.39	**L15h**	7.16	6.38	7.10
**L14e**	**1.06**	**1.09**	**1.92**	**L15i**	8.3	7.23	8.67
**L14f**	2.12	1.71	2.23	**PTX**	2.71	–	–
**L14g**	6.47	7.41	>10	**10l**	1.36	1.67	2.71
**Sorafenib**	–	0.57	8.12				

^a^All IC_50_ values (μM) are averages from triplicate assays

**Fig. 2:**
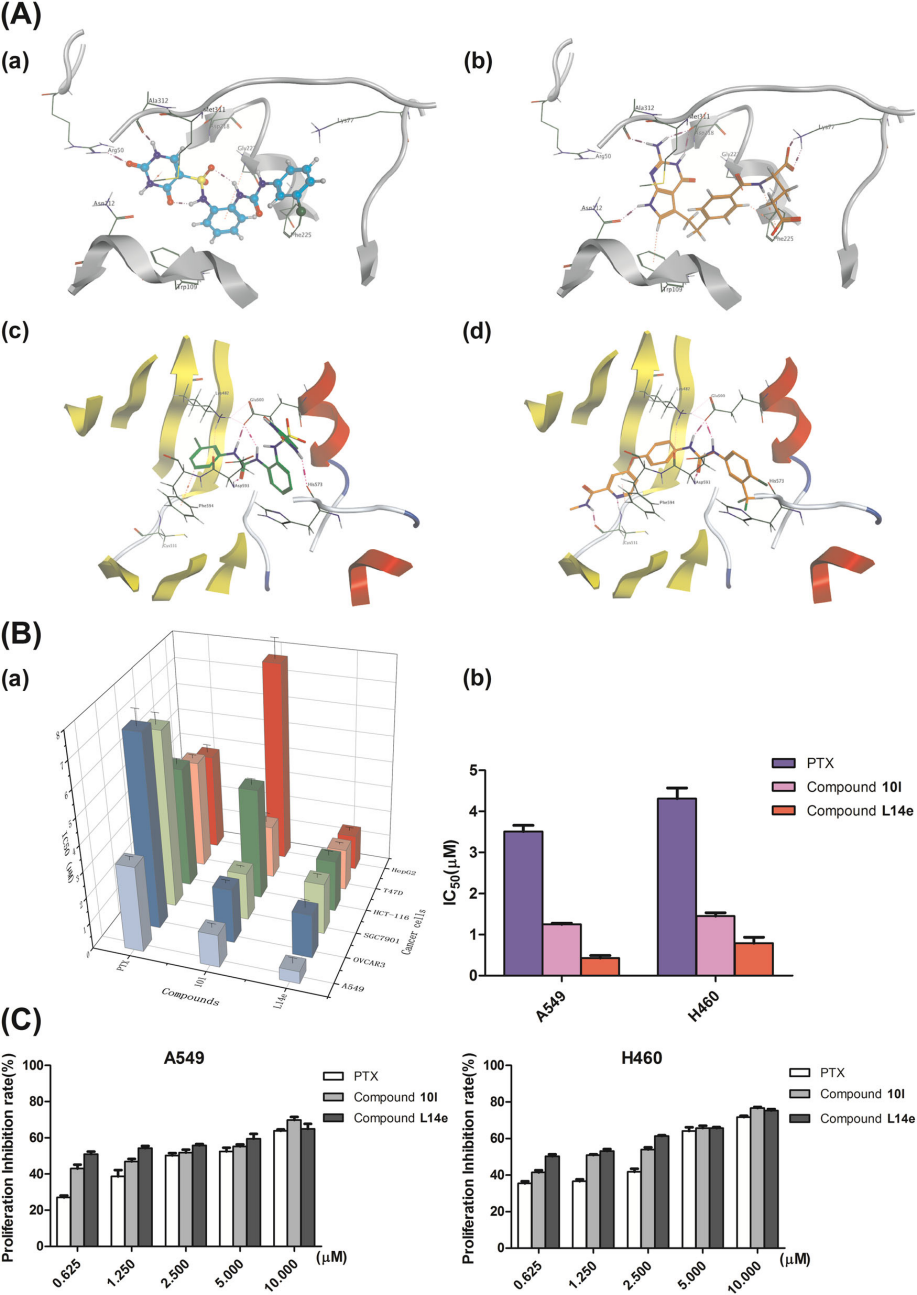
A Docking poses of compound L14e (a, c), PTX (b) and Sorafenib (d) within the protein binding pocket; B, C Comparison of compound L14e with PTX and compound 10l for: inhibiting the proliferation of six cancer cells and inhibiting the proliferation of A549 and H460 cells at the similar concentrations

To rationalize the structure-activity relationships (SARs) observed in this study and to foresee the possible interactions of the synthesized compounds with TS, molecular modeling simulations of **L14e** in the binding pocket of TS were performed with MOE (Molecular Operating Environment, version 2016.08, Chemical Computing Group Inc., Canada) software. The crystal structure of TS in complex with PTX (PDB code: 1JUJ) was adopted in the docking calculations. According to the docking results (Fig. 2A), compound **L14e** located at the similar position to that of the PTX crystal structure (Fig. 2A(a)), the uracil fragment formed two strong H-bonds with Arg50 and Ala312 residues, and the phenyl group attached to the sulfonamide group formed an H-Arene conjugate with Gly222. Although the H-bond interactions between Lys77 and glutamate residue of PTX were missing, an alternate π-π stack interaction between Phe225 and terminal benzyl ring were conducted to remain the binding affinity (Fig. 2A(b)). This interaction could explain the differences in TS inhibitory activities between **L14e** and PTX.

Furthermore, to investigate the possible interaction of the compound with tyrosine kinase, the crystal structure of human BRaf in complex with sorafenib (PDB code: 1UWH) was adopted in the docking calculations. **L14e** overlaped with the bis- uracil ring of sorafenib and forms a similar hydrogen bond. For example, the urea fragment formed three strong H bonds with the Glu500 and Asp593 residues (Fig. 2A(c)). Since the sulfonamide fragment and the urea fragment were in the ortho position of the benzene ring, the molecular structure was folded (not stretched), so that the urea fragment of **L14e** cannot penetrate the active pocket like the pyridine fragment of sorafenib (Fig. 2A(d)). In contrast, the pyrimidine fragment is on the outside of the active pocket and forms a hydrogen bond with His573. In addition, the terminal benzyl ring of **L14e** undergoes an alternating π-π stacking interaction with Phe594 to maintain binding in the active pocket. These interactions supplied a unique ligand-receptor binding pattern that distinguishes sorafenib and also reasonably explains the BRaf inhibitory activity of **L14e**.

### In vitro anti-proliferative activity studies and SAR study of compounds

Taking PTX, Sorafenib and compound **10l** as a reference compound, the target compounds (**L13d-L13i**, **L14d-L14i**, and **L15d-L15i**) were evaluated for the anti-proliferative against six cancer cell lines: A549, OVCAR-3, SGC7901, HCT-116, MDA-MB-231 and HepG2 by 3-(4,5-dimethyl-2-thiazolyl)-2,5-diphenyltetrazolium bromide (MTT) assay. The results were expressed as IC_50_ values and summarized in Table 2, and the values were the average of at least three independent experiments. As shown in Table 2, 95% of the target compounds showed excellent ability to inhibit cell viability against A549, OVCAR-3, SGC7901, HCT116, MDA-MB-231, and HepG2. Primarily, they proved to have high inhibitory potency against A549 cells when compared with the other five cancer cells (Fig. 2B).

**Table 2:** IC_50_ values of compounds and PTX against A549, OVCAR-3, SGC7901, HCT-116, MDA-MB-231, and HepG2 cell lines

Compounds	IC_50_^a^ (µM)					
	A549	OVCAR-3	SGC7901	HCT-116	MDA-MB-231	HepG2
**L13d**	2.01 ± 0.14	1.73 ± 0.44	5.30 ± 0.28	4.13 ± 0.16	>10	6.56 ± 0.15
**L13e**	0.95 ± 0.05	1.05 ± 0.42	3.38 ± 0.18	2.46 ± 0.06	2.48 ± 1.46	2.81 ± 0.16
**L13f**	0.99 ± 0.07	8.04 ± 0.32	3.98 ± 0.05	5.53 ± 0.25	8.89 ± 2.42	4.35 ± 0.45
**L13g**	4.68 ± 0.11	2.68 ± 0.38	6.39 ± 0.19	4.75 ± 0.07	>10	9.16 ± 0.12
**L13h**	1.85 ± 0.13	9.56 ± 0.94	6.35 ± 0.52	5.46 ± 1.21	>10	5.83 ± 0.18
**L13i**	3.48 ± 0.13	>10	4.92 ± 0.23	7.98 ± 0.61	>10	8.25 ± 0.25
**L14d**	3.29 ± 0.30	7.27 ± 1.23	5.22 ± 0.18	4.96 ± 0.63	6.71 ± 0.18	2.20 ± 0.06
**L14e**	**0.67** ± **0.22**	**1.68** ± **0.31**	**1.95** ± **0.15**	**1.98** ± **0.18**	**2.60** ± **0.08**	**1.36** ± **0.13**
**L14f**	1.38 ± 0.27	6.52 ± 0.50	3.98 ± 0.33	4.22 ± 0.31	4.54 ± 0.41	1.62 ± 0.10
**L14g**	2.09 ± 0.72	>10	5.40 ± 0.09	4.82 ± 0.19	>10	5.59 ± 0.64
**L14h**	1.45 ± 0.06	7.30 ± 0.68	3.30 ± 0.15	5.39 ± 0.41	7.16 ± 0.90	>10
**L14i**	1.37 ± 0.45	>10	5.13 ± 0.44	5.62 ± 0.61	7.30 ± 0.10	5.72 ± 0.21
**L15d**	1.87 ± 0.12	6.30 ± 0.25	5.25 ± 0.81	2.65 ± 0.33	2.04 ± 0.29	4.97 ± 0.36
**L15e**	0.87 ± 0.28	1.19 ± 0.49	2.58 ± 0.43	3.30 ± 0.07	3.13 ± 0.09	2.29 ± 0.29
**L15f**	1.01 ± 0.11	1.49 ± 0.28	4.28 ± 0.67	7.61 ± 0.04	3.20 ± 0.62	3.07 ± 0.29
**L15g**	8.75 ± 0.61	>10	7.04 ± 0.87	6.41 ± 1.83	>10	7.66 ± 0.78
**L15h**	1.46 ± 0.50	5.98 ± 0.60	4.78 ± 0.62	6.15 ± 0.53	6.41 ± 0.41	5.97 ± 0.21
**L15i**	4.18 ± 0.46	>10	6.96 ± 1.53	9.50 ± 1.60	9.06 ± 1.13	6.96 ± 0.59
**PTX**	3.29 ± 0.15	7.35 ± 0.60	6.96 ± 0.43	4.64 ± 0.22	4.18 ± 0.14	3.73 ± 0. 31
**10l** ^**b**^	1.26 ± 0.16	2.08 ± 0.12	1.82 ± 0.08	2.27 ± 0.23	2.04 ± 0.37	7.66 ± 0.77
**Sorafenib**	>10	>10	>10	>10	>10	8.21 ± 0.34

^a^Inhibitory effect was reported as an IC_50_ value (IC_50_ = Mean ± SD). From MTT assay after 24h of treatment; the values were average from at least 3 independent experiments

Further, based on the results of preliminary screening of MTT, the structure-activity relationship (SAR) of the compounds was analyzed. When X was replaced by *o*-phenyl, R was replaced by 3-Cl; the anti-proliferative activity of the compound (**L14e**) was better than the replacement by 4-Cl (**L14f**), wherein 4-Cl replaced the R, however, the anti-proliferative activity of the compound was better than replaced by 2-Cl (**L14d**). This was consistent with when X is replaced by *m*-phenyl or *p*-phenyl. Simultaneously, when R was replaced by 3-CH_3_ (**L14h**) the anti-proliferative activity of the compound was better than replaced by 4-CH_3_ (**L14i**), and R was replaced by 4-CH_3_ the anti-proliferative activity of the compound was better than replaced by 2-CH_3_ (**L14g**), Whether X was replaced by *m*-, *p*-, or *o*-phenyl. In summary, our results indicated that the introduction of a urea group increases the activity of the target compound, and the introduction of an electron withdrawing group was superior to the electron donating group and compound **L14e** had the best anti-proliferative activity (IC_50_ = 0.67 ± 0.22 μM) than the other compounds.

### L14e had more anticancer activity and selectivity index than PTX and compound 10l and had more potential of medicinal research significance on A549 and H460

The most common types of NSCLC are adenocarcinoma, large cell carcinoma and squamous cell carcinoma^13^. The candidate compound **L14e** was subjected to MTT assay on representative cell lines: A549, H460 cells, and SK-MES-1. The result, as shown in Fig. 2B(b), **L14e** was significantly more inhibitory to the proliferation of A549 and H460 cells than PTX and compound **10l**. All of them did not inhibit SK-MES-1 cells.

Further comparative analysis, as shown in Fig. 2C, we found that the inhibition rate of **L14e** was better than PTX at each concentration point, and moreover **L14e** had a higher inhibitory capacity at low concentrations compared to compound **10l**, indicating that the compound **L14e** has an excellent anti-proliferative potency on A549 cells and H460 cells than compound **10l** and PTX. Also, **L14e** had a higher selectivity index (SI) than PTX, its SI even higher than compound **10l**, which was shown in Table 3. This result also indicated that **L14e** had high inhibition of A549 and H460 at low concentrations and low injury to healthy cells, which made it possible to become a potential agent.

**Table 3:** Selectivity index of compound L14e, compound 10l and PTX against HPAEpiC, IOSE80, GES-1, HCoEpiC, HTB-125, LO2 cell lines

Compound	SI (Selectivity index)^a^					
	HPAEpiC	IOSE80	GES-1	HCoEpiC	HTB-125	LO2
**L14e**	19.79	8.79	8.58	9.40	10.39	12.58
**10l**	16.56	11.90	14.25	4.09	5.26	12.57
**PTX**	7.71	3.56	3.25	4.91	6.70	8.23

^a^SI = CC_50_/IC_50_

In summary, compound **L14e** had stronger ability to inhibit cell viability and higher selectivity index than both PTX and **10l** and had more potential of medicinal research significance on NSCLC, and this study mainly focused on the inhibitory effect of compound **L14e** on A549 and H460 cells.

### L14e could arrest the cell cycle in G1/S phase then affect the proliferation of A549 and H460 cells

**Fig. 3:**
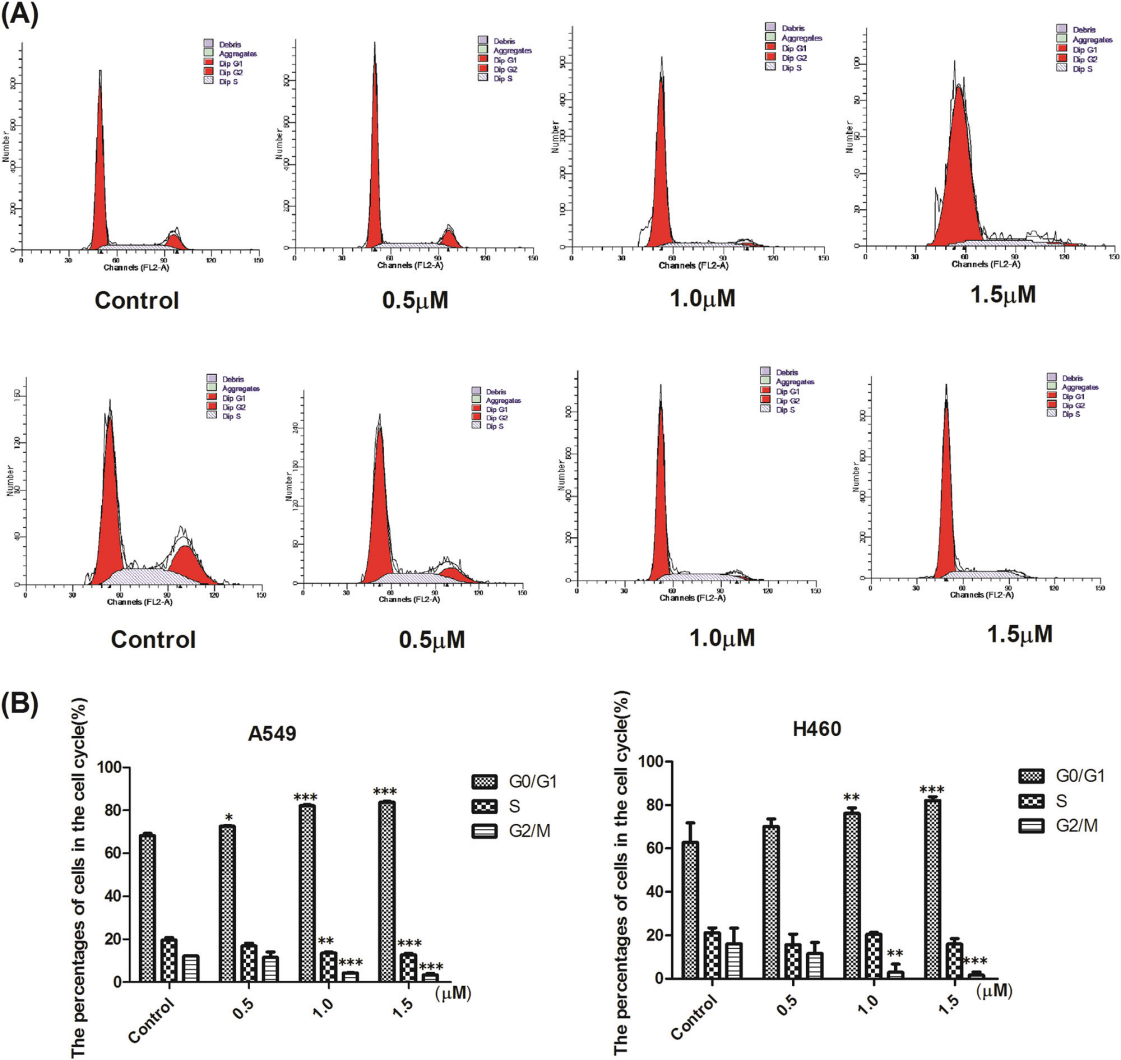
a, b Effects of compound L14e on cell cycle were evidenced by PI staining and FACS analysis. Three independent experiments were performed, each column represented the mean ± SD of triplicate determinations (*P < 0.05; **P < 0.01 and ***P < 0.001. Compound **L14e** -treated group VS control). **L14e** was able to further inhibit cell proliferation by inducing apoptosis in A549 and H460 cells

TS is a crucial enzyme in the DNA synthesis stage. When it is inhibited, first to show cell cycle arrest at G1/S phase^14^. According to TS assays results, compounds could significantly inhibit TS activity, and MTT assays results showed that **L14e** has nearly five times higher anti-proliferative potency than PTX to A549 and H460 cells. To verify that the compound **L14e**’s inhibition of cell viability is associated with cell cycle arrest, A549 cells and H460 cells were treated with different concentrations of compound **L14e** (0, 0.5, 1.0, 1.5 μM) for 24 h, then collected and stained with PI staining^15^. Flow cytometry show (Fig. 3), as the concentration of the compound increases, the cells in the G0/G1 phase increase, the cells in the S phase and the G2/M phase decrease. Eventually, the proliferation of cancer cells was inhibited by the G1/S phase arrest of the compound **L14e**.

These results demonstrated that compound **L14e** could arrest the cell cycle in the G1/S phase, then affect A549 and H460 cell proliferation. And the result is significantly different (*P* < 0.001).

### L14e could further inhibit cell proliferation by inducing apoptosis in A549 and H460 cells

**Fig. 4:**
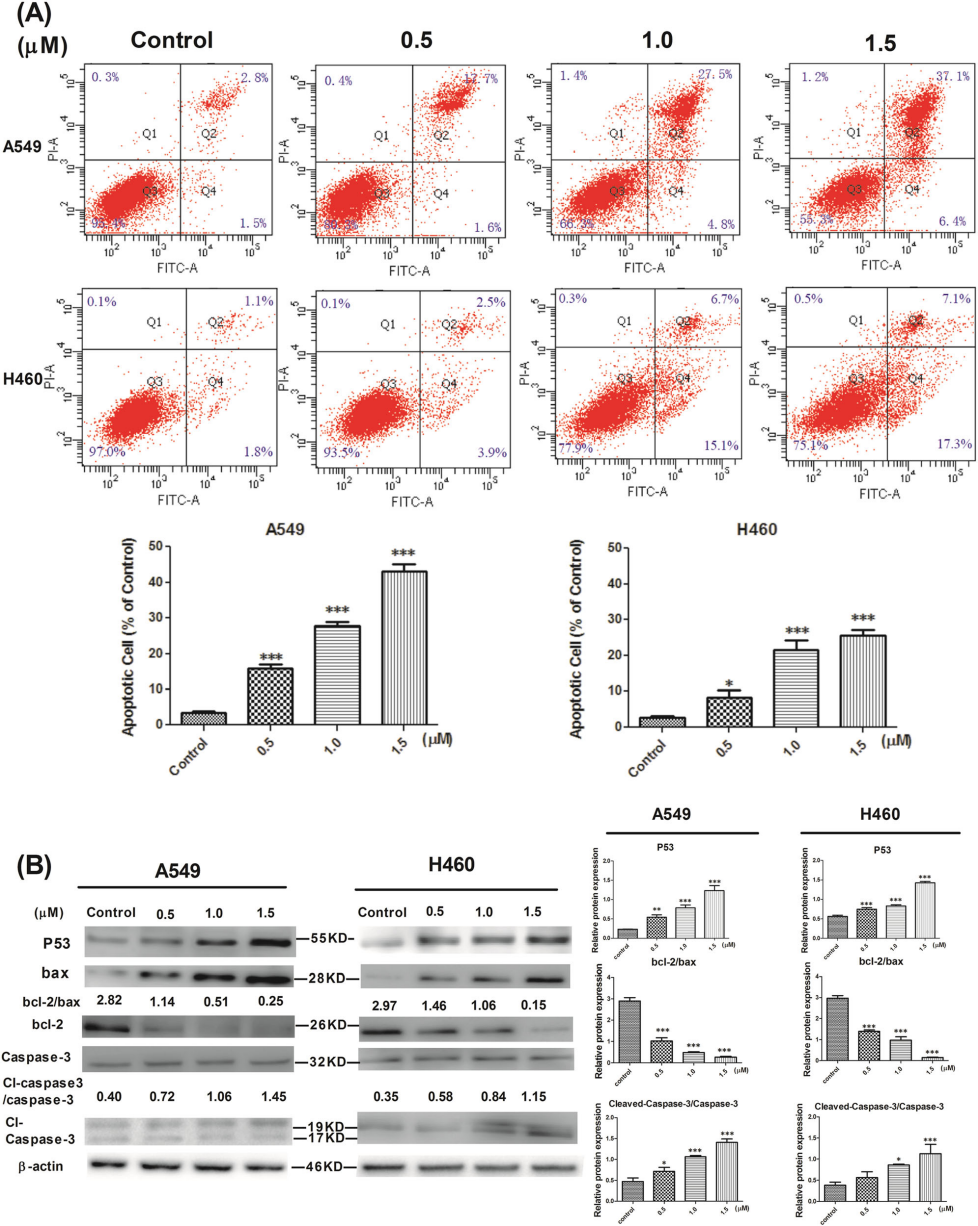
a Compound L14e induces apoptosis in A549 and H460. Effects of compound L14e on cell death were evidenced by Annexin V-FITC/PI double staining and FACS analysis. Apoptotic cells were Annexin V [+] and PI [−], late apoptotic cells were Annexin V [+] and PI [+], nectotic cells were Annexin V [−] and PI [+] and living cells were Annexin V [−] and PI [−]. Three independent experiments were performed, each column represented the mean ± SD of triplicate determinations (*P < 0.05; **P < 0.01 and ***P < 0.001. Compound L14e -treated group VS control). **b** Effect of compound L14e on the apoptotic protein expression of P53, caspase-3, bcl-2/bax and cleaved caspase-3 in A549 and H460. The levels of P53, caspase-3, bcl-2/bax and cleaved caspase-3 proteins were analyzed by western blot. Relative protein expression of bcl-2/bax, cleaved-caspase-3/caspase-3, and P53 in A549 and H460 with or without compound L14e. Each column represented the mean ± SD of triplicate determinations (*P < 0.05; **P < 0.01 and ***P < 0.001. Compound L14e -treated group VS control)

Most chemotherapeutic drugs eventually induce apoptosis in cancer cells when they inhibit cell cycle progression, thereby achieving anti-tumor effects^16^. Therefore, to determine whether the cell cycle arrest caused by the compound **L14e** could ultimately induce apoptosis, A549 cells and H460 cells were treated with different concentrations of compound **L14e** (0, 0.5, 1.0, 1.5 μM) for 24 h, then collected and stained with Annexin V/PI staining^15^. Flow cytometry showed that the apoptotic rate of A549 cells was 4.3%, 19.3%, 23.3%, and 43.5% for each concentration respectively (Fig. 4a). With the increase in concentration, the apoptotic rate increased, of which the late apoptosis rates were 2.8%, 17.7%, 27.5%, and 37.1%. However, the apoptotic rate of H460 cells was 2.9%, 6.4%, 21.8%, and 24.4% for each concentration respectively. With the increase in concentration, the apoptotic rate increased, of which the early apoptosis rates were 1.8%, 3.9%, 15.1%, and 17.3%.

It was suggested that compound **L14e** could induce apoptosis of A549 cells and H460 cells, eventually inhibited cell proliferation. And the result is significantly different (*P* < 0.001).

### Effect of L14e on the apoptotic protein expression of P53, caspase-3, bcl-2/bax, and cleaved caspase-3 in A549 and H460 cells

According to previous studies, TS was a key enzyme in DNA replication and repair^17^. Flow cytometry results showed that the compound **L14e** was able to cause G1/S phase arrest in cancer cells. However, when the DNA structure was damaged, which will activate the p53 pathway cause mitochondria apoptosis^18,19^. The P53 gene is one of tumor suppressor genes, which was activated that bax proteins will be motivated to over express^20^. Bax is a pro-apoptotic protein in the bcl-2 gene family that is homologous to bcl-2 which is anti-apoptotic protein. When bax is over express, it can antagonize the protective effect of bcl-2, and the expression of bcl-2 is decreased, then increase the expression of cleaved caspase-3, eventually, induce apoptosis^21^. Therefore, when P53 expression increases and the ratio of bcl-2/bax decreased and activated caspase-3 increases, the apoptosis increased^22,23^.

To further investigate the expression of P53 and the expression of bcl-2, as well as the activation of caspase-3, A549 cells and H460 cells were treated with different concentrations of (0, 0.5, 1.0, 1.5 μM) compound **L14e** for 24 h and western blot analysis was carried out. It was observed that over expression of P53, up-regulation of bax and down-regulation of bcl-2, as well as the activity of caspase-3 which in turn caused an increased in cleaved caspase-3 protein levels as shown in Fig. 4b. And the result is significantly different (*P* < 0.001).

### L14e could inhibit the migration of A549 and H460

In vitro kinase assay showed that the compound **L14e** has a stronger potential to inhibit EGFR activity than Sorafenib. To verify the multi-effects of the compound **L14e**, we investigated the effects of **L14e** on A549 and H460 cells migration by wound healing assays and transwell assays.

**Fig. 5:**
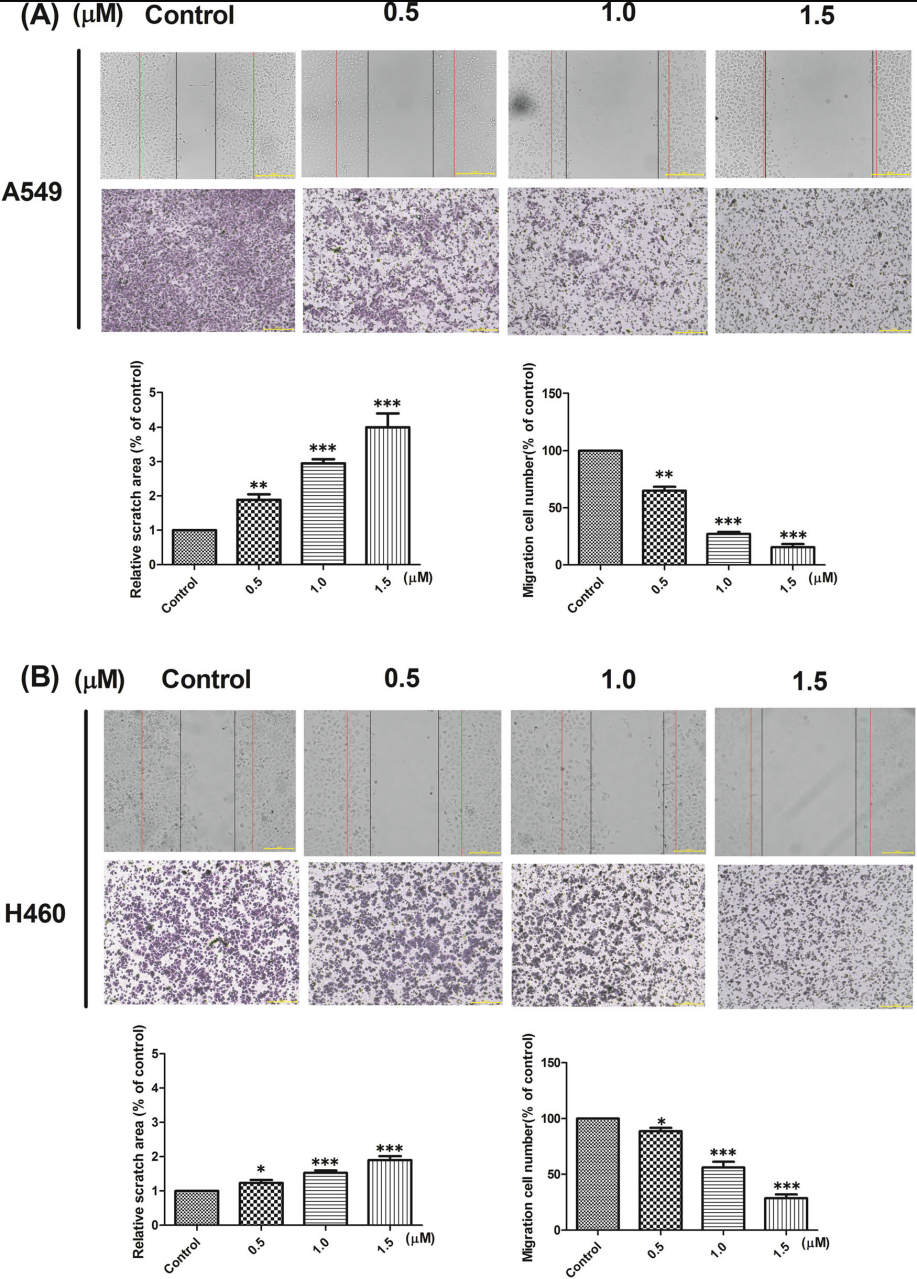
**a**, **b** L14e inhibited the migration of A549 and H460. The red line is the initial scratch and the black line is the cell migration edge. Cells were seeded into the upper part of transwell chamber. The lower compartments were filled with 500 μL of RPMI 1640 supplemented with 10% bovine serum albumin. After cells were incubated for 24 h, the migrated cells on the lower surface of the membrane were quantified by counting the number of cells in ten random fields per membrane and expressed as cells/fields (mean ± SD). The scale bars indicate 100 μm. (*P < 0.05; **P < 0.01, and ***P < 0.001. Compound **L14e** -treated group VS control)

The results showed that the **L14e** significantly inhibited the migration of A549 cells and H460 cells in a dose-dependent manner (Fig. 5). The inhibition of the migration ability of A549 cells was particularly prominent. These all revealed that the **L14e** has a significant inhibitory effect on the migration of cancer cells. And the result is significantly different (*P* < 0.001).

### Effect of L14e on the activation of p-EGFR, p-AKT, and p-ERK1/2 in A549 cells

**Fig. 6:**
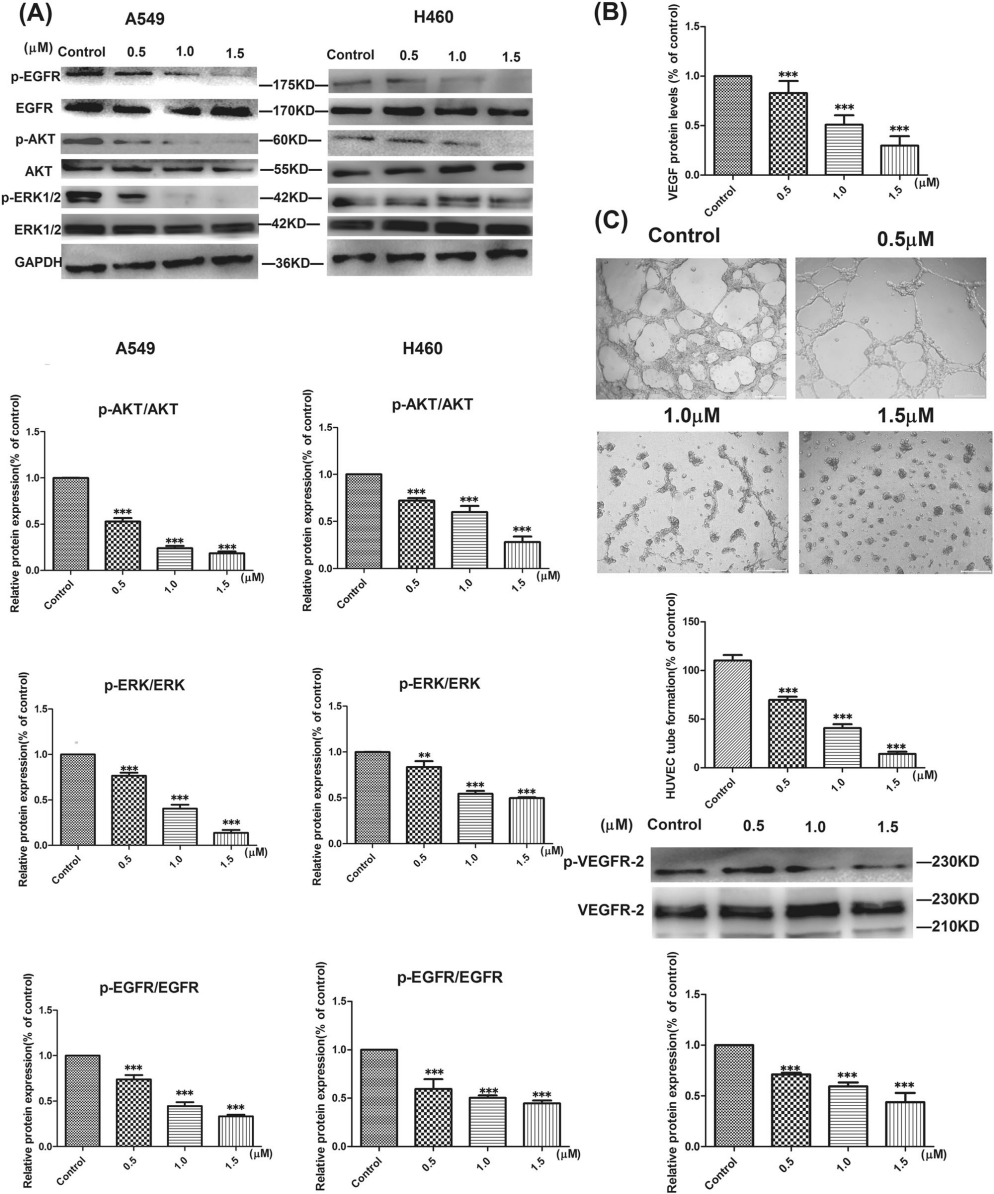
**a** Effect of compound L14e on EGFR signaling pathway. Cells were treated in the presence of EGF (50 ng/ml) for 10 min at 37 °C prior to lysis. (i) Relative protein expression of p-EGFR/EGFR, p-AKT/AKT, p-ERK/ERK in the A549 cells; (ii) Relative protein expression of p-EGFR/EGFR, p-AKT/AKT, p-ERK/ERK in the H460 cells. **b** Effect of compound **L14e** on the secretion of VEGF in A549 cells**. c L14e** inhibited tube formation of HUVECs and inhibited the activation of VEGFR-2. HUVECs were seeded in Matrigel-coated well with culture supernatant of A549 cells treated with different concentrations of compound **L14e** (0, 0.5, 1.0, 1.5 μM) and incubated for 24 h. The tube formation of control HUVECs was normalized as 100%. Each value represents the mean ± SD of three independent experiments. The scale bars indicate 100μm. (*P < 0.05; **P < 0.01 and ***P < 0.001. Compound **L14e** -treated group VS control). The scale bars indicate 100 μm

Numerous studies proved that the migration capacity of lung cancer is associated with high activation of EGFR signaling pathway^24,25^. To investigate the mechanism by which the compound L14e inhibits migration is associated with signaling regulation of the EGFR signaling pathway, cells were treated with different concentrations of **L14e** (0, 0.5, 1.0, 1.5 μM) for 24 h and western blot analysis was carried out. As the results are shown in Fig. 6a, **L14e** significantly reduced the expression of phosphorylated EGFR (p-EGFR, Tyr^1068^) and AKT (p-AKT, Ser^473^) in A549 and H460 cells. And down-regulate the expression of downstream p-ERK1/2. This effect was particularly pronounced in A549 cells.

Therefore, this result revealed that the inhibition of migration of the compound **L14e** was directly related to the down-regulation of EGFR/AKT and EGFR/ERK signaling pathway and the result is significantly different (*P* < 0.001).

### L14e could reduce the secretion of VEGF in A549 cells and inhibit the tube formation of HUVECs

Studies had shown that inhibition of EGFR signaling pathway directly affects the secretion of VEGF in cancer cells^26,27^. Moreover, when VEGF secretion is reduced, it directly affects the formation of blood vessels in tumor tissues^28^. Consequently, to multiple verify the inhibitory effect of the **L14e** on the EGFR signaling pathway, we detect the levels of VEGF secreted by tumor cells.

Since the inhibitory effect of the compound on A549 cells was stronger than that of H460, the A549 cells were treated with different concentrations of **L14e**. Taking the supernatant of the cells to detect the change of VEGF content by ELISA assays, and the effect of the supernatant on the formation of endothelial cells was detected. The ELISA result showed that the **L14e** could significantly reduce the secretion of VEGF in A549 cells (Fig. 6b). Furthermore, the **L14e** inhibited tube formation by inhibiting the expression of phosphorylated VEGFR-2 (p-VEGFR-2, Tyr^1175^) in a dose-dependent manner.

Summarize the results of inhibition of transwell and tube formation, proved that **L14e** could inhibit the activation of the EGFR signaling pathway and ultimately achieve the purpose of inhibiting cancer cell migration and angiogenesis in cancer tissues. And the result is significantly different (*P* < 0.001).

### L14e displays minimal toxicity and significantly inhibited tumor growth in vivo

**Fig. 7:**
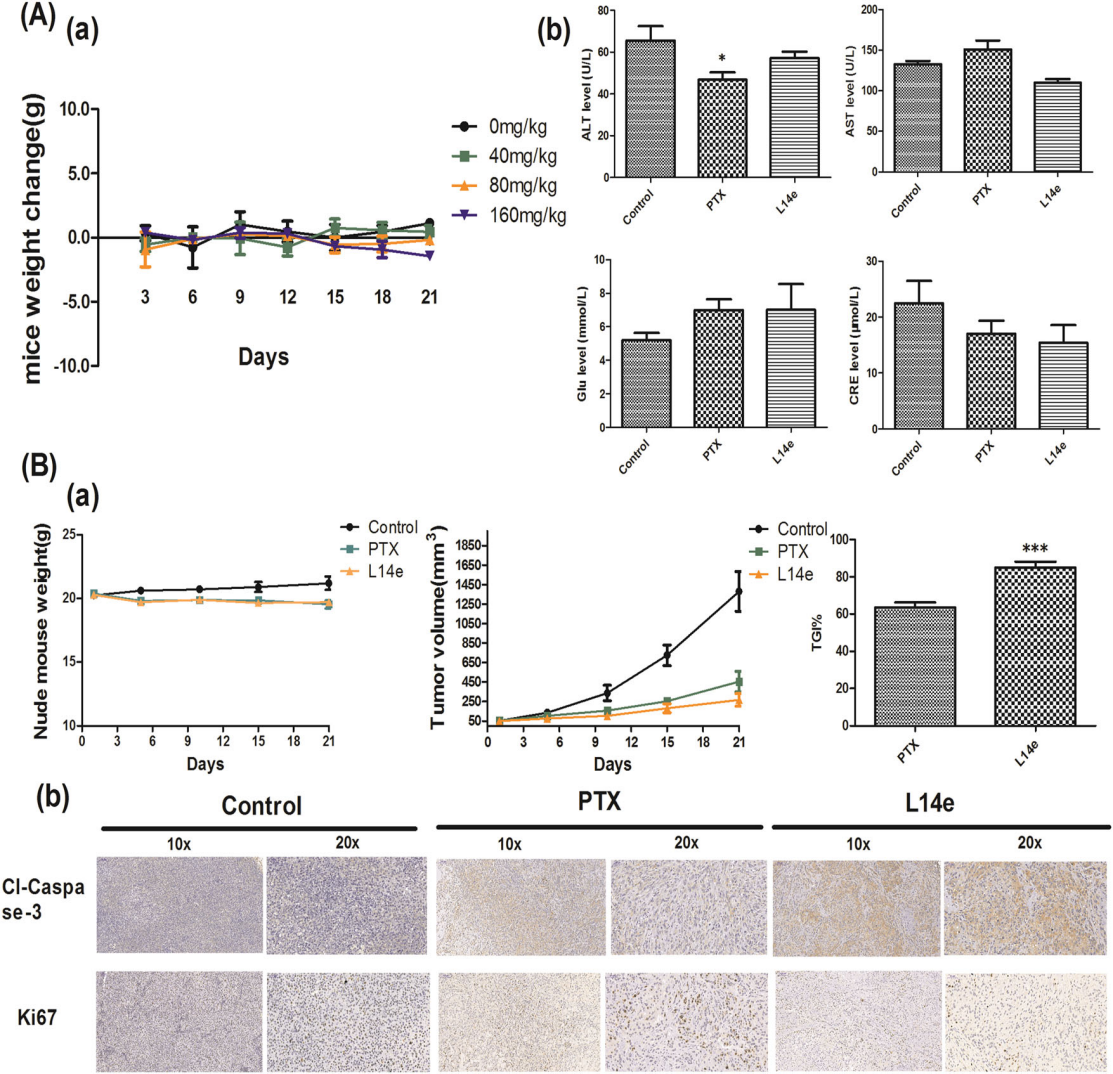
Minimal toxicity of L14e in vivo and inhibitory effects of the L14e on the growth of xenograft tumors. **Aa** Changes in body weight induced by treatment with **L14e** in mice (*n* = 5). **Ab** The level of ALT, AST, Glu and CRE in the serum. **Ba** Body weight changes and tumor volume changes in PTX- or **L14e**-treated mice (*n* = 5); The Tumor Growth Inhibition value (TGI) of **L14e**. (*P < 0.05; **P < 0.01 and ***P < 0.001. Compound **L14e** -treated group VS PTX- treated group). **Bb** IHC analyses for evaluating the expression of cleaved-caspase-3 and Ki67 in the tumors from PTX- or **L14e-**treated nude mice

All in vitro studies had shown that the **L14e** has an excellent anti-proliferative potency on A549 cells and has the potential to become an agent. Therefore, we studied the toxicity and efficacy of the compound **L14e** in vivo. To investigate the toxic effects of the compound **L14e** in vivo, mice were injected with different concentrations of compound **L14e** (0, 40, 80, 160 mg/kg) for 3 weeks^29^. The results showed that changes in the body weight of **L14e**-treated mice were minimal compared with those of control mice (Fig. 7A(a)). Simultaneously, the serum levels of aspartate aminotransferase (AST), alanine aminotransferase (ALT), blood glucose (GLU) and creatinine (CER) were evaluated (Fig. 7A(b)). The levels of these indicators in the serum collected from **L14e**-treated mice were within the reference ranges (and no significant changes). The histopathological changes in liver, lung, brain, and kidney from mice treated with PTX or **L14e** were evaluated by using HE-stained section of the tissues; the results showed that there were no visible pathological changes in each organ(the results were supplied in supplemental materials). These results indicated that **L14e** has minimal toxicity to mice.

Further research that evaluated the antitumor effect of the **L14e** in A549 tumor xenograft model. Consistent with the in vitro results, treatment with **L14e** significantly suppressed the growth of xenograft tumors with negligible changes in body weight (Fig. 7Ba). The therapeutic index of the PTX is 63.42 at 80 mg/kg dose, and the therapeutic index of the **L14e** is as high as 84.86 at the 80 mg/kg dose. Immunohistochemistry analysis results showed the expression of Ki67(a key indicator to measure the extent of malignancy) in tumors was significantly decreased by treatment with **L14e** (Fig. 7Bb). Simultaneously, the expression of cleaved-caspase-3 in tumors was significantly increased.

These results indicated that the **L14e** is the low injury for healthy tissues and effective in inhibiting cancer proliferation in vivo. And the result is significantly different (*P* < 0.001).

## Discussion

Tumor proliferation and migration are important causes of cancer progression. TS inhibitors can effectively inhibit tumor cell proliferation, but the clinically applied TS inhibitors have not inhibited tumor migration. Therefore, the development of TS inhibitors with pleiotropic inhibition is crucial. In this study, we first synthesized a total of 18 target compounds. In vitro enzyme activity assay results showed that target compounds could effectively inhibit TS enzyme, BRaf kinase, and EGFR kinase activity. And most of the compounds had excellent ability to inhibit cell viability against six cancer cell lines. Notably, the compound **L14e** had the superior anti-proliferative ability compared with PTX. This result demonstrated that the structural modification of the lead compound greatly enhances the inhibitory effect of the target compound on the proliferation of cancer cells.

Further mechanism studies showed that the compound **L14e** inhibited the proliferation of A549 and H460 cells by arresting cells in G1/S phase, then activating the P53-mitochondrial apoptotic pathway. Simultaneously, the results of the inhibition of transwell and tube formation proved that **L14e** could inhibit the activation of the EGFR signaling pathway and ultimately achieve the purpose of inhibiting cancer cell migration and angiogenesis. These results suggested that the compound L14e has multiple effects of inhibiting proliferation and migration of cancer cells. Besides, in vivo studies had shown **L14e** significantly inhibited tumor growth in xenograft tumors, the therapeutic index of the **L14e** was as high as 84.86 at 80 mg/kg dose, which was much higher than the therapeutic index of PTX. And it had a low injury to healthy tissues with minimal toxicity in vivo. These results indicated that **L14e** is safer in treatment than PTX.

All in vivo and in vitro results demonstrated that compound **L14e** has multiple effects of inhibiting tumor proliferation and migration, and has excellent value for further research and development. At the same time, the discovery of multiplexed TS inhibitors has brought good news for the clinical treatment of cancer, especially non-small cell lung cancer.

### Experimental section

#### General procedures

^1^H-NMR and ^13^C-NMR spectra were recorded on a Varian NMR spectrometers operating at 600 MHz for ^1^H, and 150 MHz for ^13^C. All chemical shifts were measured in DMSO-*d*_6_ as solvents. All chemicals were purchased from Sinoreagent Chemical Reagent (Beijing, China) and were used as received, unless stated otherwise. Analytical TLC is performed on silica gel 60 F254 plates (Qingdao Haiyang Chemical Company) and visualized by UV and potassium permanganate staining. Flash column chromatography is performed on gel 60 (40–63 mm) (Qingdao Haiyang Chemical Company). Melting points were determined with an Electro thermal melting point apparatus, are uncorrected. The results are loaded in the supplemental file.

### HPLC analysis of stability of compound in cell culture medium

The HPLC system consisted of e2695 Separations Module pump coupled to a 2489 UV/Visible detector (Waters Corp., Milford, MA, USA). Chromatograms was achieved on a Waters BDS C_18_ column (200 mm × 4.6 mm, 5 um). Detection was performed at a wavelength of 260 nm at 37 °C. The mobile phase consisted of 0.1% phosphoric acid water /methanol (50/50, v/v) at a flow rate of 1.0 mL/min. A10 µL of sample was injected. The results are loaded in the supplemental file.

### Cell culture

The human non-small cell lung cancer cell line A549 and H460, ovarian cancer cell line OVCAR-3, gastric carcinoma cell line SGC7901, the human colon cancer cell line HCT-116, the human liver cancer cell line hepG2 and the human breast cancer cell line MBA-MD-231 were purchased from the American Type Culture Collection (ATCC, Manassas, VA, USA). The cell lines were cultured according to the suppliers’ instructions. The cells were periodically authenticated by morphologic inspection and tested for mycoplasma contamination.

### Thymidylate synthase (TS) assays

Recombinant human thymidylate synthase purchased from ProSpec-Tany Company. TS was assayed spectrophotometrically at 30 °C and pH 7.4 in a mixture containing 0.1 M 2-mercaptoethanol, 0.0003 M (6*R*,*S*)-tetrahydrofolate, 0.012 M formaldehyde, 0.02 M MgCl_2_, 0.001 M dUMP, 0.04 M Tris–HCl, 30 nM hTS, and 0.00075 M NaEDTA. This was the assay described by Wahba and Friedkin^30^, except that the dUMP concentration was increased 25-fold according to the method of Davisson et al.^31^. The reaction was initiated by the addition of an amount of enzyme yielding a change in absorbance at 340 nm of 0.016/min in the absence of inhibitor.

### BRaf and EGFR kinase asays

The ADP-Glo™ kinase assay (Promega, Madison, WI) was used to screen target compounds for their inhibition effects for BRaf, EGFR kinase. The kinase assay was carried out in a 96-well plate, in a volume of 25 μL HEPES (pH 7.3) solvent containing 1.6 μg/mL EGF, and 20 ng of EGFR kinase, or 50 ng BRaf kinase, and 10 μM ATP (Promega, Madison, WI). The compound (**L13d-L13i**, **L14d-L14i**, and **L15d-L15i**) were dissolved in 100% DMSO, added to the system to give a final DMSO concentration of 1.0%. Reactions in each well were started immediately by adding ATP and kept going for half an hour under 30 °C in a constant temperature incubator (Bluepard, Shanghai, China). After the plate cooled for 5 min at room temperature, 25 μl of ADP-Glo reagent was added into each well to stop the reaction and consume the remaining ADP within 40 min. At the end, 50 μl of kinase detection reagent was added into the well and incubated for 1 h to produce a luminescence signal.

### MTT assays

Details are given in Supplementary Materials and Methods. Using probability unit and weighted regression method to calculate the IC_50_ value of compounds.

### Annexin V/propidium iodide (PI) staining

The density of the A549 cells and H460 in the logarithmic growth phase was adjusted to 1 × 10^6^ cells/mL, and a total of 1 mL of cell suspension and 1 mL of **L14e** at different concentrations were added into each well with the final compound **L14e** concentration in each group as 0, 0.5, 1.0, and 1.5 μM. Details are given in Supplementary Materials and Methods.

### Western blot analysis

A549 cells, H460 cells and HUVECs were treated with different concentrations of compound **L14e** for 24 h. Primary antibodies (100 μL/cm^2^): P53(10442-1-AP, Proteintech Group),caspase-3 (19677-1-AP, Proteintech Group), bax (50599-2-lg, Proteintech Group), bcl-2 (12789-1-AP, Proteintech Group), cleaved caspase-3 (25546-1-AP, Proteintech Group), β-actin (60008-1-lg, Proteintech Group), EGFR (ab21074, Abcam), AKT (#2920, Cell Signaling Technology), ERK1/2 (#4370, Cell Signaling Technology), VEGFR-2 (#9698, Cell Signaling Technology), p-EGFR (ab40815, Abcam), p-AKT (#4060, Cell Signaling Technology), p-ERK1/2 (#4695, Cell Signaling Technology), p-VEGFR-2 (#3770, Cell Signaling Technology), and GAPDH (ab181603, Abcam). The cellular levels of proteins were determined by standard Western blotting. Details were given in Supplementary Materials and Methods.

### Cell migration and wound healing assays

Cell migration ability was measured using transwell chambers (8-μm pore size; Corning Costar, Cambridge, MA, USA). For the transwell assay, A549 and H460 cells suspended in serum-free RPMI-1640 medium containing different concentrations of **L14e** (0, 0.5, 1.0, and 1.5 μM) were seeded into the upper chamber. The lower chamber contained RPMI-1640 medium supplemented with 20% serum. After 24 h incubation, the filters were fixed in methanol and stained with 0.1% crystal violet. The upper faces of the filters were gently abraded, and the lower faces with cells migrated across the filters were imaged and counted under the microscope. For wound healing assays, cells were placed into 6-well plates and cultured until 100% confluence. An artificial scratch was created using a 200 μL pipette tip. Add serum-free RPMI-1640 medium containing different concentrations of drugs. At 24 h after culturing in serum-free medium, wound closure images were captured in the same field under magnification. Cell healing rates were calculated by the fraction of cell coverage across the line. These experiments were performed in triplicate and repeated three times.

### Tube formation in vitro

Matrigel was thawed at 4 °C and 50 μL of the solution were added to each well in a 96-well plate and formed a gel at 37 °C for 30 min. HUVECs were suspended at 1 × 10^4^ cells in 100 μL of A549 cell supernatant with or without **L14e** (0, 0.5, 1.0, and 1.5 μM), and then added to each well. After 24h-incubation, the degree of tube formation was determined by counting the number of areas surrounded by tubes contained in 10 random fields, and expressed as mean ± SD. Using Image J for data analysis.

#### Toxicity test

FVB mice were treated with vehicle, **L14e** (40, 80, and 160 mg/kg), or PTX (80 mg/kg) every day for 3 weeks. Blood was collected from euthanized mice under isoflurane-induced deep anesthesia by cardiac puncture. After allowing blood coagulation at 4 °C, serum was collected by centrifugation at 3000 rpm for 10 min at 4 °C. Analysis of the level of ALT, AST, Glu and CRE in the serum was performed using a veterinary hematology analyzer (Fuji DRI-Chem 3500 s, Fujifilm, Tokyo, Japan) according to the manufacturer’s provided protocols. The histopathological changes in liver, lung, brain, and kidney were evaluated by using HE-stained section of the tissues (This part of the results is in the supplementary document).

### Tumor xenograft model

Five-week-old immunodeficient BABL/c female nude mice, weighing 18 to 20 g, were purchased and maintained under specific pathogen-free conditions. They were implanted subcutaneously with 1 × 10^7^ A549 cells. Tumor sizes were assessed using the two largest perpendicular axes. Tumor volume was calculated using the formula **V** **=** **(a** **×** **b**^**2**^**)/2**, where **a** is length and **b** is width. The tumor volume was measured every 3 days. When tumor volumes reached 50 mm^3^, the mice were randomized to drug-treated or vehicle groups (five mice per group). Tumor growth was monitored every 3 days using a Vernier caliper. For a total of 3 weeks, the **L14e** (80 mg/kg) and PTX (80 mg/kg) was intraperitoneal injection, after which the mice were euthanized and the xenograft tumors were dissected. All animal studies were conducted with the approval of the Laboratory Animal Welfare and Ethical Committee of China Medical University.

### HE staining

Tissues were fixed in 10% neutral-buffered formalin for 24 h, embedded in paraffin, cut into 4-μm-thick sections, deparaffinized with xylene and processed with a graded ethanol series. Next, the sections were stained with HE and were observed using Eclipse Ts2 microscope (Nikon).

#### Immunohistochemistry

Sections derived from formalin-fixed and paraffin-embedded murine lung tissues were deparaffinized by incubation overnight at 65 °C followed by rehydration in sequential xylene and ethanol rinses. After incubation with hydrogen peroxide, the slides were washed with PBS and then incubated with 0.4% Triton X-100. The sections were incubated with blocking solution (Dako Protein Block, Dako, Glostrup, Denmark) for 30 min at room temperature after washing with PBS. The sections were further incubated with primary antibodies (Ki67 and cleaved caspase 3 [all from Cell Signaling Technology], diluted at 1:200) overnight at 4 °C, washed with PBS several times, incubated with the corresponding biotinylated secondary antibodies (diluted at 1:500), and then washed with PBS multiple times. After adding avidin-biotin complexes (Vector Laboratories), the sections were visualized using diaminobenzidine (DAB) detection reagent (Enzo Life Sciences, Farmingdale, NY, USA) and mounted with a mounting solution (Vector Laboratories, Burlingame, CA, USA).

### Statistical analysis

Data were analyzed using GraphPad Prism 5 (GraphPad Software; La Jolla, CA). Normal distributed measurement for two groups was conducted using the *t*-test, and comparisons among multiple groups were determined using one-way analysis of variance (ANOVA). Numeric data were presented as either ratios or percentages. *P* *<* 0.05 was considered to be statistically significant.

## Supplementary information

Discovery of N-phenyl-(2,4-dihydroxypyrimidine-5-sulfonamido) phenylurea-based thymidylate synthase (TS) inhibitor as a novel multi-effects antitumor drugs with minimal toxicity
