# Supplementary material for: Discovery of N-phenyl-(2,4-dihydroxypyrimidine-5-sulfonamido) phenylurea-based thymidylate synthase (TS) inhibitor as a novel multi-effects antitumor drugs with minimal toxicity
Source: Cell Death Dis. 2019 Jul 11;10(7):532. doi: 10.1038/s41419-019-1773-0 (PMC6624297; doi:10.1038/s41419-019-1773-0)
Supplement: Supplementary file 1 — Discovery of N-phenyl-(2,4-dihydroxypyrimidine-5-sulfonamido) phenylurea-based thymidylate synthase (TS) inhibitor as a novel multi-effects antitumor drugs with minimal toxicity [file 41419_2019_1773_MOESM1_ESM.doc]

# Supplementary Data

# Discovery of N-phenyl-(2,4-dihydroxypyrimidine-5-sulfonamido) phenylurea-based thymidylate synthase (TS) inhibitor as a novel multi-effects antitumor drugs with minimal toxicity

Xin-yang Li^1,#^, Ting-jian Zhang^2,#^, Mohamed Olounfeh Kamara ^3,#^,Guo-qing Lu^4,#^, Haili Xu^5,#^, Depu Wang^6,#^, Fan-hao Meng*^,#^

^#^School of Pharmacy, China Medical University, 77 Puhe Road, Shenyang 110122, China

^1^ E-mail: xinyanglicmu@163.com

^2^ E-mail: todayfy@outlook.com

^3^ E-mail: mohamedkamara6994@yahoo.com

^4^ E-mail: luxiaoqing125@163.com

^5^ E-mail:17309813989@163.com

^6^ E-mail: www22229988@163.com

*Correspondence: Professor Fan-hao Meng, School of pharmacy, China Medical University, Shenyang 110122, P. R. China.

E-mail: fhmeng@cmu.edu.cn

Fax: +86-24-31939448

**Supplementary experimental method**

**The synthesis of 2, 4-dihydroxypyrimidine-5-sulfonylchloride (L1).**

Preparation of 2, 4-dihydroxypyrimidine-5-sulfonylchloride was done according to the reported method in our past work.

**General procedure for the synthesis of compounds L3a-L3c.**

To a solution of corresponding methyl nitrobenzoate (10 g, 55.22 mmol) in methanol (80 mL), hydrazine monohydrate (80%) (13.82 g, 22082 mmol) was added. The reaction mixture was heated under reflux for 6-8 h. After completion of the reaction, the solvent was evaporated under reduced pressure and the residue was washed with water, and the obtained solid was filtered and dried to give the compound **L3a-L3c**.

4-nitro benzohydrazide **(L3a):** a light yellow solid, yield: 88.62%. 2-nitro benzohydrazide**(L3b):** a white solid, yield: 78.45%. 3-nitro benzohydrazide **(L3c):** a yellow solid, yield: 83.26%.

**General procedure for the synthesis of compounds L4a-L4c.**

To a stirred solution of corresponding nitrobenzohydrazide(L3a-L3c) (3 g, 16.56 mmol) in DCM, the solution of NaNO_2_ (1.37 g, 19.87 mmol) in water (25 mL) was added, then the solution of 37% HCl (2.12 g, 21.53 mmol）in water (20 mL) was added dropwise in ice cooled bath. The reaction mixture was stirred at 0℃ for 1.5 h and the reaction was monitored by TLC. After completion of the reaction, the organic phase was separated and dried over anhydrous Na_2_SO_4_, the solvent was removed in vacuo, and the residue was purified by silica gel column to give the pure compound L4a-L4c.4-nitrobenzoyl azide (L4a): a light yellow solid, yield: 76.89%.2-nitrobenzoyl azide (L4b): a light yellow solid, yield: 72.33 %.3-nitrobenzoyl azide (L4c): a light yellow solid, yield: 80.03 %.

**General procedure for the synthesis of compounds L7d-l7i.**

The solution of the compound **L4a** (2.5 g, 13.01 mmol) in 1, 2-dichloroethane (20mL) was stirred at 80°C for 1.5h to obtain the compound **L5a**. Within the same reaction system, then **L6d-L6i** (13.01 mmol) was added drop wise, the obtained solid was filtered, washed with petroleum ether and dried to give the compound **L7d–L7i**.

1-(2-chlorophenyl)-3-(4-nitrophenyl)urea (**L7d**): a light yellow solid, yield: 91.32 %.

1-(3-chlorophenyl)-3-(4-nitrophenyl)urea (**L7e**): a yellow solid, yield: 89.68 %.

1-(4-chlorophenyl)-3-(4-nitrophenyl)urea (**L7f**): a light yellow solid, yield: 92.75 %.

1-(4-nitrophenyl)-3-(*o*-tolyl)urea (**L7g**): a yellow solid, yield: 87.65 %.

1-(4-nitrophenyl)-3-(*m*-tolyl)urea (**L7h**): a light yellow solid, yield: 86.94 %.

1-(4-nitrophenyl)-3-(*p*-tolyl)urea (**L7i**): a light yellow solid, yield: 88.16 %.

**General procedure for the synthesis of compounds L10d-L10i.**

To a solution of compound **L7d-l7i** (8.57 mmol) in methanol (60 mL), the solution of NH_4_Cl (4.58 g, 85.71 mmol) in water (20 mL) and zinc dust (3.36 g, 51.42 mmol) were added and the mixture was stirred at room temperature for 6-8 h and the reaction was monitored by TLC. After completion of the reaction, the filtrate was obtained by suction filtered. The solvent was evaporated under reduce pressure, and then the water (150 mL) was added, the obtained solid was filtered and dried to give the compound **L10d-L10i**.

1-(4-aminophenyl)-3-(2-chlorophenyl)urea (**L10d**): a light yellow solid, yield: 78.45%.

1-(4-aminophenyl)-3-(3-chlorophenyl)urea **(L10e)**: a light yellow solid, yield: 82.95%.

1-(4-aminophenyl)-3-(4-chlorophenyl)urea (**L10f**): a light yellow solid, yield: 86.47%.

1-(4-aminophenyl)-3-(*o*-tolyl)urea (**L10g**): a light yellow solid, yield: 75.82%.

1-(4-aminophenyl)-3-(*m*-tolyl)urea (**L10h**): a light yellow solid, yield: 77.64 %.

1-(4-aminophenyl)-3-(*p*-tolyl)urea (**L10i**): a light yellow solid, yield: 85.42 %.

**General procedure for the synthesis of compounds L8d-l8i.**

The solution of the compound **L4b** (2.5 g, 13.01 mmol) in 1,2-dichloroethane (20 mL) was stirred at 80°C for 1.5h to obtain the compound **L5b**, then **L6d-L6i** (13.01 mmol) was added dropwise, the obtained solid was filtered, washed with petroleum ether and dried to give the compound **L8d** –**L8i**.

1-(2-chlorophenyl)-3-(2-nitrophenyl)urea (**L8d**): a yellow solid, yield: 85.42 %.

1-(3-chlorophenyl)-3-(2-nitrophenyl)urea (**L8e**): a light yellow solid, yield: 88.57 %.

1-(4-chlorophenyl)-3-(2-nitrophenyl)urea (**L8f**): a yellow solid, yield: 90.15 %.

1-(2-nitrophenyl)-3-(*o*-tolyl)urea (**L8g**): a light yellow solid, yield: 93.48 %.

1-(2-nitrophenyl)-3-(*m*-tolyl)urea (**L8h**): a light yellow solid, yield: 89.17%.

1-(2-nitrophenyl)-3-(*p*-tolyl)urea (**L8i**): a light yellow solid, yield: 94.65 %.

**General procedure for the synthesis of compounds L11d-L11i.**

To a solution of compound **L8d-L8i** (9.22 mmol) in methanol (60 mL), the solution of NH_4_Cl (4.93 g, 92.16mmol) in water (20 mL) and zinc dust (3.62 g, 55.29 mmol) were added and the mixture was stirred at room temperature for 6-8 h and the reaction was monitored by TLC. After completion of the reaction, the filtrate was obtained by suction filtered. The solvent was evaporated under reduce pressure, and then the water (150 mL) was added, the obtained solid was filtered and dried to give the compound **L11d-L11i**.

1-(2-aminophenyl)-3-(2-chlorophenyl)urea (**L11d**): a light yellow solid, yield: 88.52 %.

1-(2-aminophenyl)-3-(3-chlorophenyl)urea (**L11e**): a light yellow solid, yield: 84.26 %.

1-(2-aminophenyl)-3-(4-chlorophenyl)urea (**L11f**): a light yellow solid, yield: 79.45 %.

1-(2-aminophenyl)-3-(*o*-tolyl)urea (**L11g**): a light yellow solid, yield: 82.38 %.

1-(2-aminophenyl)-3-(*m*-tolyl)urea (**L11h**): a light yellow solid, yield: 86.15 %.

1-(2-aminophenyl)-3-(*p*-tolyl)urea (**L11i**): a light yellow solid, yield: 79.86 %.

**General procedure for the synthesis of compounds L9d-L9i.**

The solution of the compound **L4c** (2.5 g, 13.01mmol) in 1,2-dichloroethane (20 mL) was stirred at 80°C for 1.5h to obtain the compound **L5c**, then **L6d-L6i** (13.01 mmol) was added dropwise, the obtained solid was filtered, washed with petroleum ether and dried to give the compound **L9d** –**L9i**.

1-(2-chlorophenyl)-3-(3-nitrophenyl)urea (**L9d**): a light yellow solid, yield: 77.90 %.

1-(3-chlorophenyl)-3-(3-nitrophenyl)urea (**L9d**): a light yellow solid, yield: 80.74 %.

1-(4-chlorophenyl)-3-(3-nitrophenyl) urea (**L9d**): a light yellow solid, yield: 78.75 %.

1-(3-nitrophenyl)-3-(*o*-tolyl)urea (**L9d**): a light yellow solid, yield: 76.20%.

1-(3-nitrophenyl)-3-(*m*-tolyl)urea (**L9d**): a light yellow solid, yield: 79.89 %.

1-(3-nitrophenyl)-3-(*p*-tolyl)urea (**L9d**): a light yellow solid, yield: 81.47 %.

**General procedure for the synthesis of compounds L12d-L12i.**

To a solution of compound **L9d-l9i** (8.57 mmol) in methanol (60 mL), the solution of NH_4_Cl (4.58 g, 85.71 mmol) in water (20 mL) and zinc dust (3.36 g, 51.42 mmol) were added and the mixture was stirred at room temperature for 6-8 h and the reaction was monitored by TLC. After completion of the reaction, the filtrate was obtained by suction filtered. The solvent was evaporated under reduce pressure, and then the water (150 mL) was added, the obtained solid was filtered and dried to give the compound **L12d-L12i**.

1-(3-aminophenyl)-3-(2-chlorophenyl)urea (**L12d**): a light yellow solid, yield: 84.23 %.

1-(3-aminophenyl)-3-(3-chlorophenyl)urea (**L12e**): a light yellow solid, yield: 87.84%.

1-(3-aminophenyl)-3-(4-chlorophenyl)urea (**L12f**): a light yellow solid, yield: 79.28%.

1-(3-aminophenyl)-3-(*o*-tolyl)urea (**L12g**): a white solid, yield: 77.48 %.

1-(3-aminophenyl)-3-(*m*-tolyl)urea (**L12h**): a light yellow solid, yield: 83.33 %.

1-(3-aminophenyl)-3-(*p*-tolyl)urea (**L12i)**: a light yellow solid, yield: 81.08 %.

**Chemical synthesis**

**L13d**

*N*'-(2-chlorophenyl)-4-(2,4-dihydroxypyrimidine-5-sulfonamido)phenylurea.

A off white solid, yield: 85.79 %. Mp: 167-169°C. ^1^H NMR (600MHz, DMSO-*d_6_*) *δ* 11.87 (d, *J* = 5.1 Hz, 1 H), 11.63 (s, 1 H), 9.96 (br. s., 1 H), 9.84 (s, 1 H), 8.53 (s, 1 H), 8.11 (d, *J* = 8.1 Hz, 1 H), 7.96 (d, *J* = 6.1 Hz, 1 H), 7.48 - 7.34 (m, 3 H), 7.28 (t, *J* = 7.6 Hz, 1 H), 7.08 - 6.95 (m, 3 H); ^13^C NMR (150 MHz, DMSO-*d*_6_) *δ* 159.21, 152.78, 150.82, 148.43, 136.90, 136.54, 131.77, 129.65, 127.89, 123.68, 122.68, 122.24, 122.02, 119.13, 111.24. ESI-HRMS calcd for C_17_H_14_ClN_5_O_5_S. [M - H]^+^ 434.0382, found: 434.0311.


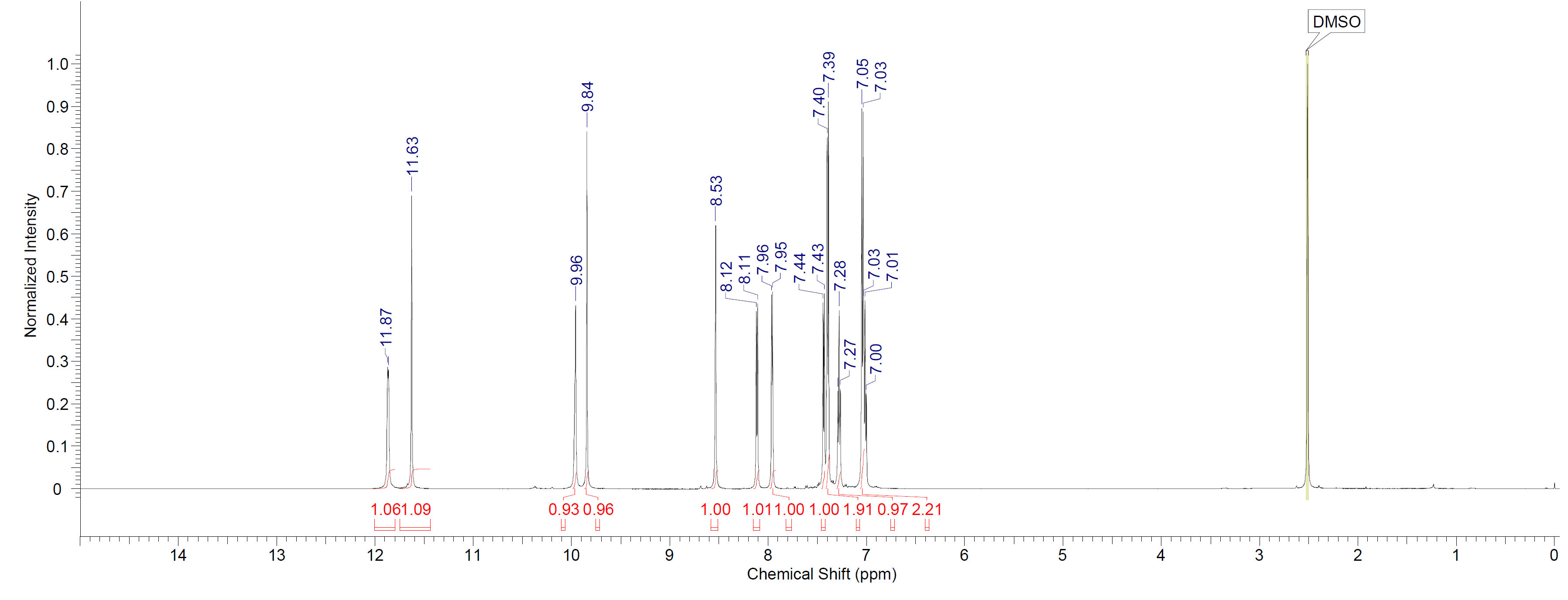

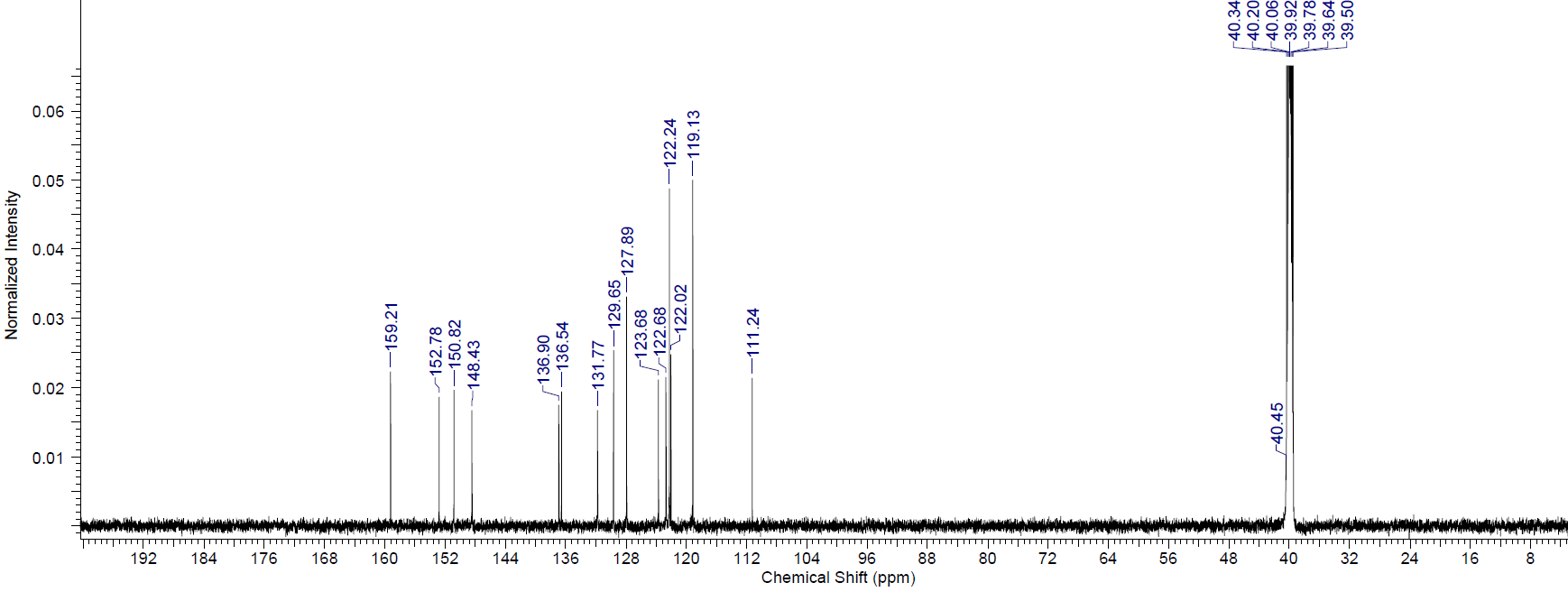


**L13e**

N'-(3-chlorophenyl)-4-(2,4-dihydroxypyrimidine-5-sulfonamido)phenylurea.

A white solid, yield: 85.00 %. Mp: 169-171°C. ^1^H NMR (600MHz, DMSO-*d_6_*) *δ* 11.76 (br. s., 1 H), 11.62 (s, 1 H), 9.84 (s, 1 H), 9.48 (br. s., 1 H), 9.22 (br. s., 1 H), 7.96 (s, 1 H), 7.69 (s, 1 H), 7.34 (d, *J* = 8.6 Hz, 2 H), 7.31 - 7.24 (m, 2 H), 7.03 (d, *J* = 8.6 Hz, 2 H), 7.00 (br. s., 2 H); ^13^C NMR (150 MHz, DMSO-*d*_6_) *δ* 159.19, 152.97, 150.87, 148.45, 141.92, 136.69, 133.62, 131.84, 130.85, 122.21, 121.64, 119.26, 117.63, 116.74, 111.27. ESI-HRMS calcd for C_17_H_14_ClN_5_O_5_S. [M - H]^+^ 434.0385, found: 434.0313.


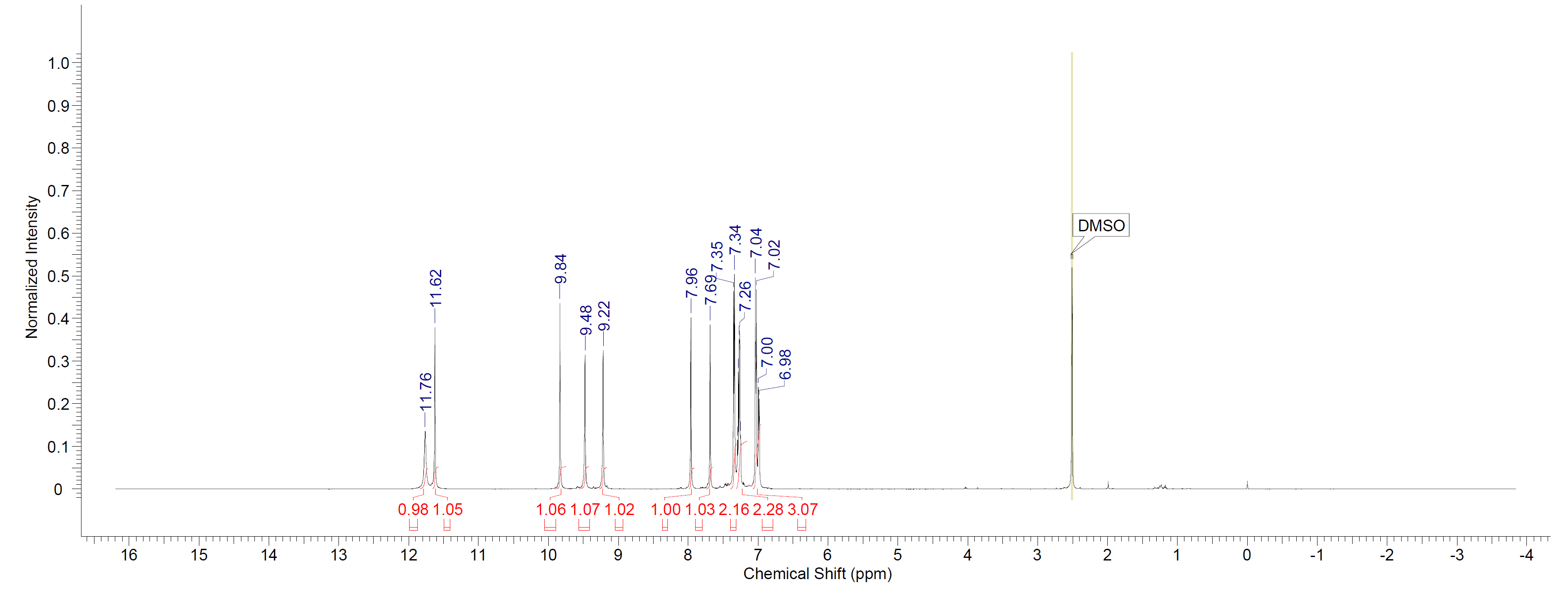

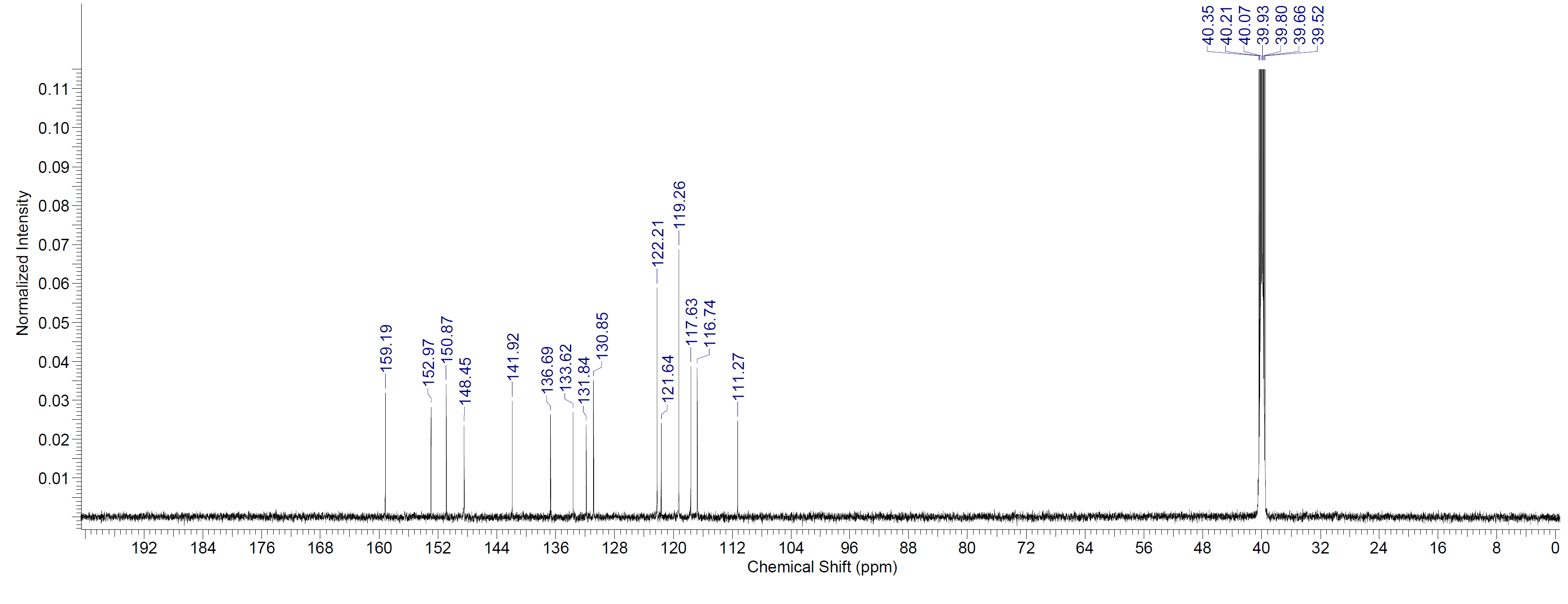


**L13f**

N'-(4-chlorophenyl)-4-(2,4-dihydroxypyrimidine-5-sulfonamido)phenylurea.

A off brown solid, yield: 89.15 %. Mp: 175-177°C. ^1^H NMR (600MHz, DMSO-*d_6_*) *δ* 11.86 (d, *J* = 5.5 Hz, 1 H), 11.63 (s, 1 H), 9.82 (s, 1 H), 9.73 (s, 1 H), 9.51 (s, 1 H), 7.96 (s, 1 H), 7.48 (d, *J* = 8.8 Hz, 2 H), 7.37 - 7.27 (m, 4 H), 7.03 (d, *J* = 8.8 Hz, 2 H); ^13^C NMR (150 MHz, DMSO-*d*_6_) *δ* 159.19, 153.17, 150.84, 148.37, 140.41, 137.18, 131.48, 122.37, 121.99, 122.03, 118.97, 118.31, 111.30. ESI-HRMS calcd for C_17_H_14_ClN_5_O_5_S. [M - H]^+^ 434.0397, found: 434.0325.


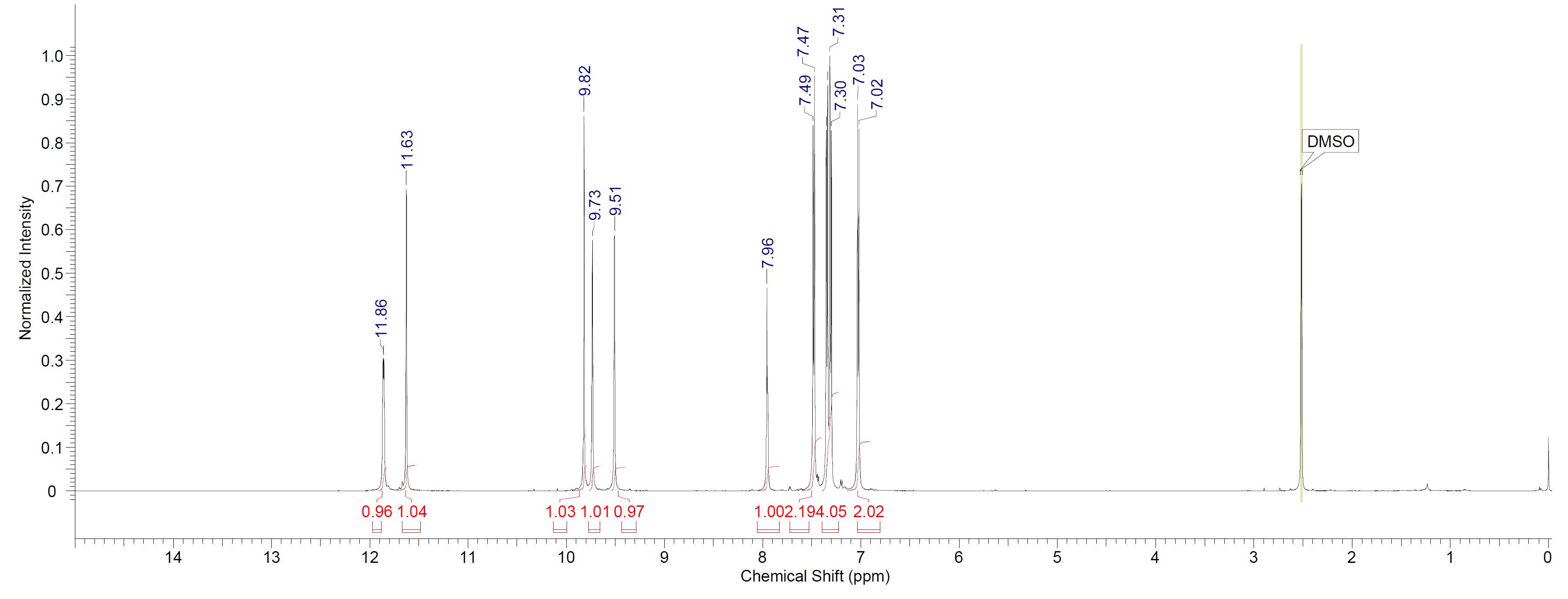

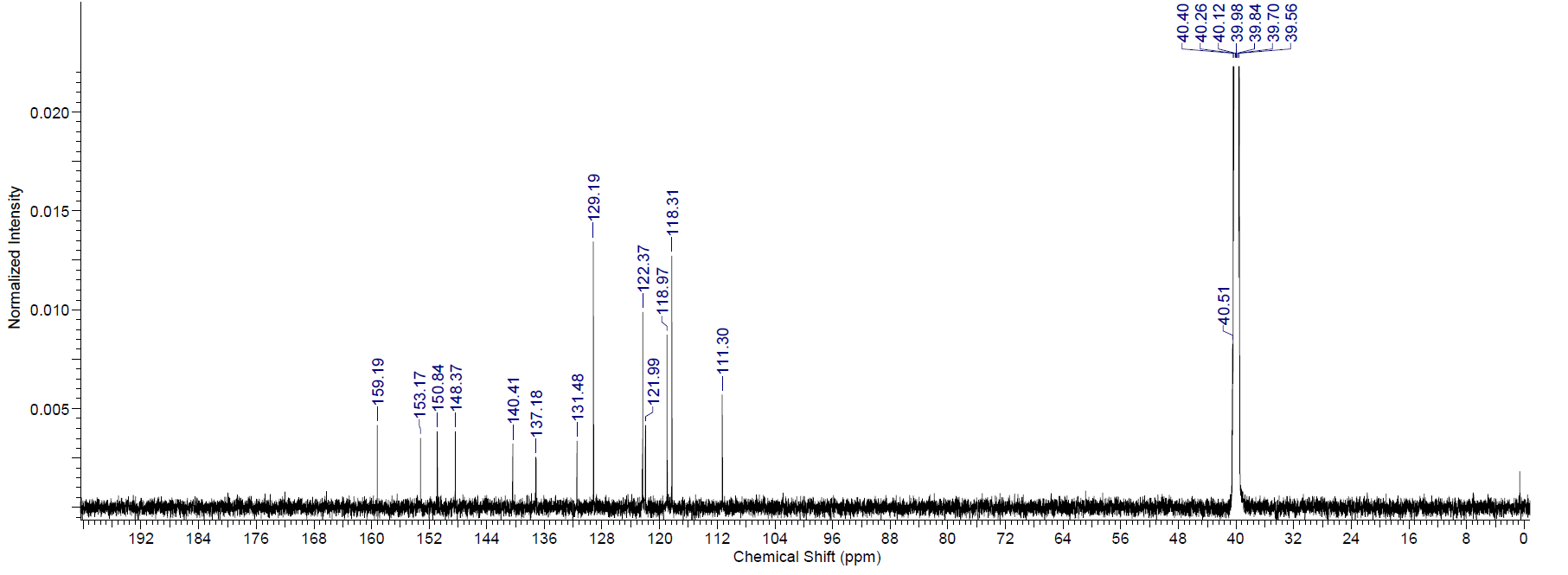


**L13g**

N'-(2-methylphenyl)-4-(2,4-dihydroxypyrimidine-5-sulfonamido)phenyl urea.

A white solid, yield: 80.10 %. Mp: 169-173°C. ^1^H NMR (600MHz, DMSO-d_6_) *δ* 11.74 (br. s., 1 H), 11.61 (s, 1 H), 9.78 (s, 1 H), 9.40 (br. s., 1 H), 8.16 (br. s., 1 H), 7.94 (d, *J* = 6.4 Hz, 1 H), 7.81 (d, *J* = 7.9 Hz, 1 H), 7.37 (d, *J* = 8.7 Hz, 2 H), 7.20 - 7.07 (m, 2 H), 7.02 (d, *J* = 8.8 Hz, 2 H), 6.96 - 6.86 (m, 1 H), 2.24 (s, 3 H); ^13^C NMR (150 MHz, DMSO-*d*_6_) *δ* 159.19, 153.30, 150.85, 148.38, 138.05, 137.45, 131.34, 130.58, 127.94, 126.48, 122.84, 122.41, 121.32, 118.81, 111.29, 18.72. ESI-HRMS calcd for C_18_H_17_lN_5_O_5_S. [M - H]^+^ 414.0942, found: 414.0870.


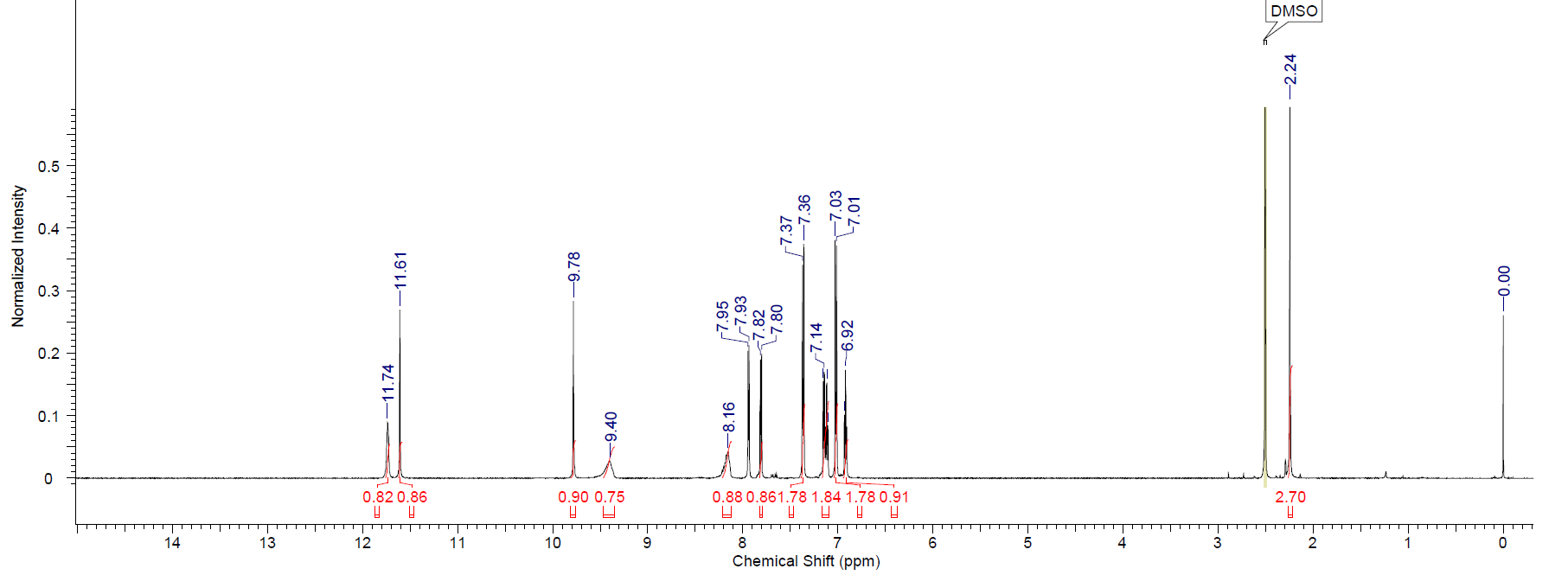

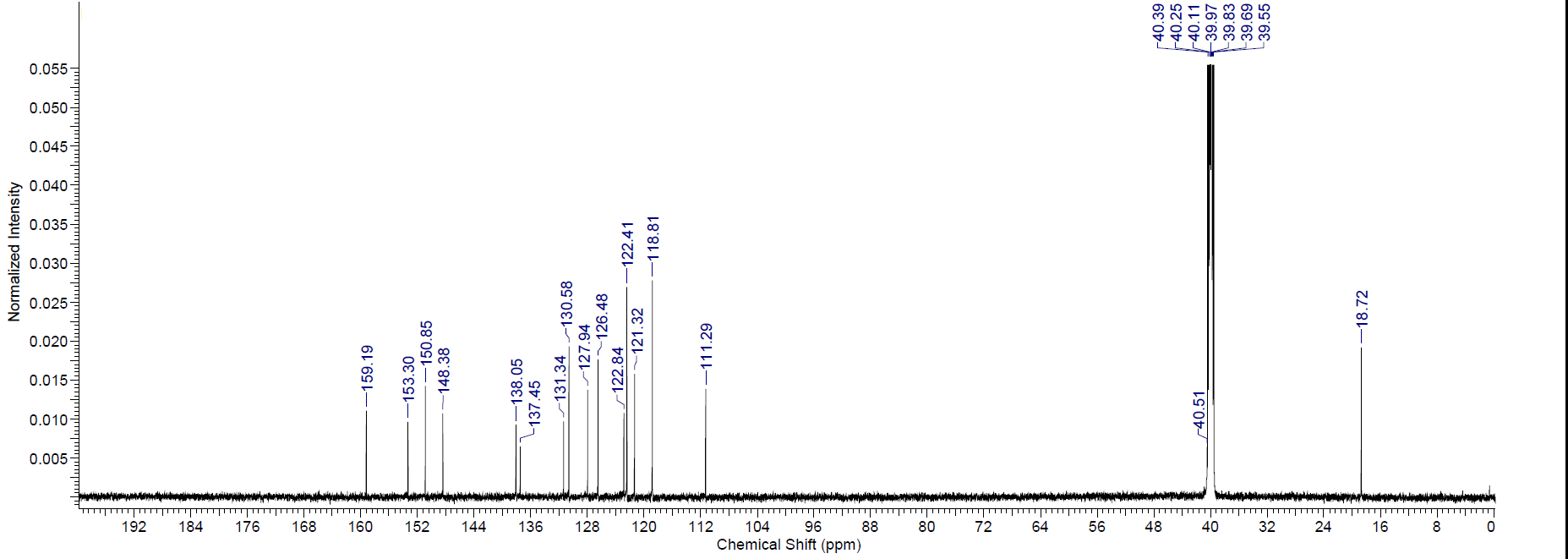


**L13h**

*N'*-(3-methylphenyl)-4-(2,4-dihydroxypyrimidine-5-sulfonamido)phenylurea.

A off brown solid, yield: 83.50 %. Mp: 179-182°C. ^1^H NMR (600MHz, DMSO-*d_6_*) *δ* 11.70 (br. s., 1 H), 11.62 (s, 1 H), 9.82 (s, 1 H), 8.68 (s, 1 H), 8.64 (s, 1 H), 7.96 (br. s., 1 H), 7.35 - 7.32 (m, 2 H), 7.27 (s, 1 H), 7.21 (d, *J* = 8.4 Hz, 1 H), 7.16 - 7.11 (m, 1 H), 7.05 - 7.00 (m, 2 H), 6.77 (d, *J* = 7.3 Hz, 1 H), 2.26 (s, 3 H); ^13^C NMR (150 MHz, DMSO-*d*_6_) *δ* 159.18, 152.97, 150.90, 148.43, 140.11, 138.38, 136.93, 131.65, 129.07, 122.94, 122.23, 119.22, 119.05, 115.73, 111.31, 21.70. ESI-HRMS calcd for C_18_H_17_lN_5_O_5_S. [M - H]^+^ 414.0958, found: 414.0886.


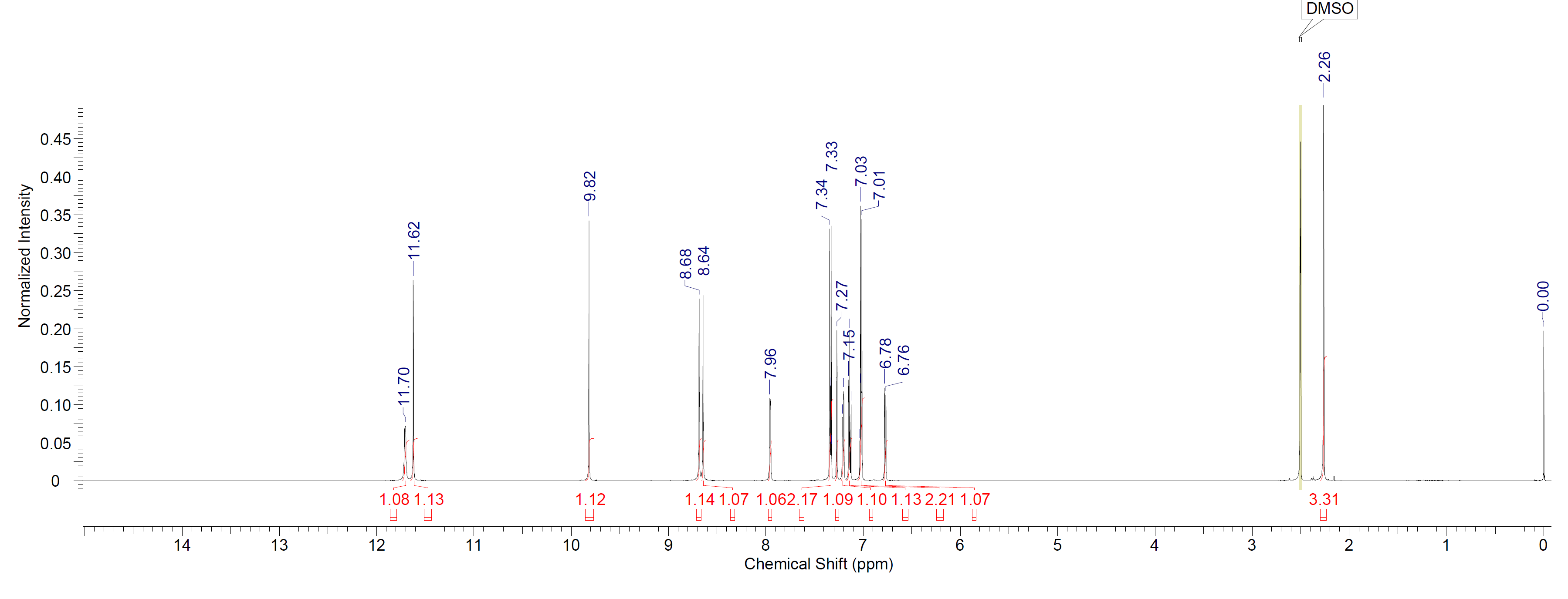

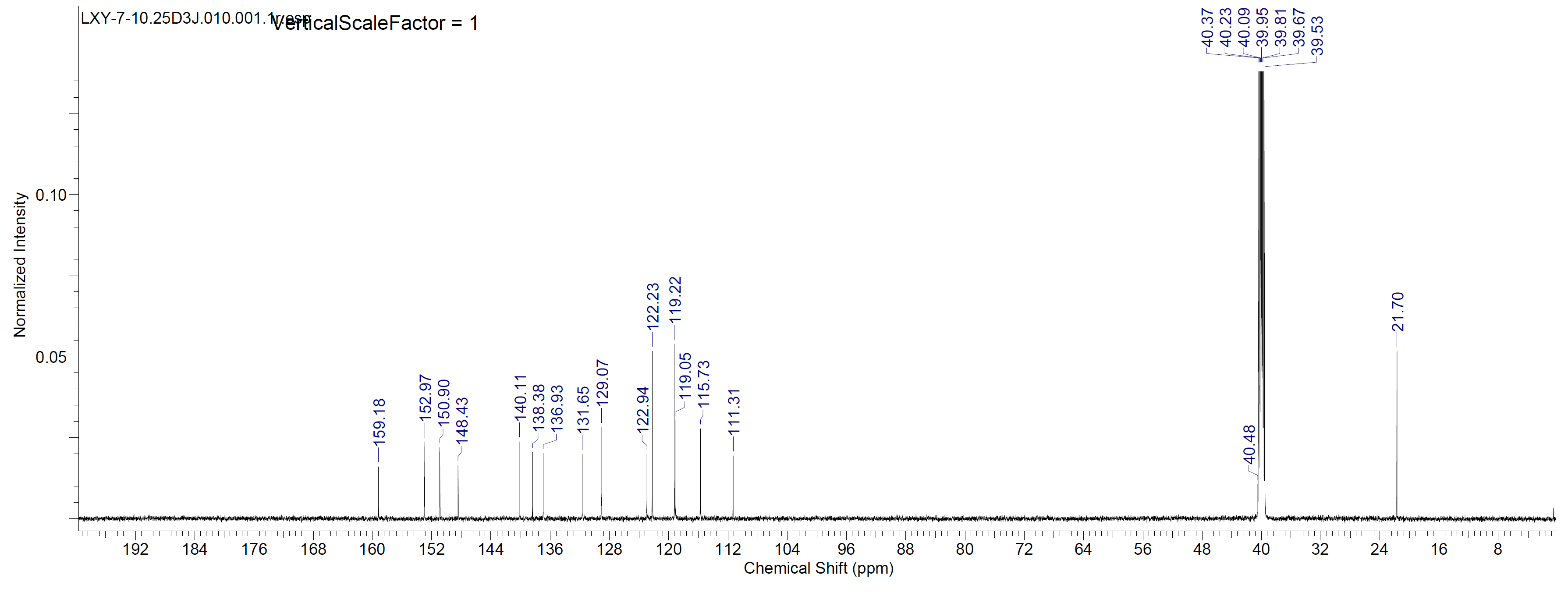


**L13i**

N'-(4-methylphenyl)-4-(2,4-dihydroxypyrimidine-5-sulfonamido) phenyl urea.

A off white solid, yield: 82.90 %. Mp: 181-183°C. ^1^H NMR (600MHz ,DMSO-*d_6_*) *δ* 11.71 (br. s., 1 H), 11.62 (s, 1 H), 9.81 (s, 1 H), 8.69 (s, 1 H), 8.65 (s, 1 H), 7.95 (s, 1 H), 7.32 (dd, *J* = 8.6, 11.7 Hz, 4 H), 7.07 (d, *J* = 8.3 Hz, 2 H), 7.02 (d, *J* = 8.8 Hz, 2 H), 2.23 (s, 3 H); ^13^C NMR (150 MHz, DMSO-*d*_6_) δ159.18, 153.04, 150.90, 148.42, 137.62, 137.02, 131.58, 129.62, 122.26, 119.19, 118.64, 111.31, 20.80. ESI-HRMS calcd for C_18_H_17_lN_5_O_5_S. [M - H]^+^ 414.0927, found: 414.0885.


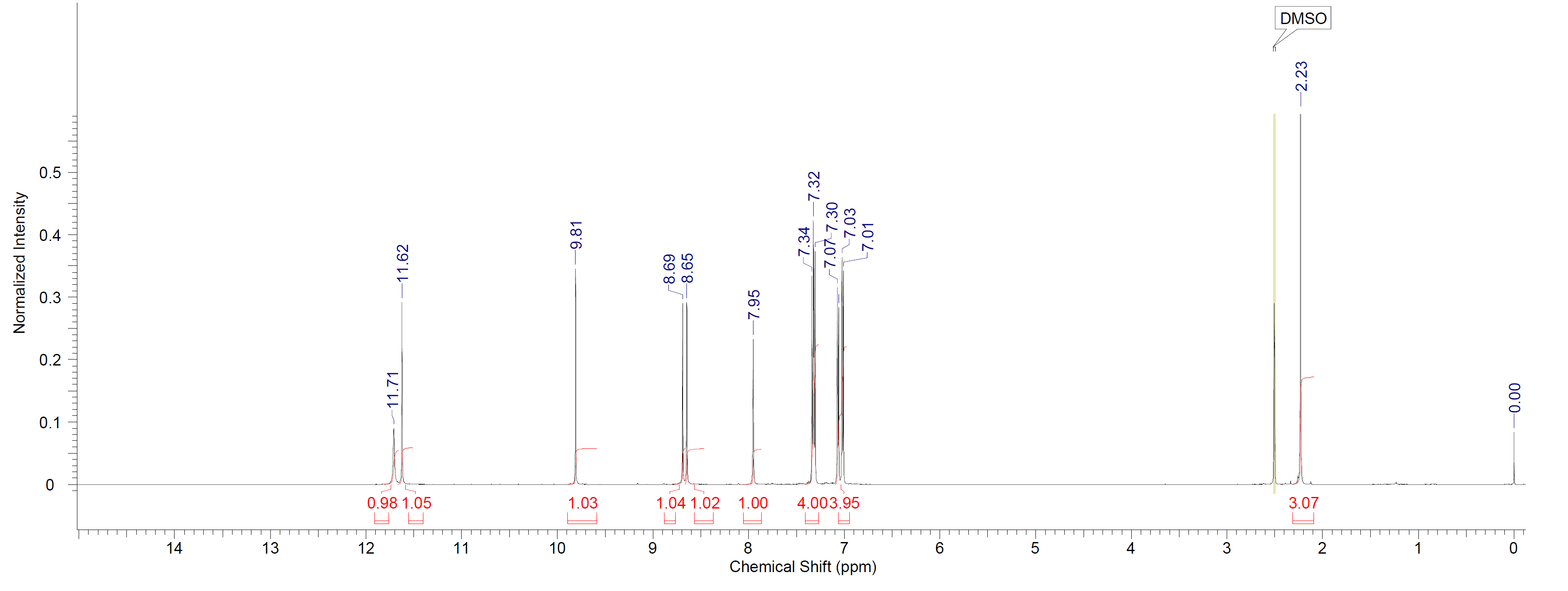

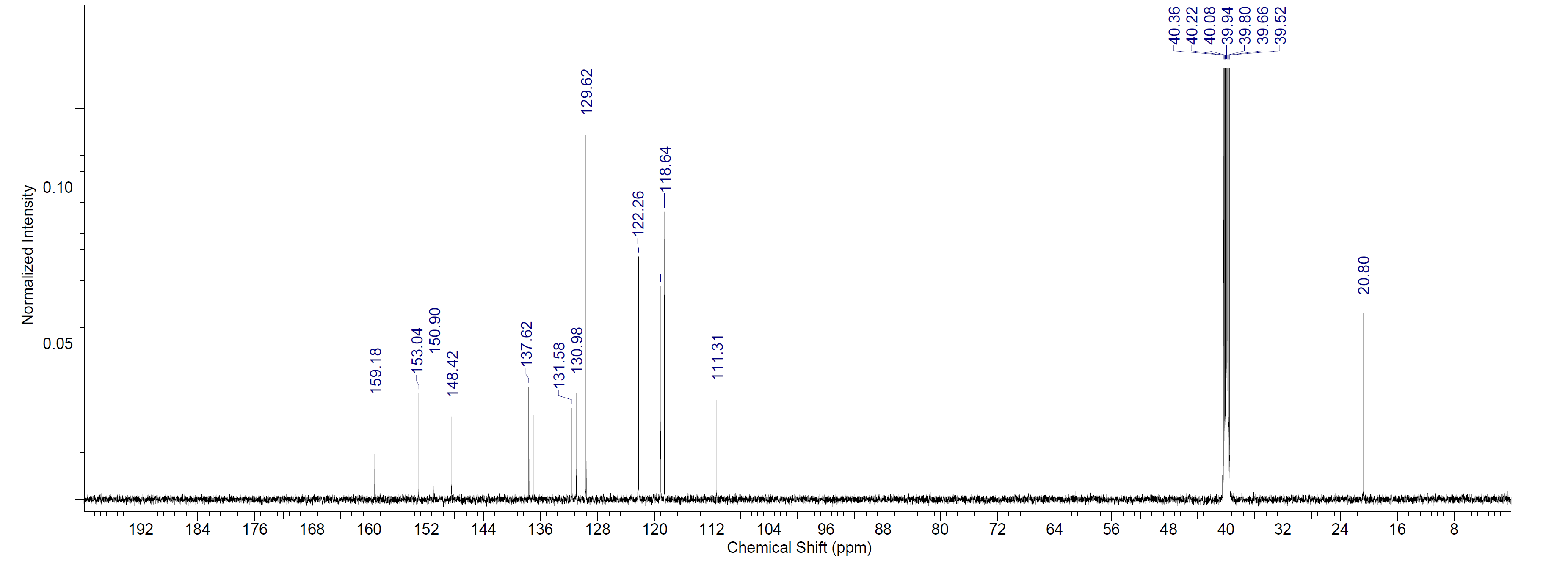


**L14d**

N'-(2-chlorophenyl)-2-(2,4-dihydroxypyrimidine-5-sulfonamido)phenyl urea.

A white solid, yield: 83.73 %. Mp: 151-153°C. ^1^H NMR (600MHz ,DMSO-*d_6_*) *δ* 11.69 (br. s., 1 H), 9.12 (br. s., 1 H), 8.84 (s, 1 H), 8.08 (s, 1 H), 7.88 (d, *J* = 8.1 Hz, 1 H), 7.82 (br. s., 1 H), 7.46 (d, *J* = 7.7 Hz, 1 H), 7.30 (t, *J* = 7.5 Hz, 1 H), 7.24 - 7.16 (m, 1 H), 7.08 - 7.02 (m, 2 H), 7.03 - 6.96 (m, 1 H); ^13^C NMR (150 MHz, DMSO-*d*_6_) *δ* 159.79, 153.33, 150.79, 148.01, 141.79, 136.27, 133.60, 130.85, 128.01, 127.97, 126.46, 123.55, 121.92, 121.89, 118.07, 117.12, 111.43. ESI-HRMS calcd for C_17_H_14_ClN_5_O_5_S. [M - H]^+^ 434.0385, found: 434.0313.


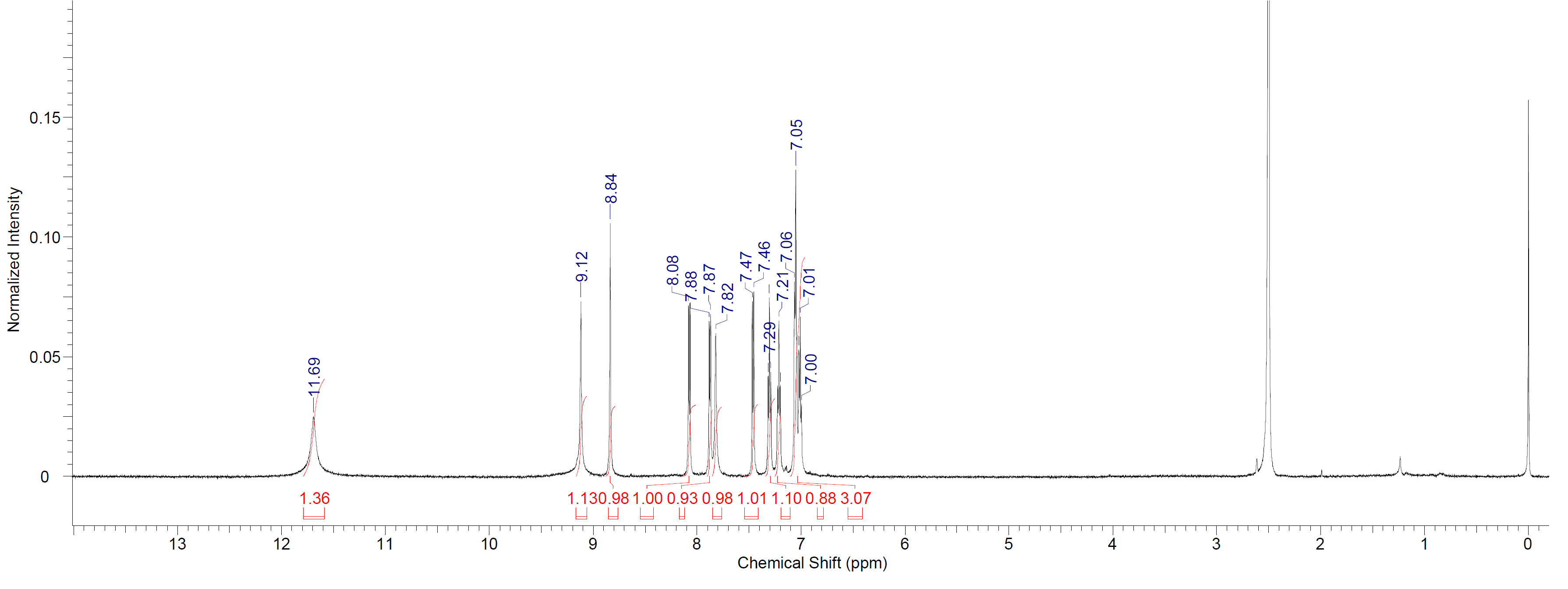

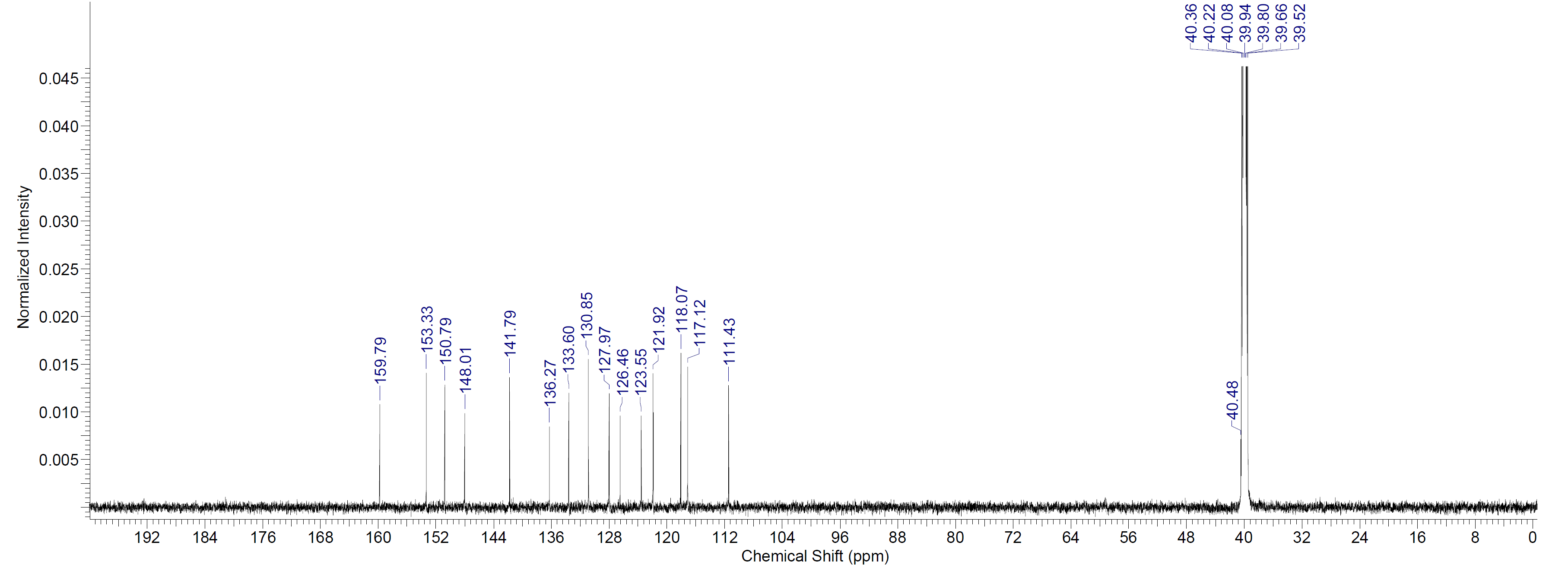


**L14e**

N'-(3-chlorophenyl)-2-(2,4-dihydroxypyrimidine-5-sulfonamido)phenyl urea.

A light brown solid, yield: 88.10 %. Mp: 159-161°C. ^1^H NMR (600MHz ,DMSO-*d_6_*) *δ* 11.71 (br. s., 1 H), 9.20(br. s., 1 H), 9.11 (br. s., 1 H), 8.84 (s, 1 H), 8.08 (s, 1 H), 7.87 (d, *J* = 8.1 Hz, 1 H), 7.82 (br. s., 1 H), 7.46 (d, *J* = 7.9 Hz, 1 H), 7.30 (t, *J* = 7.7 Hz, 1 H), 7.21 (t, *J* = 7.5 Hz, 1 H), 7.08 - 7.03 (m, 2 H), 7.03 - 6.97 (m, 1 H); ^13^C NMR (150 MHz, DMSO-*d*_6_) *δ* 160.00, 153.24, 151.19, 148.47, 136.60, 136.41, 129.69, 128.11, 128.01, 127.88, 126.42, 124.15, 123.44, 123.38, 123.09, 121.95, 111.31. ESI-HRMS calcd for C_17_H_14_ClN_5_O_5_S. [M - H]^+^ 434.0382, found: 434.0310.


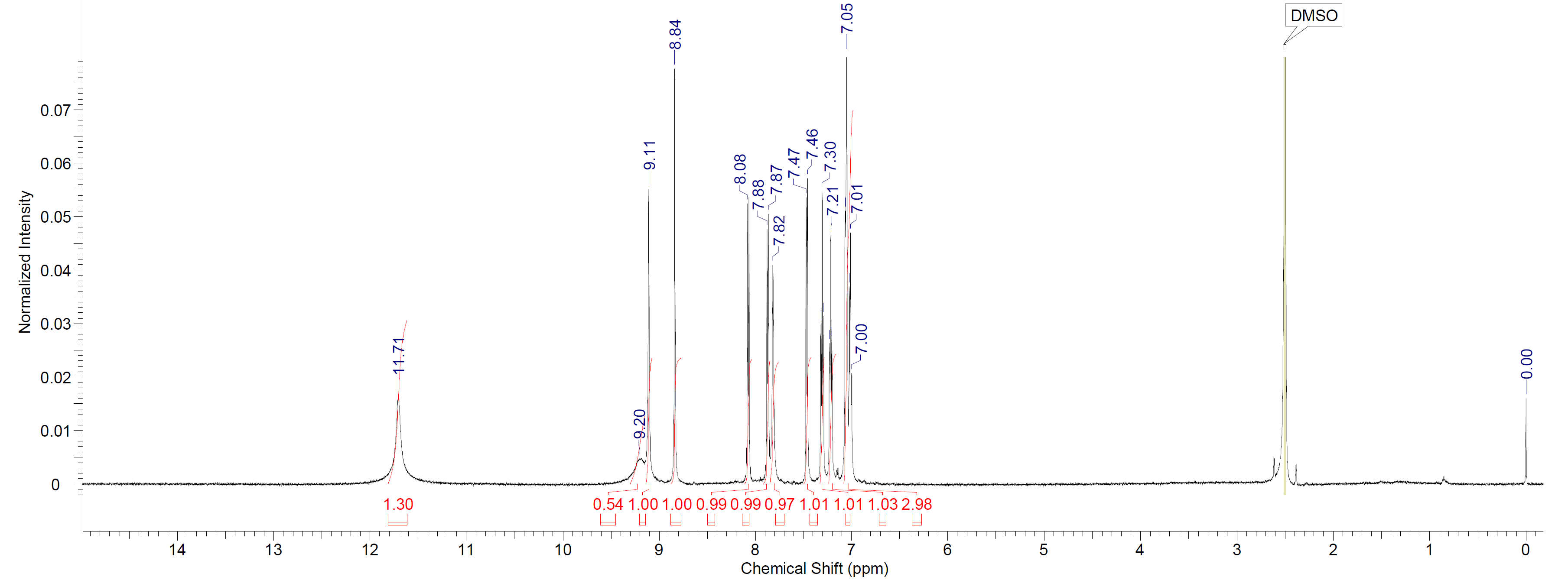

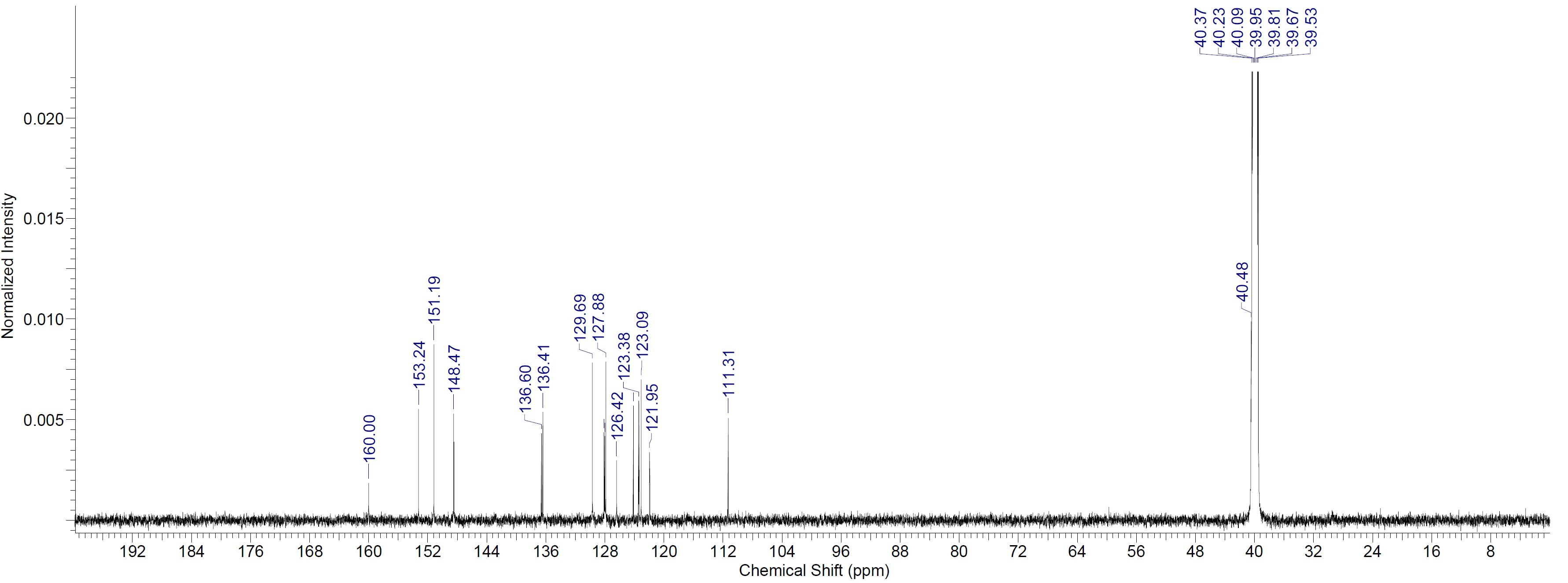


**L14f**

N'-(4-chlorophenyl)-2-(2,4-dihydroxypyrimidine-5-sulfonamido)phenyl urea.

A off white solid, yield: 89.50 %. Mp: 160-163°C. ^1^H NMR (600MHz ,DMSO*-d_6_*) *δ* 11.80 (s, 1 H), 9.52 (s, 1 H), 9.33 (s, 1 H), 8.43 (s, 1 H), 7.97 - 7.94 (m, 2 H), 7.81 (s, 1 H), 7.52 (d, *J* = 8.6 Hz, 2 H), 7.34 (d, *J* = 8.8 Hz, 2 H), 6.99 (d, *J* = 3.7 Hz, 2 H); ^13^C NMR (DMSO-*d*_6_) *δ* 162.78, 159.89, 153.01, 150.91, 148.26, 139.25, 137.18, 129.09, 128.29, 125.84, 125.72, 123.08, 121.44, 120.24, 111.30. ESI-HRMS calcd for C_17_H_14_ClN_5_O_5_S. [M - H]^+^ 434.0384, found: 434.0312.


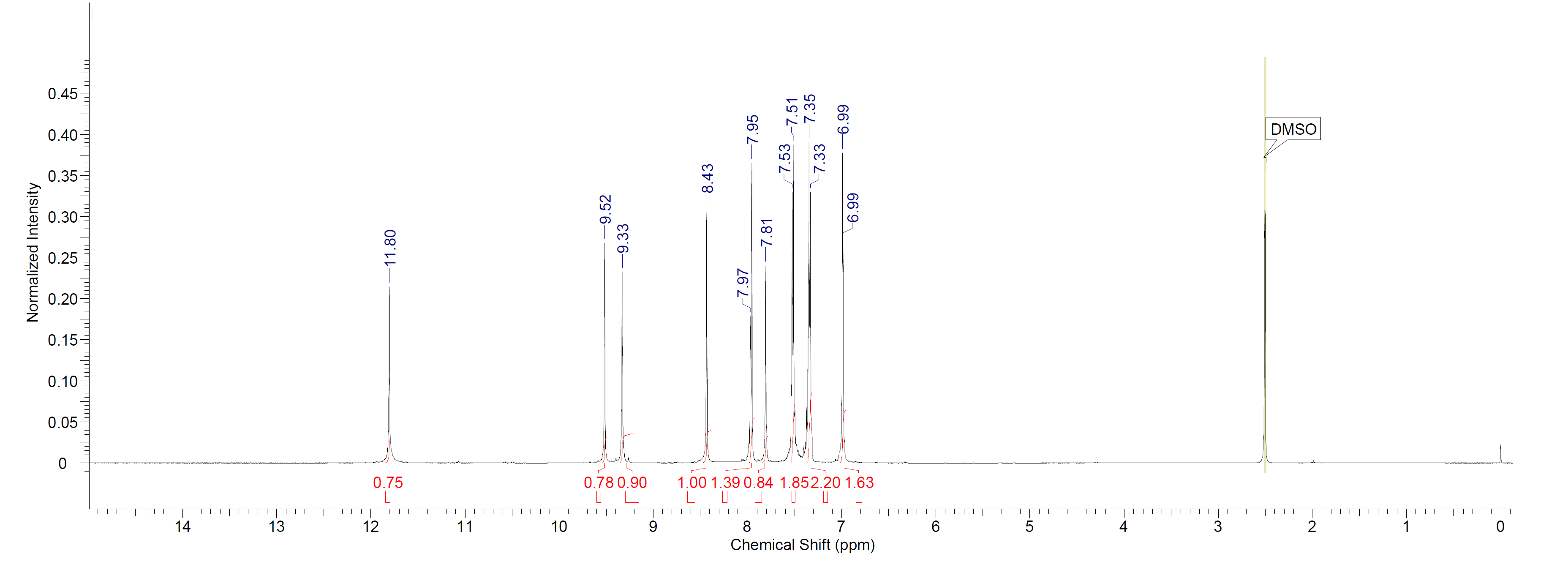

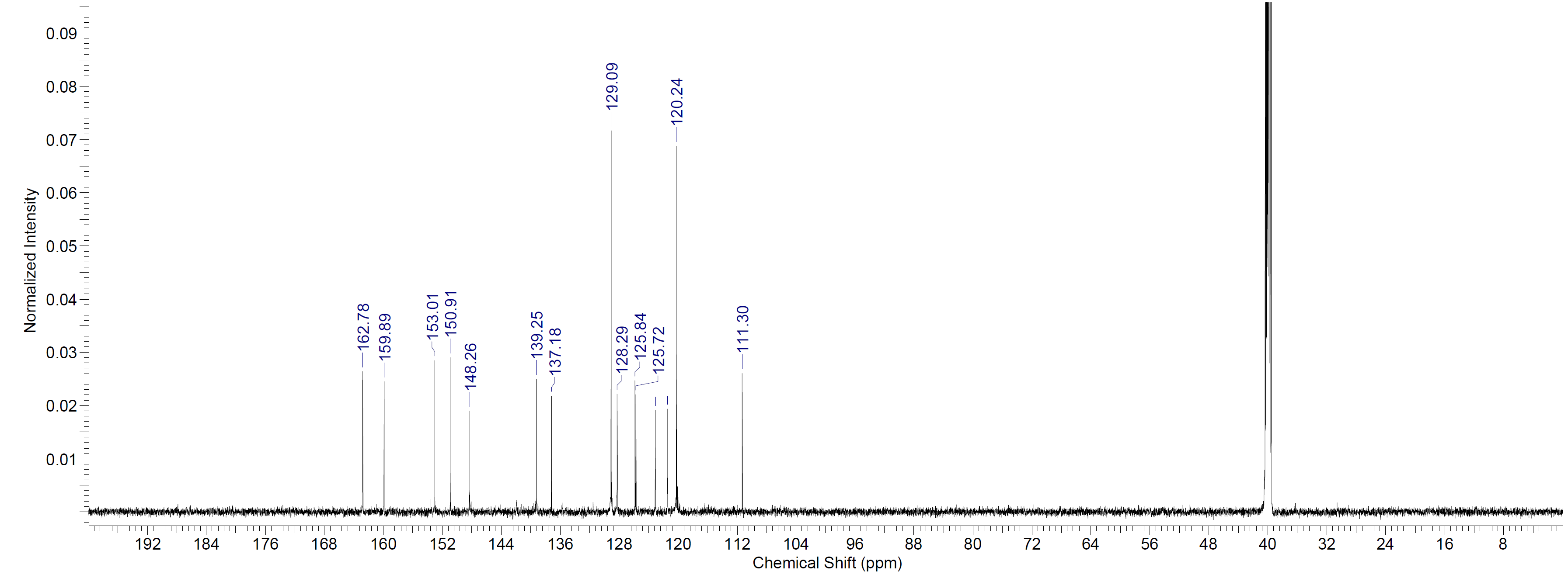


**L14g**

N'-(2-methylphenyl)-2-(2,4-dihydroxypyrimidine-5-sulfonamido)phenyl urea.

A off brown solid, yield: 78.80 %. Mp: 151-154°C. ^1^H NMR (600MHz ,DMSO-*d_6_*) *δ* 11.87 (br. s., 1 H), 11.77 (s, 1 H), 9.37 (s, 1 H), 9.05 (s, 1 H), 8.53 (s, 1 H), 7.81 (s, 1 H), 7.76 (d, *J* = 8.1 Hz, 1 H), 7.22 - 7.13 (m, 3 H), 7.10 (d, *J* = 7.2 Hz, 1 H), 7.02 - 6.95 (m, 2 H), 2.29 (s, 3 H); ^13^C NMR (150 MHz, DMSO-*d*_6_) *δ* 159.82, 153.93, 150.84, 147.99, 137.67, 136.48, 130.60, 129.09, 127.94, 127.83, 126.47, 123.53, 123.26, 122.69, 121.81, 111.63, 18.63. ESI-HRMS calcd for C_18_H_17_lN_5_O_5_S. [M - H]^+^ 414.0934, found: 414.0861.


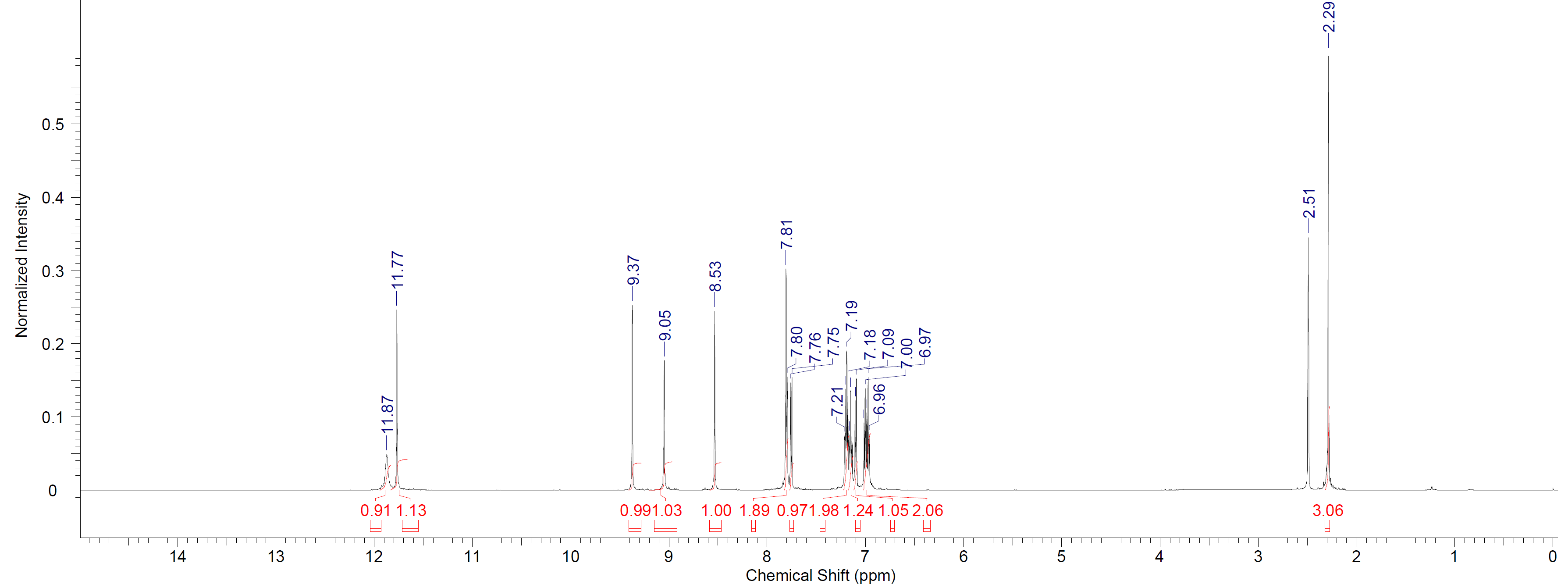

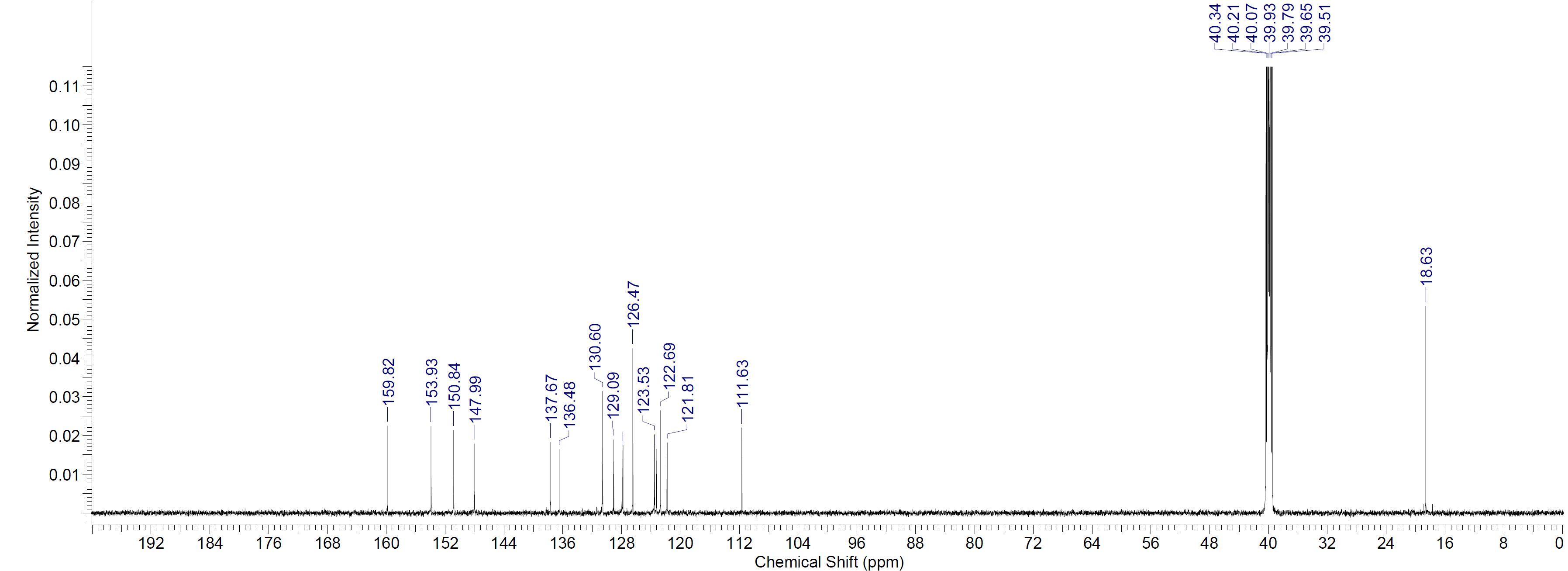


**L14h**

N'-(3-methylphenyl)-2-(2,4-dihydroxypyrimidine-5-sulfonamido)phenylurea.

A brown solid, yield: 81.50 %. Mp: 155-158°C. ^1^H NMR (600MHz ,DMSO-*d_6_*) *δ* 11.54 (br. s., 1 H), 9.18 (br. s., 1 H), 8.75 (br. s., 1 H), 8.02 (br. s., 1 H), 7.88 (br. s., 1 H), 7.32 (d, *J* = 19.1 Hz, 2 H), 7.17 (br. s., 2 H), 7.02 - 6.87 (m, 2 H), 6.79 (br. s., 1 H), 2.29 (br. s., 3 H); ^13^C NMR (150 MHz, DMSO-*d*_6_) *δ* 160.77, 153.02, 150.07, 148.39, 140.22, 138.36, 137.64, 129.05, 128.14, 127.88, 125.61, 122.98, 122.44, 120.49, 119.16, 115.81, 110.20, 21.72. ESI-HRMS calcd for C_18_H_17_lN_5_O_5_S. [M - H]^+^ 414.0939, found: 414.0866.


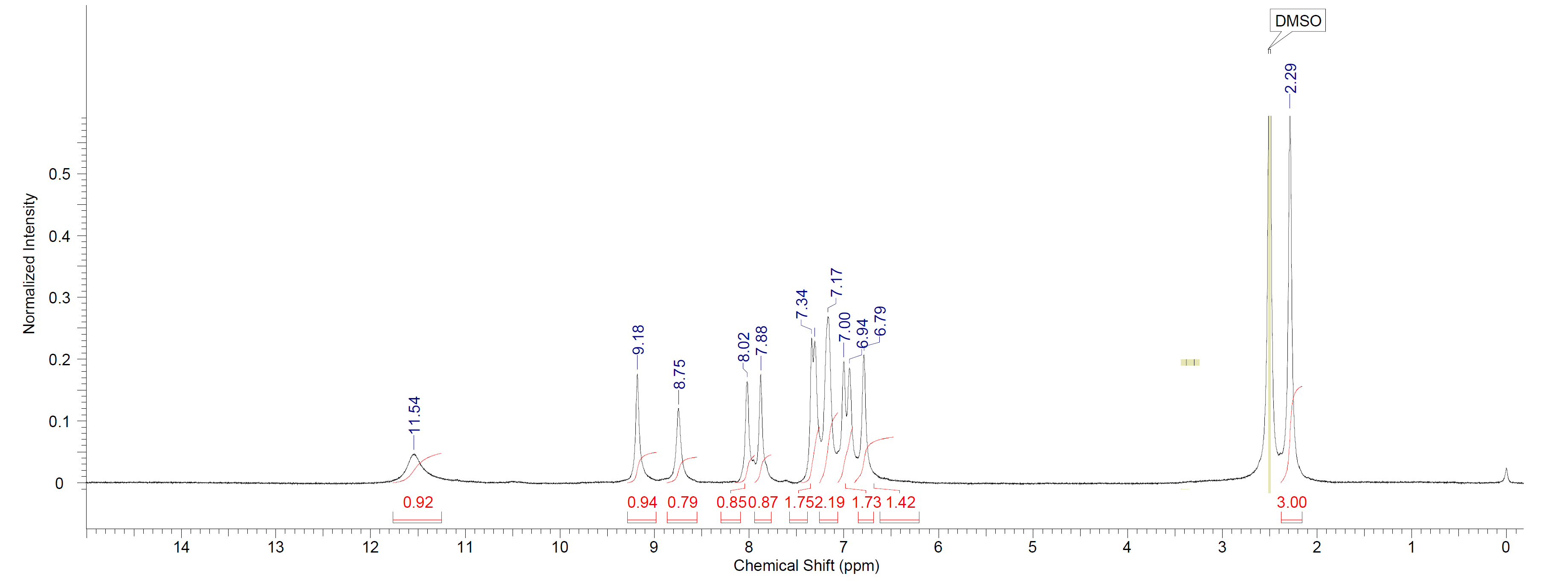

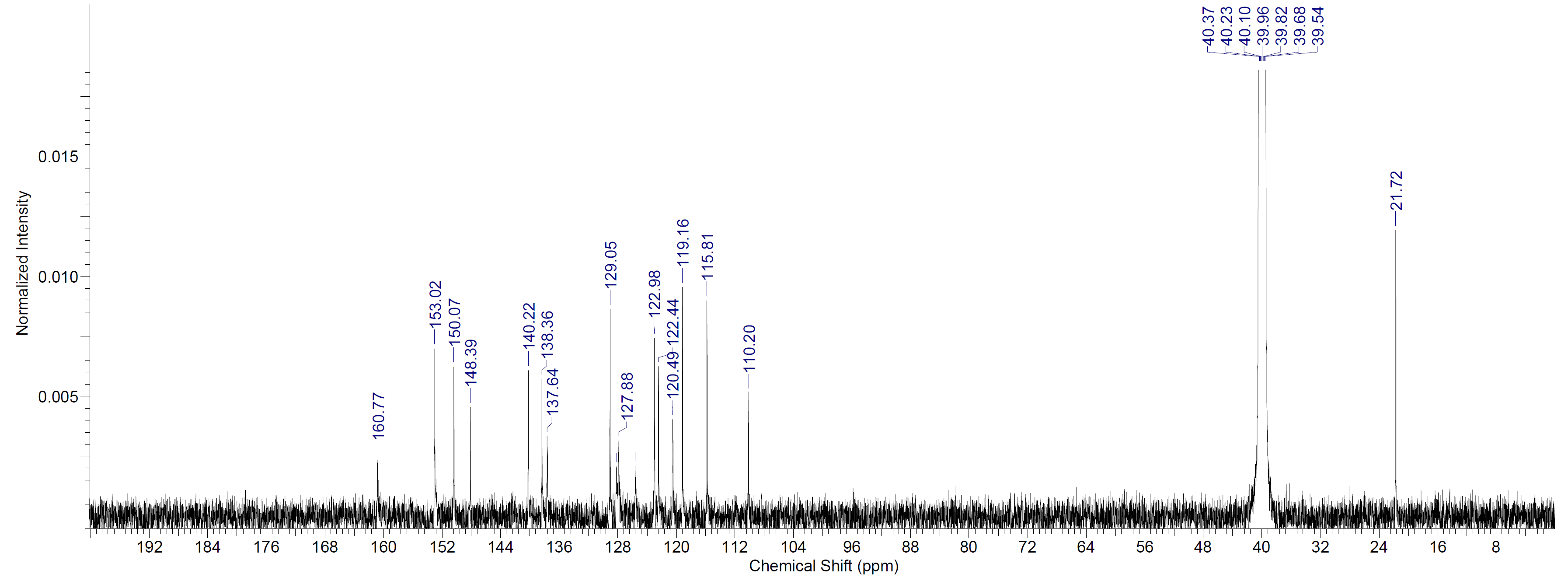


**L14i**

N'-(4-methylphenyl)-2-(2,4-dihydroxypyrimidine-5-sulfonamido)phenylurea.

A light brown solid, yield: 88.15 %. Mp: 165-169°C. ^1^H NMR (600MHz ,DMSO-*d_6_*) *δ* 11.82 (br. s., 1 H), 11.73 (br. s., 1 H), 9.32 (br. s., 1 H), 9.24 (s, 1 H), 8.39 (s, 1 H), 8.03 - 7.96 (m, 1 H), 7.82 (s, 1 H), 7.38 (d, *J* = 8.3 Hz, 2 H), 7.26 - 7.16 (m, 1 H), 7.10 (d, *J* = 8.4 Hz, 2 H), 7.01 - 6.91 (m, 2 H), 2.23 (br. s., 3 H); ^13^C NMR (150 MHz, DMSO-*d*_6_) *δ* 159.93, 153.11, 150.93, 148.29, 137.65, 137.60, 131.16, 129.64. 128.27, 128.28. 125.36, 122.70, 121.12, 118.86. 111.36, 20.83. ESI-HRMS calcd for C_18_H_17_lN_5_O_5_S. [M - H]^+^ 414.0950, found: 414.0868.


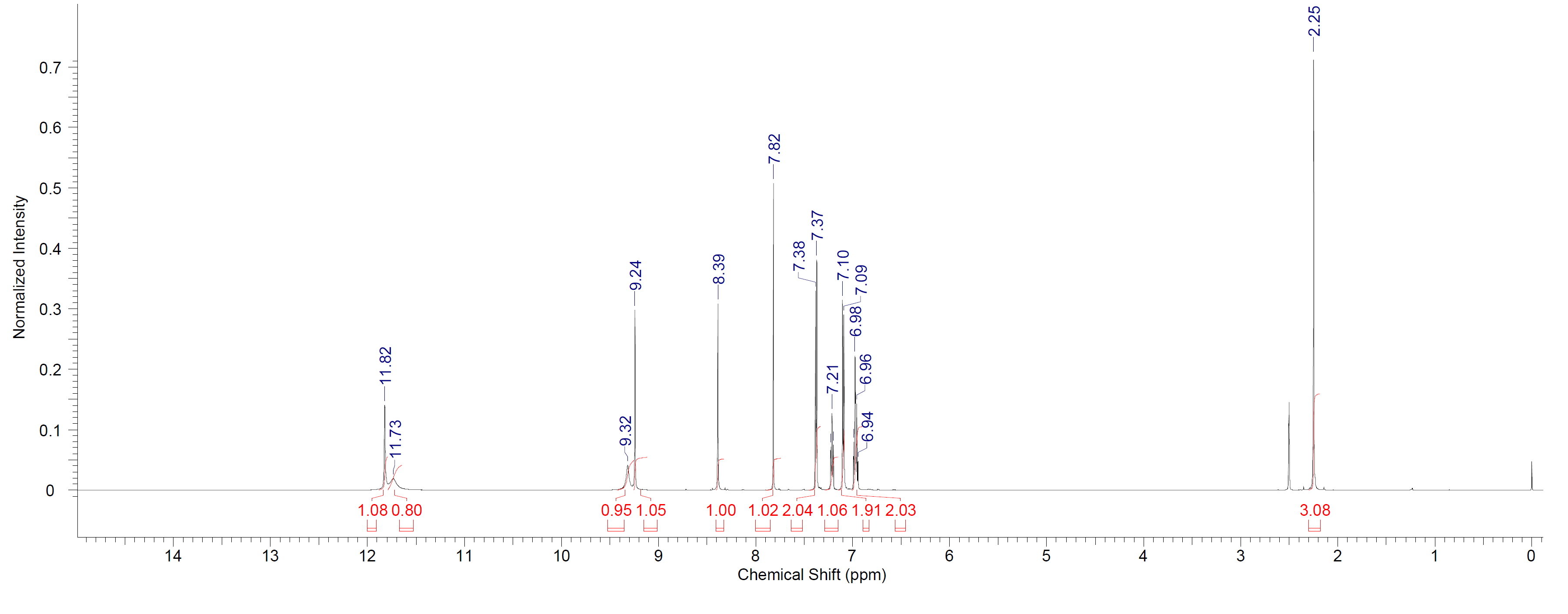

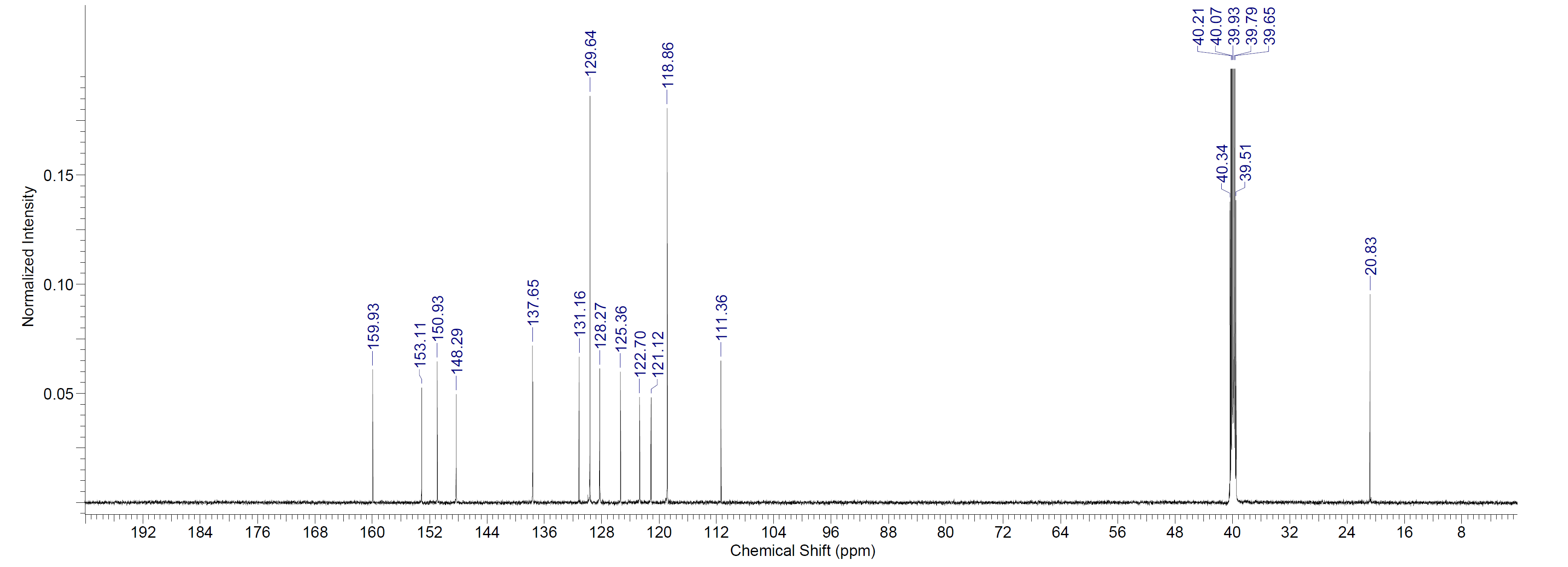


**15d**

N'-(2-chlorophenyl)-3-(2,4-dihydroxypyrimidine-5-sulfonamido)phenylurea.

A white solid, yield: 82.10 %. Mp: 159-162°C. ^1^H NMR (600MHz, DMSO-*d_6_*) *δ* 11.95 (d, *J* = 4.2 Hz, 1 H), 11.62 (s, 1 H), 10.17 (s, 1 H), 9.97 (br. s., 1 H), 8.52 (s, 1 H), 8.16 (dd, *J* = 1.5, 8.3 Hz, 1 H), 8.06 (d, *J* = 5.7 Hz, 1 H), 7.44 (dd, *J* = 1.5, 8.1 Hz, 1 H), 7.37 (s, 1 H), 7.33 - 7.25 (m, 1 H), 7.20 - 7.10 (m, 2 H), 7.03 (d, *J* = 1.6, 7.7 Hz, 1 H), 6.72 (td, *J* = 1.6, 7.7 Hz, 1 H); ^13^C NMR (150 MHz, DMSO-*d*_6_) *δ* 159.05, 152.66, 150.85, 148.91, 140.79, 138.52, 136.49, 129.75, 129.64, 127.95, 123.71, 122.65, 122.08, 113.64, 113.34, 111.10, 109.31. ESI-HRMS calcd for C_17_H_14_ClN_5_O_5_S. [M - H]^+^ 434.0397, found: 434.0328.


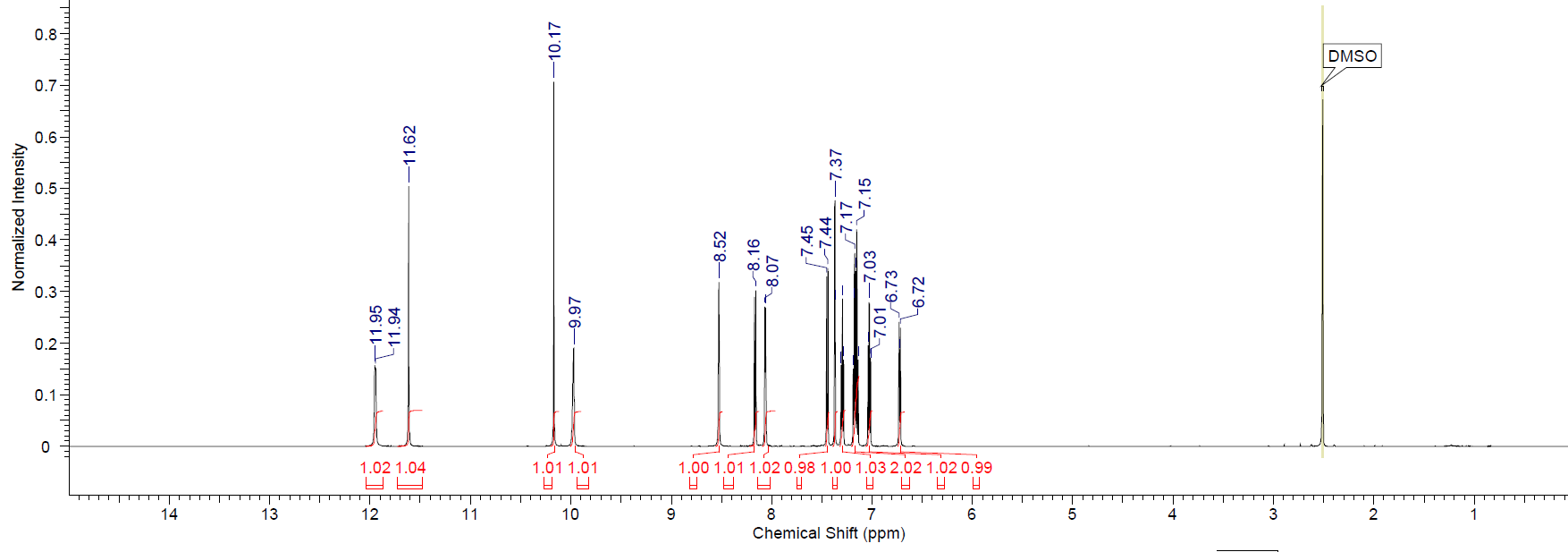


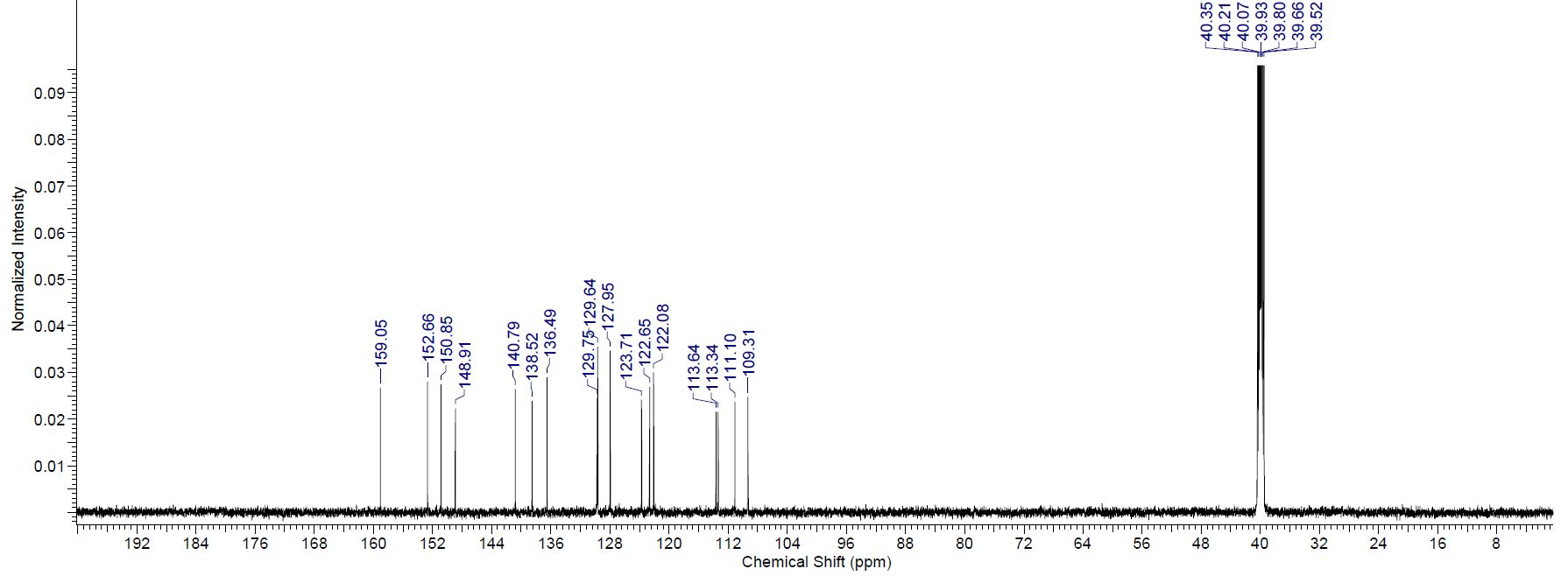


**L15e**

N'-(3-chlorophenyl)-3-(2,4-dihydroxypyrimidine-5-sulfonamido)phenylurea.

A white solid, yield: 83.40 %. Mp: 162-166°C. ^1^H NMR (600MHz, DMSO-*d_6_*) *δ* 11.93 (br. s., 1 H), 11.62 (s, 1 H), 10.15 (s, 1 H), 9.83 (br. s., 1 H), 9.57 (br. s., 1 H), 8.06 (br. s., 1 H), 7.67 (s, 1 H), 7.37 (s, 1 H), 7.33 - 7.27 (m, 2 H), 7.17 - 7.09 (m, 2 H), 7.00 (dd, *J* = 1.9, 7.4 Hz, 1 H), 6.71 (d, *J* = 7.9 Hz, 1 H); ^13^C NMR (150 MHz, DMSO-*d*_6_) *δ* 159.05, 152.92, 150.86, 148.88, 141.94, 140.71, 138.52, 133.61, 130.89, 129.72, 121.64, 117.51, 116.67, 113.64, 113.28, 111.12, 109.42. ESI-HRMS calcd for C_17_H_14_ClN_5_O_5_S. [M - H]^+^ 434.0384, found: 434.0312.


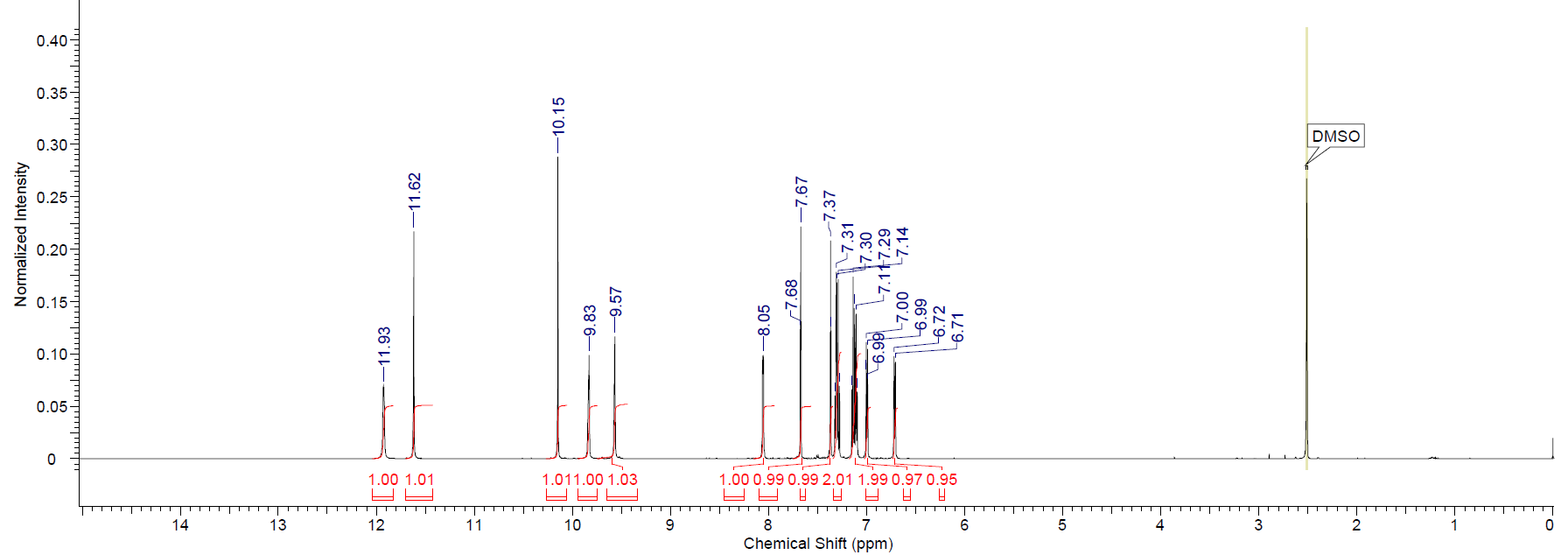

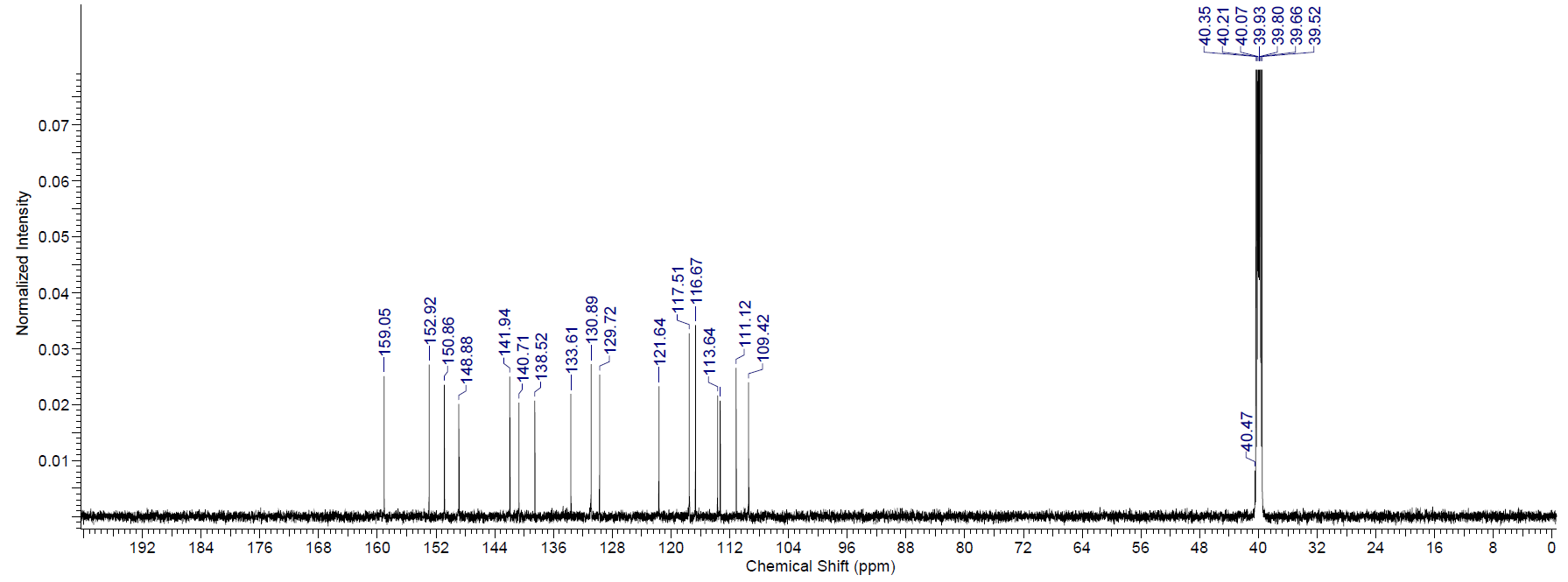


**L15f**

N'-(4-chlorophenyl)-3-(2,4-dihydroxypyrimidine-5-sulfonamido)phenylurea.

A off white solid, yield: 84.50 %. Mp: 169-171°C. ^1^H NMR (600MHz ,DMSO-*d_6_*) *δ* 11.91 (d, *J* = 2.9 Hz, 6 H), 11.62 (s, 1 H), 10.15 (s, 1 H), 9.57 (s, 1 H), 9.42 (s, 1 H), 8.06 (d, *J* = 4.6 Hz, 1 H), 7.50 (d, *J* = 8.6 Hz, 2 H), 7.40 - 7.36 (m, 1 H), 7.35 - 7.28 (m, 2 H), 7.20 - 7.11 (m, 1 H), 7.11 - 7.04 (m, 1 H), 6.70 (d, *J* = 7.7 Hz, 1 H); ^13^C NMR (150 MHz, DMSO-*d*_6_) *δ *159.05, 152.93, 150.89, 148.95, 140.77, 139.33, 138.52, 129.70, 129.07, 125.52, 119.85, 113.60, 113.21, 111.13, 109.32. ESI-HRMS calcd for C_17_H_14_ClN_5_O_5_S. [M - H]^+^ 434.0385, found: 434.0313.


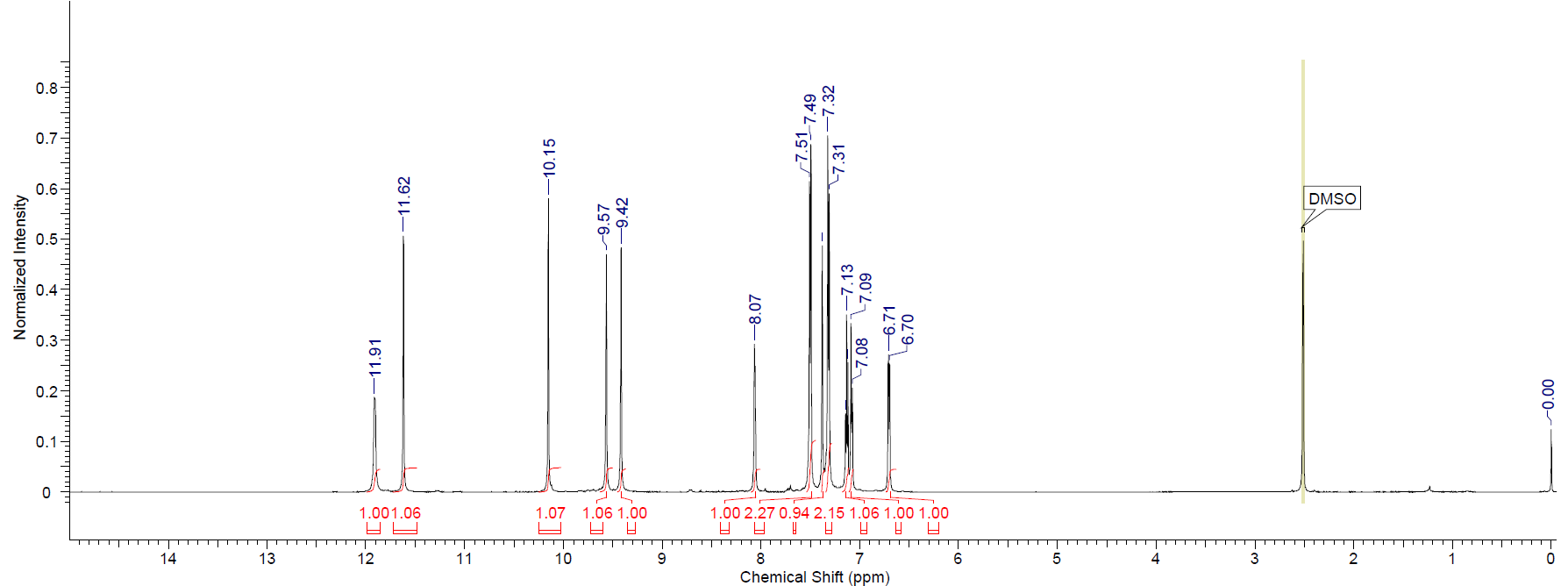

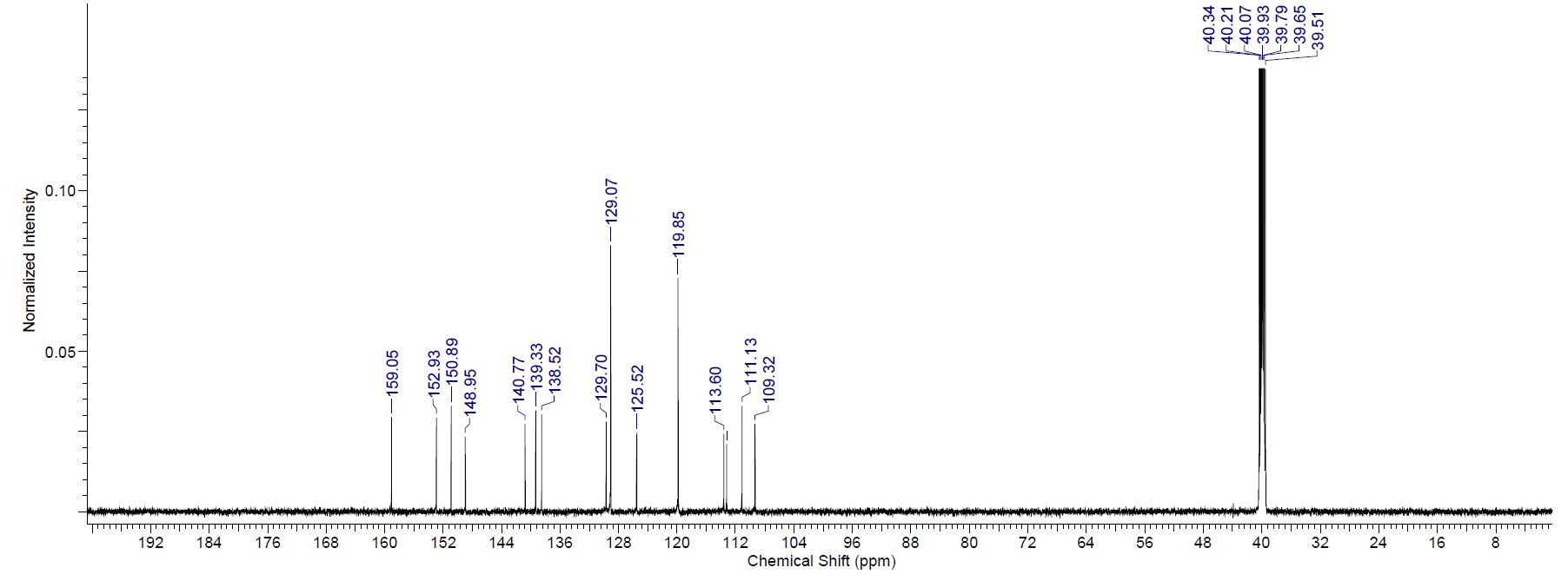


**L15g**

N'-(2-methylphenyl)-3-(2,4-dihydroxypyrimidine-5-sulfonamido)phenyl urea.

A white solid, yield: 72.60 %. Mp: 163-167°C. ^1^H NMR (600MHz, DMSO-*d_6_*) *δ* 11.57 (br. s., 1 H), 10.10 (br. s., 1 H), 9.09 (s, 1 H), 8.04 (s, 1 H), 7.89 (s, 1 H), 7.86 (d, *J* = 8.1 Hz, 1 H), 7.29 (s, 1 H), 7.18 - 7.12 (m, 4 H), 6.94 (t, *J* = 7.2 Hz, 1 H), 6.71 (d, *J* = 7.7 Hz, 1 H), 2.24 (s, 3 H); ^13^C NMR (150 MHz, DMSO-*d*_6_) *δ* 159.06, 152.88, 151.12, 149.07, 140.96, 138.57, 137.83, 130.63, 129.81, 127.73, 126.65, 123.05, 121.30, 113.54, 113.21, 111.08, 109.23, 18.37. ESI-HRMS calcd for C_18_H_17_lN_5_O_5_S. [M - H]^+^ 414.0949, found: 414.0877.


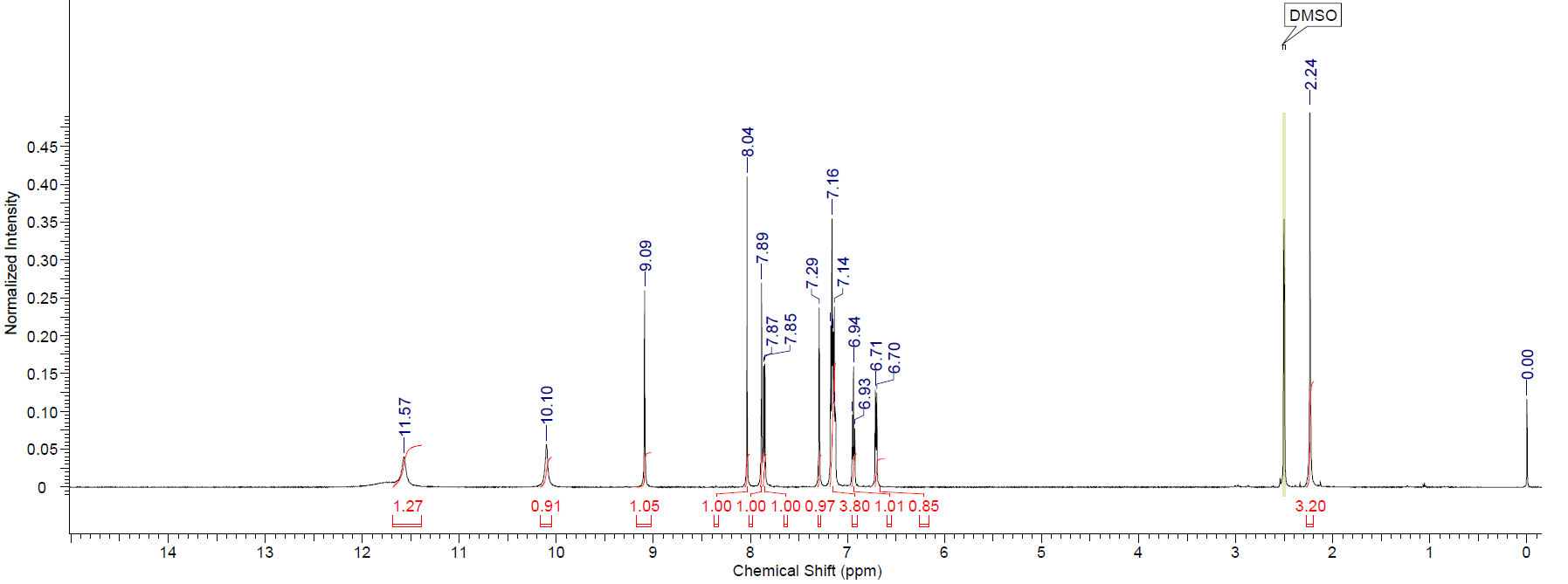

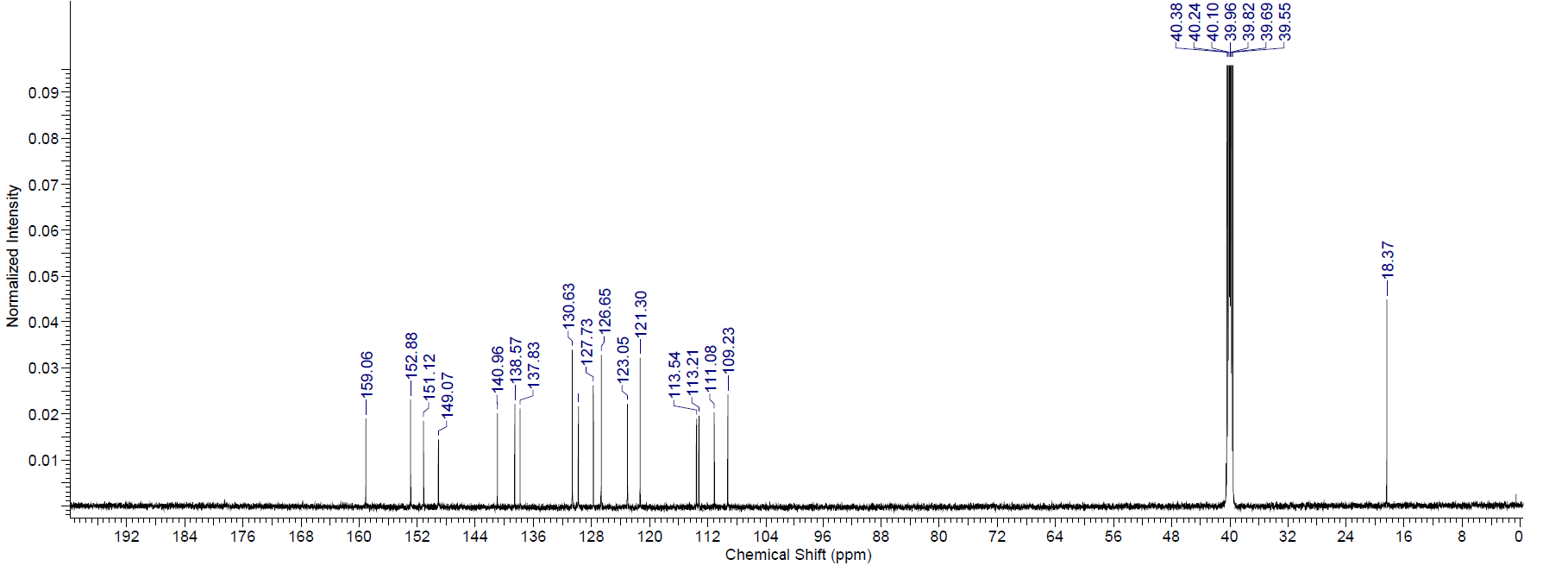


**L15h**

N'-(3-methylphenyl)-3-(2,4-dihydroxypyrimidine-5-sulfonamido)phenyl urea.

A off white solid, yield: 70.00 %. Mp: 165-169°C. ^1^H NMR (600MHz ,DMSO-*d_6_*) *δ* 11.84 (d, *J* = 5.3 Hz, 5 H), 11.62 (s, 1 H), 10.12 (s, 1 H), 9.04 (s, 1 H), 8.88 (s, 1 H), 8.05 (d, *J* = 6.1 Hz, 1 H), 7.34 (s, 1 H), 7.30 - 7.24 (m, 2 H), 7.18 - 7.07 (m, 3 H), 6.78 (d, *J* = 7.3 Hz, 1 H), 6.70 (d, *J* = 7.5 Hz, 1 H), 2.28 (s, 3 H); ^13^C NMR (150 MHz, DMSO-*d*_6_) *δ* 159.03, 152.88, 150.91, 148.83, 140.92, 140.11, 138.50, 138.38, 129.70, 129.09, 122.95, 119.00, 115.71, 113.65, 113.22, 111.22, 109.42, 21.70. ESI-HRMS calcd for C_18_H_17_lN_5_O_5_S. [M - H]^+^ 414.0951, found: 414.0880.


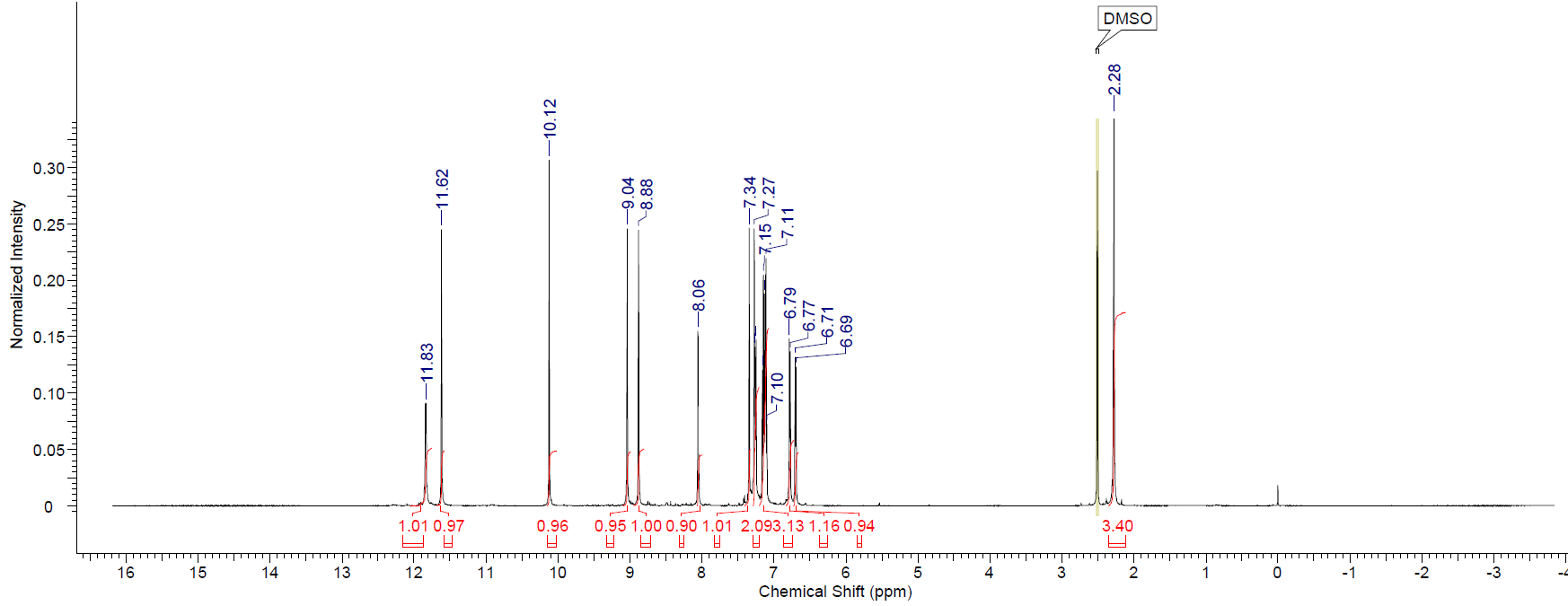

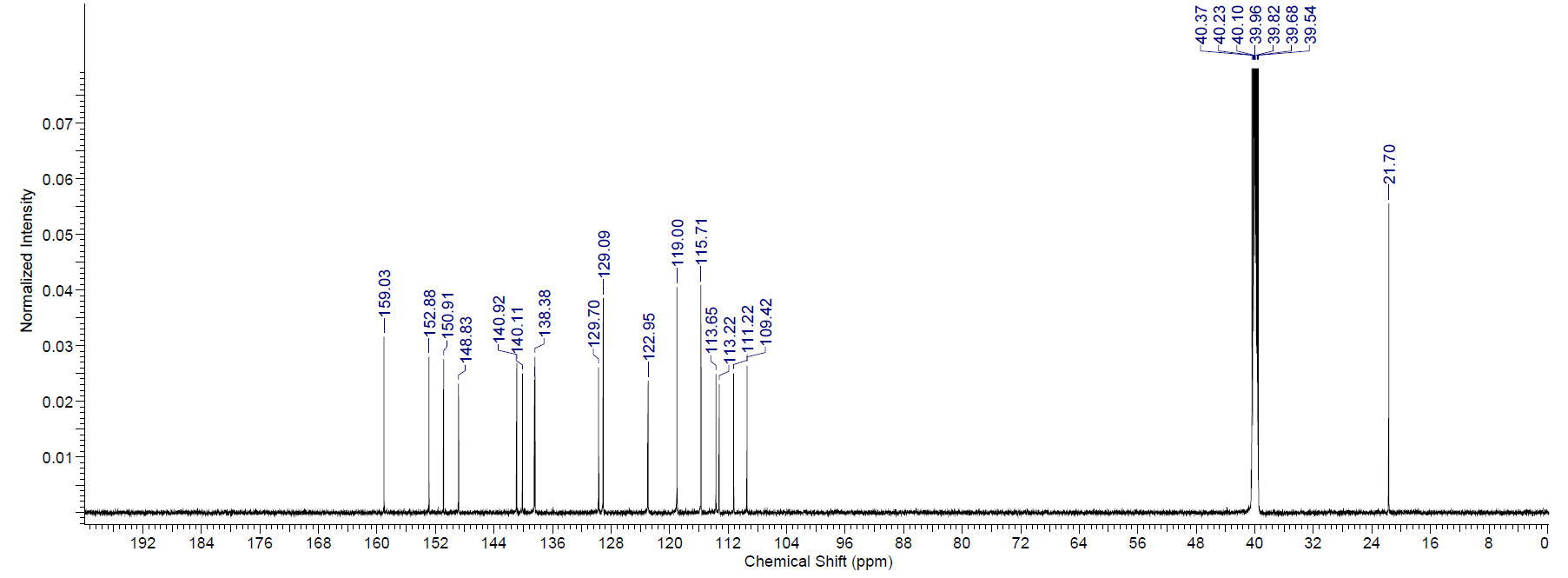


**L15i**

N'-(4-methylphenyl)-3-(2,4-dihydroxypyrimidine-5-sulfonamido)phenyl urea.

A white solid, yield: 78.50 %. Mp: 173-175°C. ^1^H NMR (600MHz, DMSO-*d_6_*) *δ* 11.81 (br. s., 1 H), 11.62 (br. s., 1 H), 10.13 (br. s., 1 H), 8.70 (br. s., 1 H), 8.52 (br. s., 1 H), 8.05 (br. s., 1 H), 7.34 (br. s., 3 H), 7.13 – 7.08 (m, 4 H), 6.71 (d, *J* = 7.3 Hz, 1 H), 2.24 (s, 3 H); ^13^C NMR (150 MHz, DMSO-*d*_6_) *δ* 159.02, 152.84, 150.94, 148.88, 140.88, 138.50, 137.49, 131.12, 129.74, 129.65, 118.73, 113.68, 113.19, 111.19, 109.35, 20.82. ESI-HRMS calcd for C_18_H_17_lN_5_O_5_S. [M - H]^+^ 414.0936, found: 414.0863.


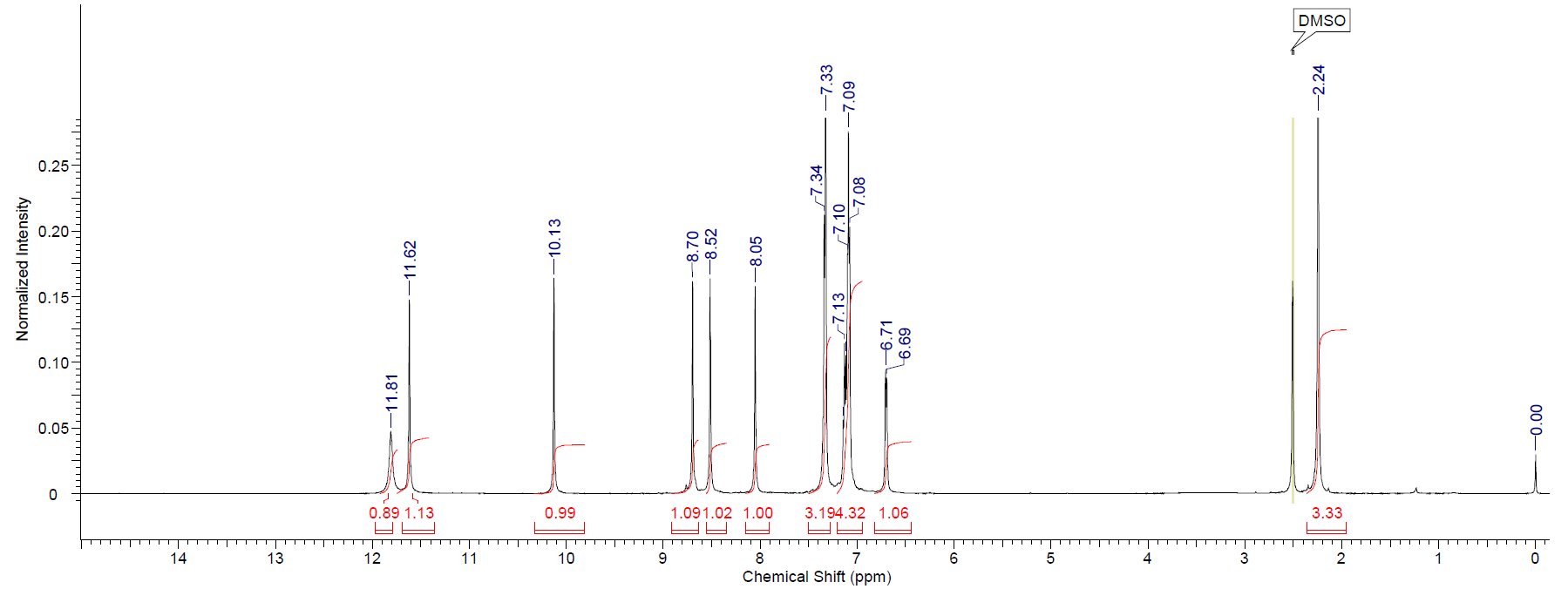

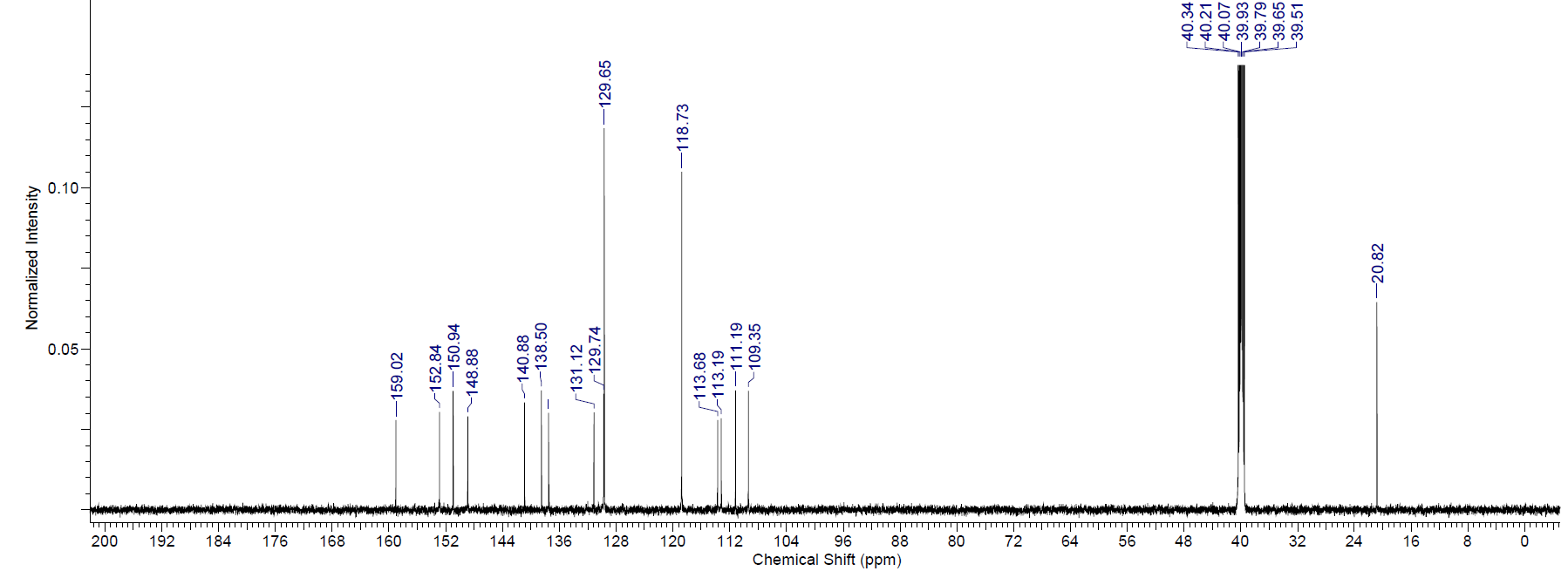


**HPLC analysis of stability of compound L14e in cell culture medium.**


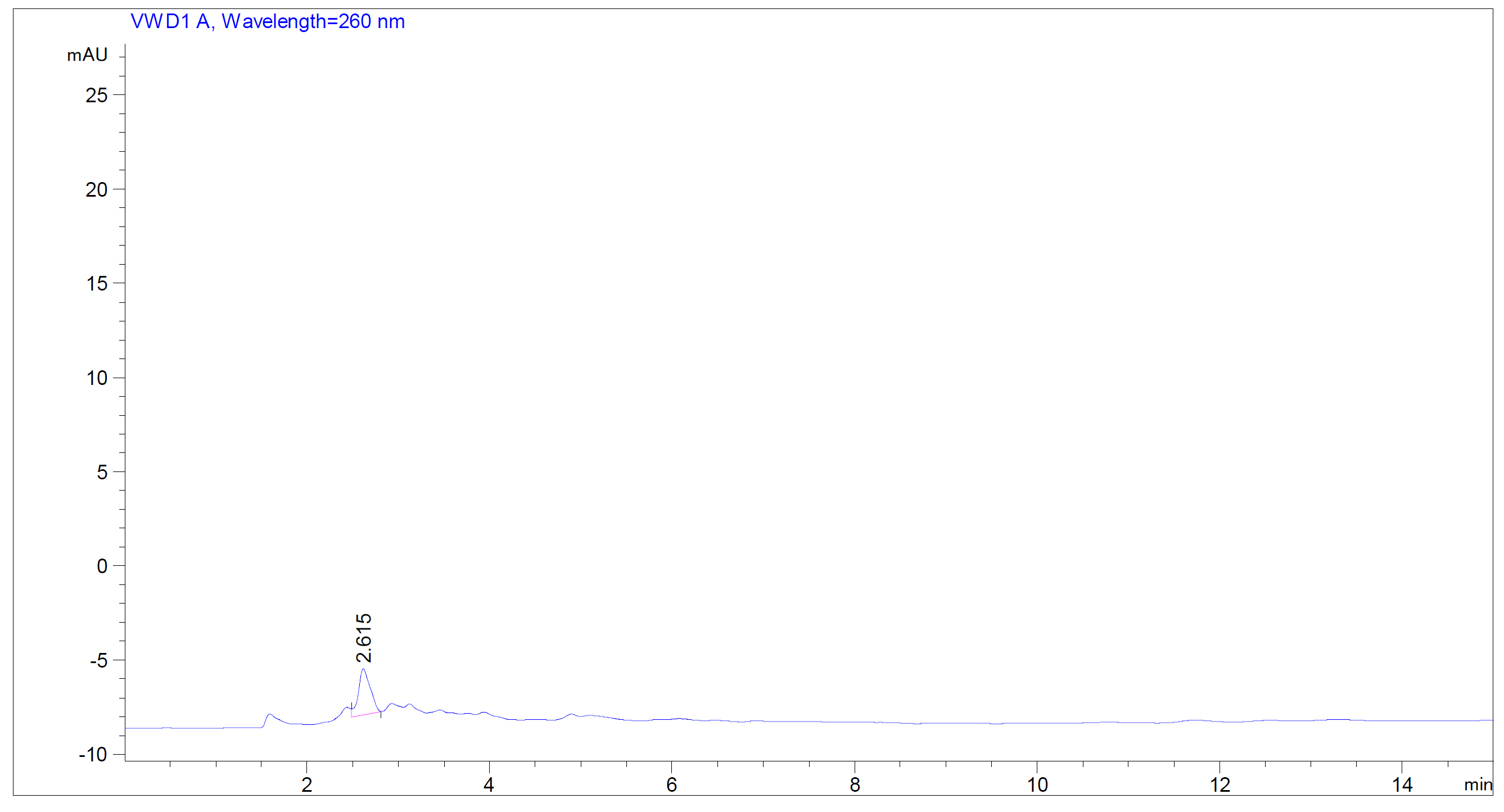


(a)


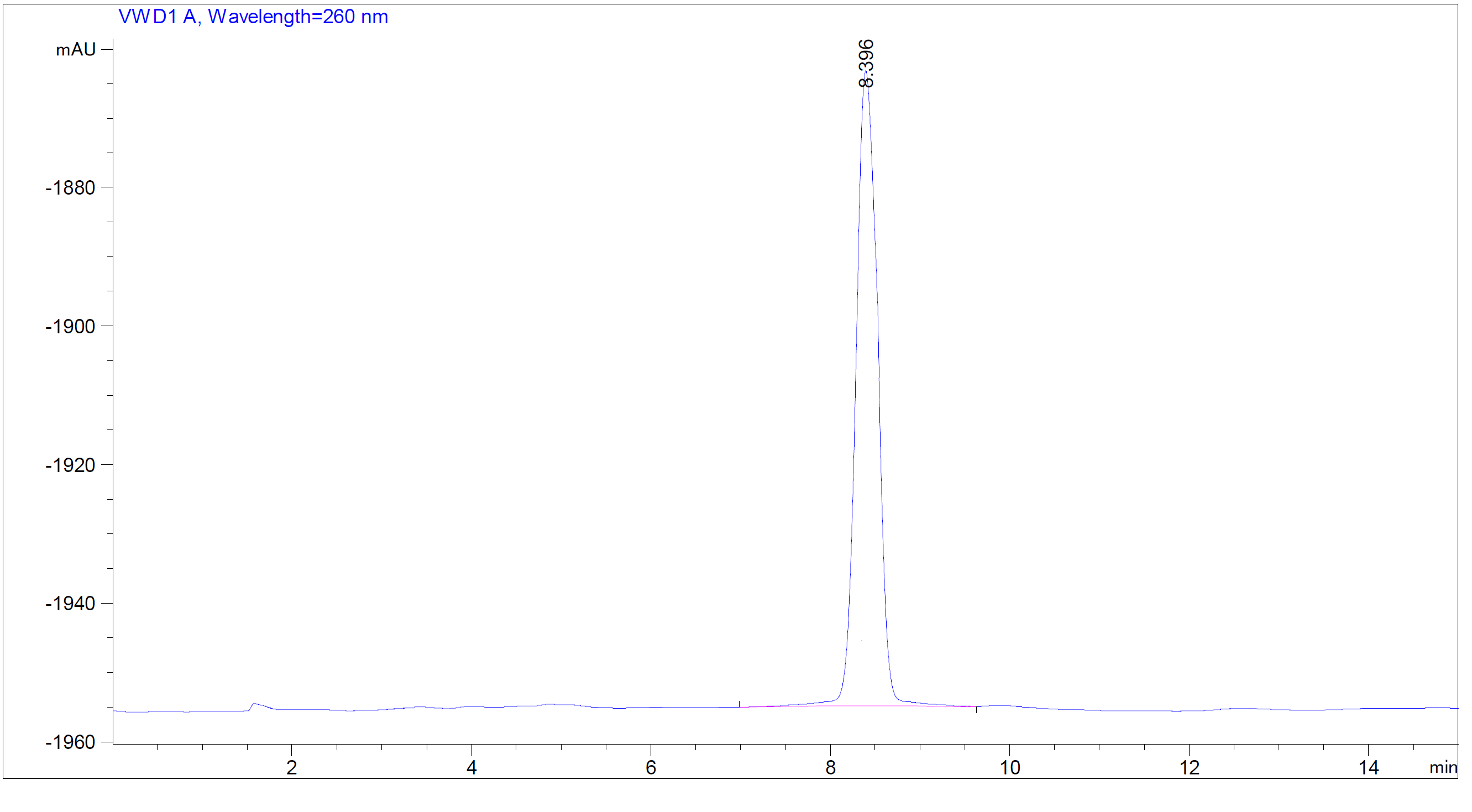


(b)


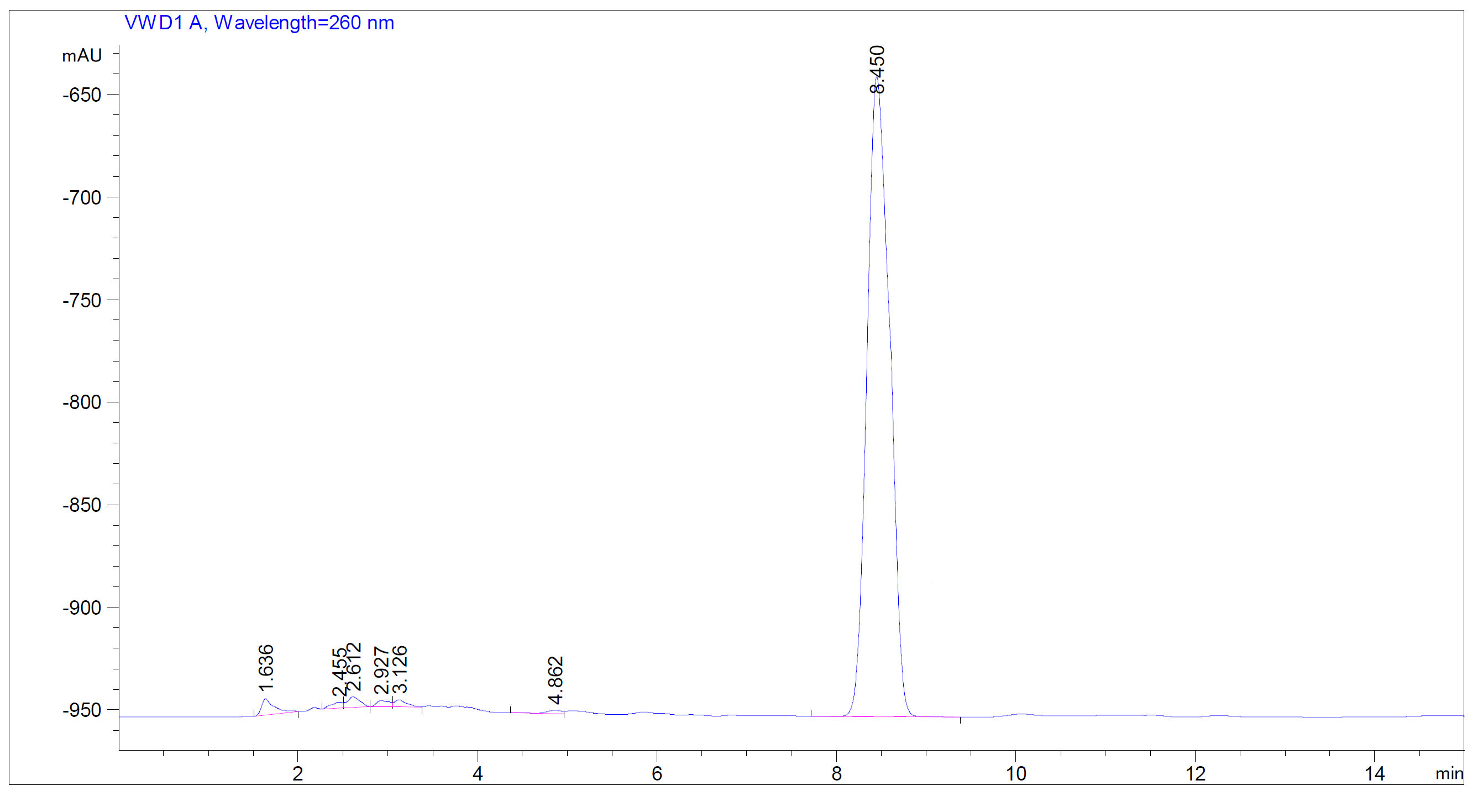


(c)

**Figure S1.** HPLC analysis of stability of compound **L14e** in cell culture medium. (a): Cell culture media. (b): Compound **L14e** standard. (c): Cell culture media containing compound **L14e**.

| Compound **L14e**  Concentration(µM**)** | HPLC-UV Measure Concentration(µM**)**^a^ | | | |
| --- | --- | --- | --- | --- |
|  | 1h | 12h | 24h | 36h |
| 0.625 | 0.621±0.005 | 0.623±0.001 | 0.625±0.002 | 0.625±0.001 |
| 1.25 | 1.246±0.005 | 1.239±0.003 | 1.253±0.007 | 1.242±0.009 |
| 2.5 | 2.510±0.020 | 2.506±0.021 | 2.430±0.002 | 2.483±0.050 |
| 5 | 5.001±0.010 | 4.963±0.005 | 5.012±0.023 | 4.980±0.020 |
| 10 | 10.000±0.049 | 9.908±0.100 | 9.930±0.100 | 9.850±0.150 |

**Table S1.** HPLC measurement results of compound **L14e** in cell culture medium.

a: HPLC-UV Measure Concentration: Mean ± SD.

**MTT bioassay**

Total cells in the logarithmic growth phase were collected, digested using 0.25% pancreatic enzymes, and then diluted into single-cell suspensions using phosphate-buffered solution (PBS). These cells were seeded into 96-well plates at 4× 10^3^-5× 10^3^ cells per well (100 µL per well) and then A549, H460, OVCAR-3, SGC7901, MDA-MB-231, HCT116 and HepG2 were cultured at 37°Cin an incubator containing 5% CO_2_. After cell complete attachment, 100 µL of different concentrations of compounds (0.625 μM, 1.25 μM, 2.5μM, 5 μM, 10 μM) were added to the cells after the nutrient solution in each well was discarded, while no compound except 0.1% DMSO was added to cells in the control group; at the same time, each concentration was set up to 5 wells. In addition, 5 wells in each 96-well plate only received 100 µL of nutrient solution as a blank control. The plates were taken out after the cells were incubated with compounds for 24 h, and 20 µL of MTT solution (5 mg/mL) (Wuhan Boster Biotechnology Limited Company, Wuhan, China; item No. AR1156) was added into all the wells. After another time of incubation for 4 h at 37°C, the nutrient solution in each well was discarded slowly, and 150 µL of DMSO was added to each well. The plates were shaken on a shaking table for 20 min. When the crystals were fully dissolved, the blank wells were used to zero the plate reader. The optical density (OD) of each well was detected at 490 nm. The IC_50_ value was determined as the concentration of the inhibitor that caused 50% inhibition. Using probability unit and weighted regression method to calculate the IC_50_ value of compounds.

**Annexin V/propidium iodide (PI) staining.**

The density of the A549 cells and H460 in the logarithmic growth phase was adjusted to 1 × 10^6^ cells/mL, and then the cell suspension was seeded into a 6-well plate (Becton Dickinson and Company, USA). A total of 1 mL of cell suspension and 1 mL of compound **L14e** at different concentrations were added into each well with the final compound **L14e** concentration in each group as 0 μM, 0.5 μM, 1.0 μM, and 1.5μM. After cells were incubated for 24 h at 37°C, 1 mL of cell suspension per well was collected and centrifuged at 1000 r/min for 10 min at 4°C, and the supernatant was removed. Then, 1 mL of cold PBS was added to the cell pellet, and was shaken lightly to re-suspend the cells. The cells were centrifuged at 1000 r/min at 4°C for 10 min, and the supernatant was removed. These steps were repeated twice. The cells were re-suspended in 150µL of PBS, 10 µL of fluorescein isothiocyanate (FITC)-labeled Annexin V (Annexin V-FITC) (Promega Corporation, USA) and 5 µL of PI (Promega Corporation, USA) were added. The mixture was gently mixed and incubated in the dark at room temperature for 10 min then stained cells were analyzed using flow cytometry to test the levels of apoptotic cells (Becton Dickinson and Company, USA; FACS Calibur).

**Western blot analysis**

A549 cells, H460 cells and HUVECs were treated with different concentrations of compound **L14e** for 24 h, and collected to establish their respective treatment groups. The cell density is 1 × 10^7^ cells per flask (Becton Dickinson and Company, USA), and the group without compound 10l is used as the control group. A protein extraction reagent (Beyotime Biotechnology Company, Shanghai, China) was used to extract the proteins, and a BCA kit (Beyotime Biotechnology Company, Shanghai, China) was applied to measure the protein levels.

Extracted proteins were separated from each other using polyacrylamide electrophoresis on a 10 % gel (Beyotime Biotechnology Company, Shanghai, China) and transferred to a polyvinylidene fluoride (PVDF) membrane. When the membrane transfer finished, the membranes were placed on a horizontal shaking table and blocked for 1 h with PBS containing either 5% bovine serum albumin (BSA) or dissolved skim milk powder. The sealed PVDF membrane was removed and incubated at 4°C overnight with the following primary antibodies (100 μL/cm^2^). The secondary antibody was diluted in 5% BSA (Beyotime Biotechnology Company, Shanghai, China) at a density of 100 μl/cm^2^ and was added to the membranes, which were then sealed and incubated on a shaking table at room temperature for 1h. Equal volumes of detection reagents A and B were mixed in a petri dish with no strong light throughout the procedure. The PVDF membrane (Bio-Rad Company, USA) immersed in PBS was placed in ECL working solution for color development, and was scanned using the Bio-Rad Gel Doc 2000 gel imaging system. Image analysis and processing were conducted by Image Lab. Gray values of protein bands were quantitatively analyzed, with the gray value of β-actin(GAPDH) band as the reference.

**HE staining.**


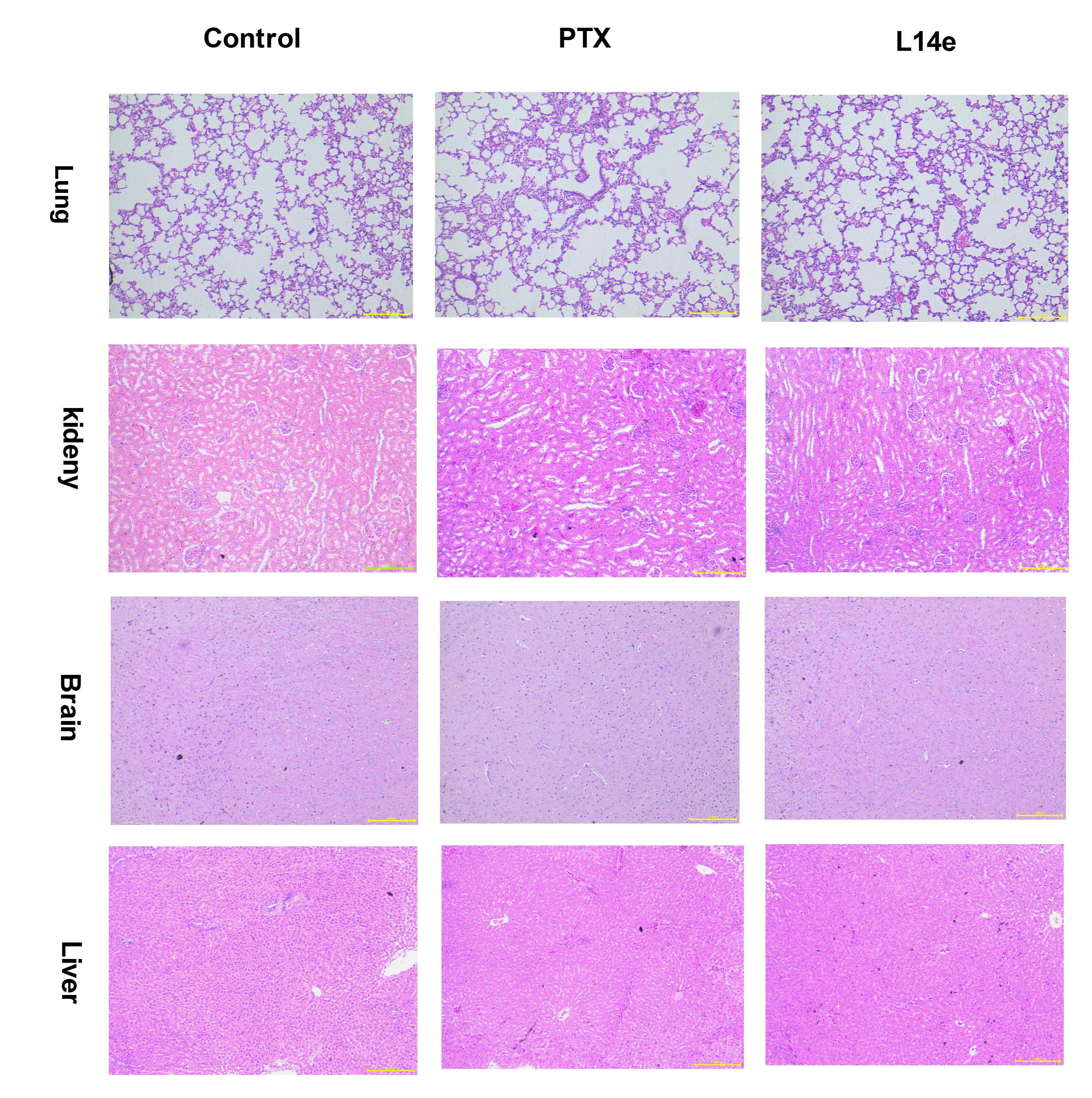


**Figure S2.** The histopathological changes in liver, lung, brain, and kidney from mice treated with PTX or **L14e** were evaluated by using HE-stained section of the tissues. The representative images of HE-stained section of the tissues were shown.

**P value:**

**(Figure 3)**

**
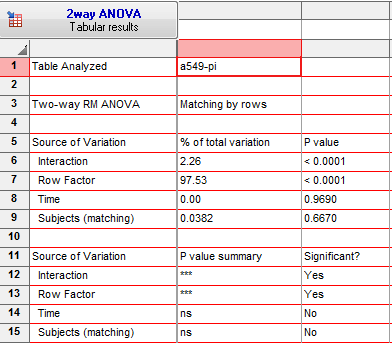
**

**
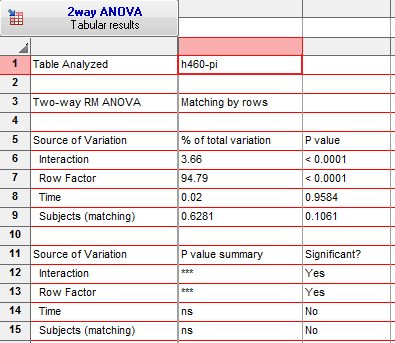
**

**(Figure 4(A))**

**
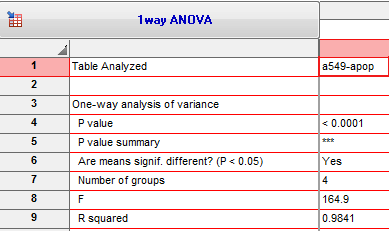
**

**
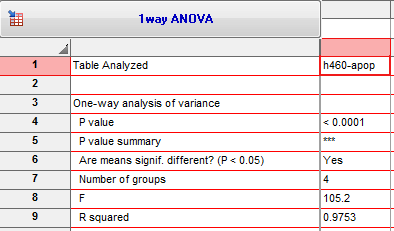
**

**(Figure 4(B))**

**
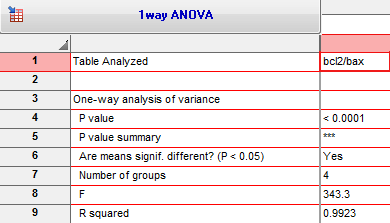
**

**
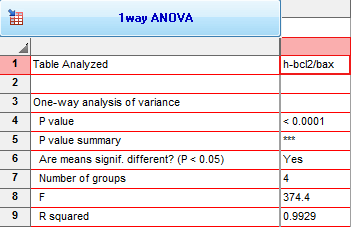
**

**
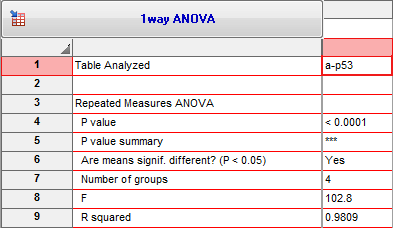
**

**
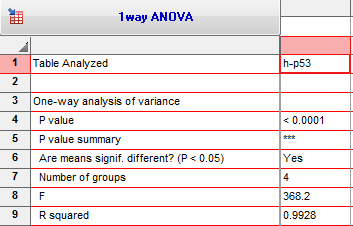
**

**
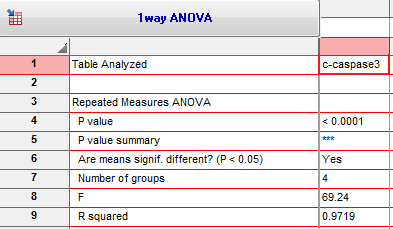
**

**
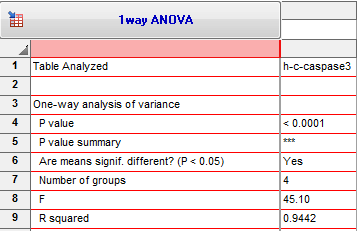
**

**(Figure 5(A))**

**
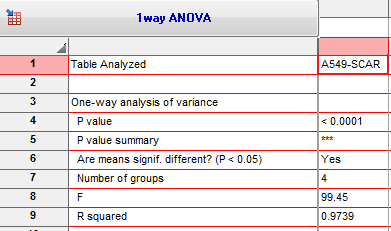
**

**
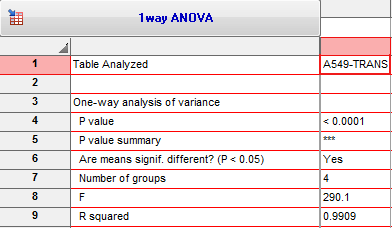
**

**
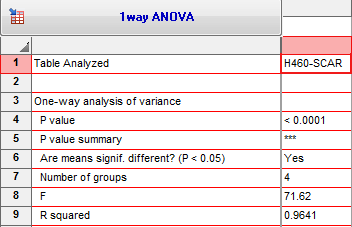
**

**
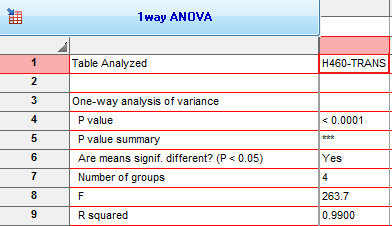
**

**(Figure 6(A))**

**
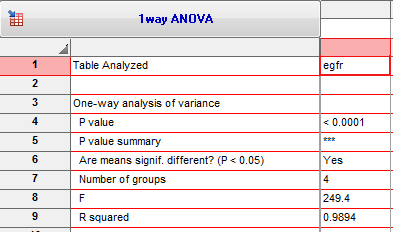
**

**
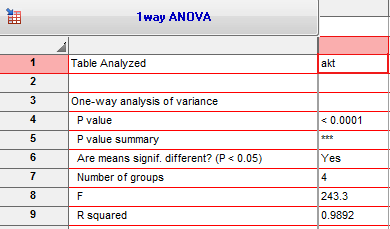
**

**
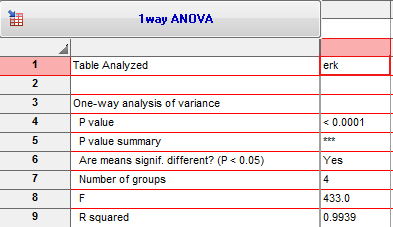
**

**
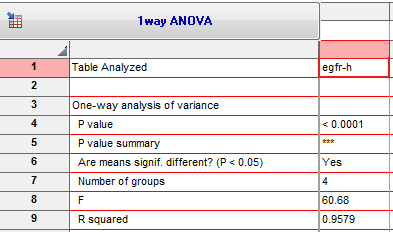
**

**
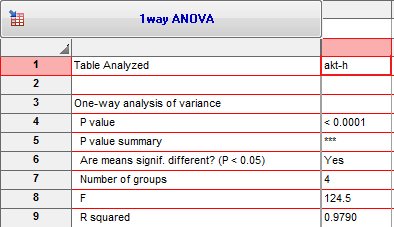
**

**
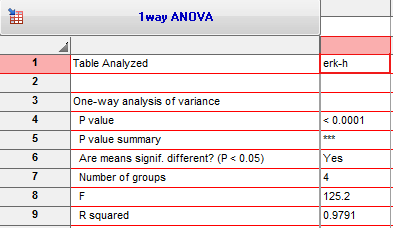
**

**(Figure 6(B))**

**
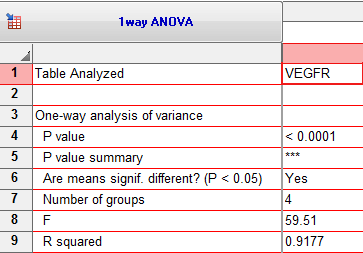
**

**(Figure 6(C))**

**
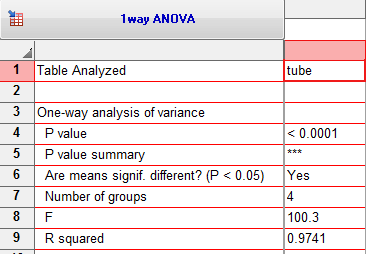
**

**
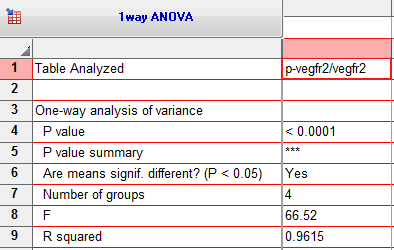
**
